# 1,4-Dihydropyridine Anions as Potent Single-Electron Photoreductants

**DOI:** 10.1021/acs.orglett.4c00513

**Published:** 2024-02-27

**Authors:** Prasadi C. Gallage, Mary G. McKee, Spencer P. Pitre

**Affiliations:** Department of Chemistry, Oklahoma State University, 107 Physical Sciences, Stillwater, Oklahoma 74078, United States

## Abstract

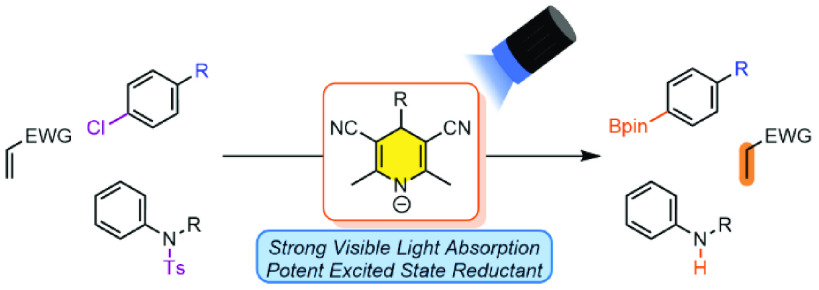

We report the use of simple 1,4-dihydropyridine anions as a general platform for promoting single-electron photoreductions. In the presence of a mild base, 1,4-dihydropyridines were shown to effectively promote the hydrodechlorination and borylation of aryl chlorides and the photodetosylation of N-tosyl aromatic amines under visible light irradiation. Our studies also demonstrate that the C4 substituent can influence the reactivity of these anions, reducing unwanted side reactions like hydrogen atom transfer and back-electron transfer.

1,4-Dihydropyridines (DHPs) have emerged as a valuable class of reagents in organic synthesis, often used as reductants in catalytic hydrogenation reactions and more recently as a class of sacrificial single-electron reductants or as the hydrogen atom source in photoredox catalyzed reactions.^1^ Further demonstrating the versatility of these reagents, 4-alkyl-1,4-DHPs have been extensively employed as precursors to alkyl radicals in C–C bond forming reactions.^2^ A common theme in these methodologies is the requirement for some chemical entity to oxidize the DHP to the radical cation to ultimately furnish the alkyl radical, which is normally achieved either by using a photocatalyst or through direct excitation of the DHP in the presence of an oxidant. In previous work from our lab, we demonstrated that, in the presence of a suitable base, 4-*tert*-alkyl-1,4-DHPs bearing cyano groups at C3 and C5 could be directly photolyzed under blue LED irradiation, which allowed for these DHPs to be used as *tert*-alkyl radical precursors in photochemical Giese reactions in the absence of a photocatalyst or external oxidant (Scheme 1A).^3^ Mechanistic studies suggested that the reaction proceeds via excitation of the DHP anion, which possesses a significantly red-shifted absorption in comparison to the neutral DHP. During the course of this work, we observed that 1,4-DHPs bearing secondary alkyl groups did not yield the corresponding Giese products, with hydrogenation of the Michael acceptor being predominantly observed in these cases.^3^ Inspired by this switch in mechanism afforded by a simple change in the substitution pattern at C4, we envisioned that these 1,4-DHPs, in the presence of a suitable mild base, could serve as a general platform for visible light mediated single-electron photoreduction reactions (Scheme 1B).^4^ Furthermore, as 1,4-DHPs are long established to be competent hydrogen atom donors,^1a^ this strategy would eliminate the need for separate chemical entities to promote the desired single-electron reduction and the subsequent hydrogen atom transfer, addressing a common drawback of many modern photochemical approaches in this space.

**Scheme 1:**
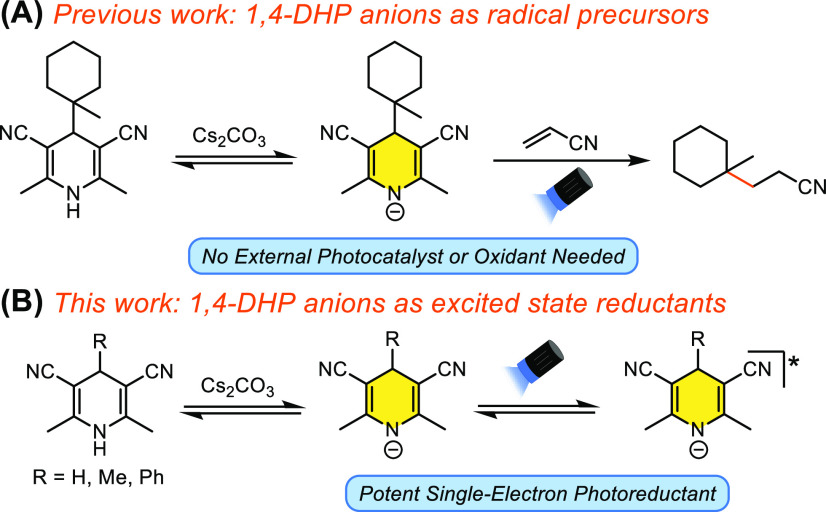
(A) Previous Work: 4-*tert*-Alkyl-1,4-DHP Anions as Radical Precursors. (B) This Work: 1,4-DHP Anions as Potent Single-Electron Photoreductants

Our design plan was further motivated by the recent reports of organic anions serving as potent single-electron photoreductants upon visible light irradiation.^5^ For example, Xia and co-workers, among others,^6^ have shown that phenolate anions possess highly reducing excited states upon photoexcitation with visible light (*E*_ox_* = −3.16 V vs SCE).^7^ Furthermore, Melchiorre and co-workers recently reported that thiolate anions generated from the deprotonation of cyclic thioamides possess highly reducing excited states (*E*_ox_^*^ = −3.38 V vs SCE) capable of activating C–Cl, C–F, and C–O bonds as well as promoting the Birch reduction of unfunctionalized arenes.^8^ Given this precedent, we set out to determine the excited state oxidation potentials of simple 1,4-DHP anions to assess their potential as potent single-electron photoreductants. UV–vis studies demonstrated that a significant red shift in the absorption of 1,4-DHP **I** is observed upon treatment with Cs_2_CO_3_, supporting the formation of the anion (Scheme 2). Excitation of the anion resulted in an emission centered at ∼490 nm, supporting the formation of an excited state upon irradiation of the anion. From these data, the *E*_0,0_ of 2.66 eV could be inferred from the crossing point between the absorption and emission spectra (466 nm).^9^ Using the ground state oxidation potential of 1,4-DHP **I** anion (*E*_p/2_ = 0.01 V vs SCE, see SI), an excited state oxidation potential [*E*(DHP^•^/[DHP^–^]*)] was estimated as −2.65 V vs SCE using the Rehm–Weller equation.^10^ Given the highly reducing nature of the excited state, we hypothesized that these 1,4-DHP anions could serve as a general platform for the single-electron photoreduction of C–Cl bonds of aryl halides^11^ as well as the photodetosylation of *N*-Ts aromatic amines.^12^

**Scheme 2:**
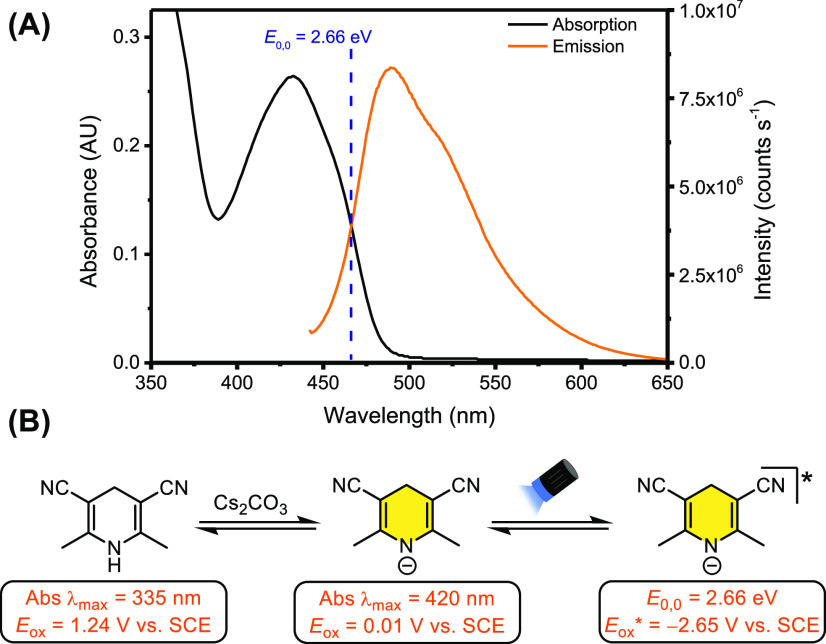
(A) Absorption (black trace) and Emission (orange trace) Spectra of the 1,4-DHP **I** Anion. (B) Summary of Electrochemical and Photophysical Data for the 1,4-DHP **I** Anion

We began our investigation of 1,4-DHP anions as potent photoreductants by studying the hydrodechlorination of methyl 4-chlorobenzoate (**1**) as a model system (Table 1). Gratifyingly, hydrodechlorinated product **2** was observed in 60% yield using only 1 equiv of 1,4-DHP **I** in the presence of 5 equiv of Cs_2_CO_3_ in MeCN under 456 nm LED irradiation (entry 1). Increasing the loading of 1,4-DHP **I** led to a further increase in yield (entries 2, 3). Finally, running the reaction under more dilute conditions was found to be beneficial, providing **2** in a 78% isolated yield (entry 4). Control experiments demonstrated that both Cs_2_CO_3_ and light were essential for promoting reactivity (entries 5, 6). Finally, we tested an alternative approach using Hantzsch ester (**II**) and *t*BuOK under 456 nm irradiation previously reported by Budén and co-workers,^13^ which afforded **2** in only 22% yield under comparable reaction conditions (entry 7).

**Table 1:**
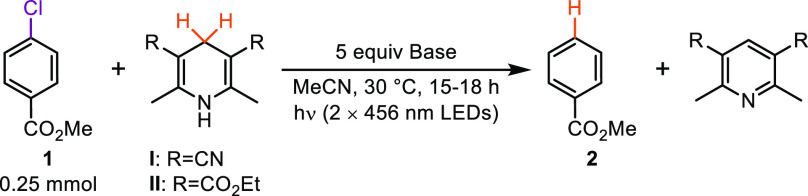
Hydrodechlorination Optimization and Control Reactions

entry	DHP	[MeCN] (mM)	base	yield of **2**^a^ (%)
1	**I**, 1.0 equiv	50	Cs_2_CO_3_	60
2	**I**, 1.2 equiv	50	Cs_2_CO_3_	69
3	**I**, 1.5 equiv	50	Cs_2_CO_3_	75
4	**I**, 1.5 equiv	62.5	Cs_2_CO_3_	78^b^
5	**I**, 1.5 equiv	62.5	none	c
6	**I**, 1.5 equiv	62.5	Cs_2_CO_3_	c,d
7	**II**, 1.5 equiv	62.5	*t*BuOK^e^	22

a Yield calculated by ^1^H NMR using 1,3,5-trimethoxybenzene as an external standard.

b Isolated yield.

c No reaction.

d No light.

e 2.2 equiv.

With the optimized conditions in hand, we examined the scope of compatible aryl chlorides (Scheme 3). To facilitate product isolation, we employed slightly modified conditions to afford the corresponding borylated products by adding bis(pinacolato)borane ((Bpin)_2_). For the borylation of aryl chlorides, it was also found that using 1,4-DHP **III** with a methyl group at C4 was beneficial, as it reduced the amount of hydrodechlorinated byproduct formed from an unwanted hydrogen atom transfer reaction (see SI for full reaction optimization). Aryl chlorides possessing electron-withdrawing groups (**3**–**5**) were efficiently photoreduced, giving the corresponding borylated products in moderate to good yields. Chlorobenzene (**6**) and 4-chlorobiphenyl (**7**) were also found to be compatible substrates, which was anticipated based on the measured reduction potential of **7** (*E*_p/2_ = −2.34 V vs SCE, see SI). Aryl chlorides bearing electron-donating groups did not undergo photoreduction, which was expected as the reduction potentials required exceeded the excited state oxidation potential of the 1,4-DHP **III** anion (*E*(DHP^•^/[DHP^–^]*) = −2.59 V vs SCE, see SI).^12a^

**Scheme 3:**
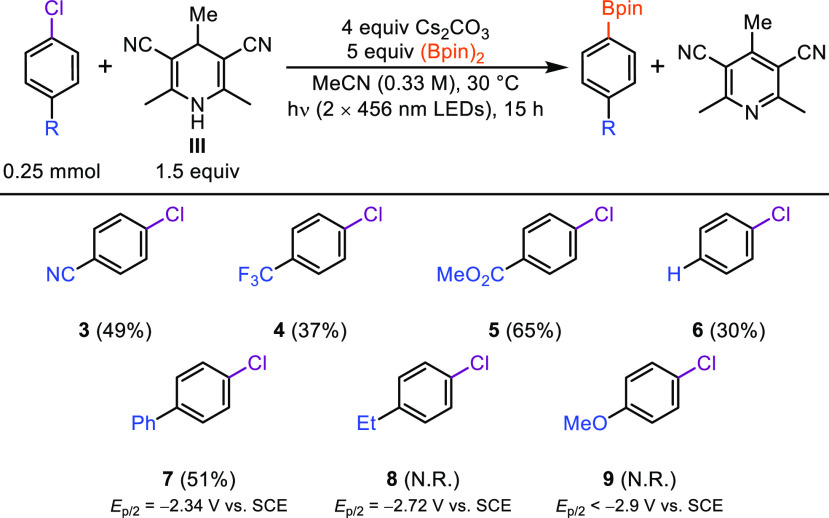
Reaction Scope for the Borylation of Aryl Chlorides Yields of isolated, purified products after 15 h of irradiation using the optimized conditions (see General Procedure C3 in the Supporting Information).

Next, we examined the potential of 1,4-DHP anions for the photodetosylation of *N*-Ts aromatic amines, given the similarities in the reported reduction potentials of these substrates in comparison to aryl chlorides.^12a,14^ As a model reaction, we examined the photodetosylation of *N*-Ts indole (**10**) using 1,4-DHP **I** as the excited state anionic photoreductant. In an initial attempt using only 1 equiv of DHP **I** in the presence of 5 equiv of Cs_2_CO_3_ in MeCN, detosylated indole **11** was observed in 72% yield after 18 h of 456 nm irradiation (Table 2, entry 1). Varying the loadings of DHP **I** and Cs_2_CO_3_ revealed that 1.2 equiv of DHP **I** and 4 equiv of Cs_2_CO_3_ were optimal, giving **11** in 99% yield (entries 2–4). Control reactions demonstrated that light and Cs_2_CO_3_ were essential for reactivity (entries 5 and 6). Finally, a time course revealed that the reaction was complete after 12 h, giving a 97% yield of **11** (entries 7 and 8).

**Table 2:**
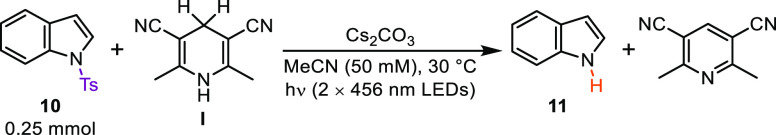
Photodetosylation Optimization and Control Reactions^a^

entry	equiv of DHP I	equiv of Cs_2_CO_3_	time (h)	yield of **11**^b^ (%)
1	1.0	5.0	18	72
2	1.2	5.0	18	81
3	1.5	5.0	18	83
4	1.2	4.0	18	99
5	1.2	0	18	c
6	1.2	4.0	18	c,d
7	1.2	4.0	8	89
8	1.2	4.0	12	97

a Ts: tosyl.

b Yields calculated by ^1^H NMR using 1,3,5-trimethoxybenzene as an external standard.

c No reaction.

d No light.

With the optimized conditions in hand, we explored the scope of compatible aromatic amines and heterocycles for the photodetosylation reaction (Scheme 4). *N*-Ts heterocycles such as indoles (**11**, **12**), benzimidazole (**13**), and carbazole (**14**) could all be photodetosylated in excellent yields using this method, and no loss in reactivity was observed when the detosylation was performed at 1 mmol scale. Secondary *N*-Ts anilines **15**–**17** were also effectively photodetosylated using this protocol. *N*-Ts melatonin (**18**) also underwent photodetosylation in 55% yield. Furthermore, 1,4-DHP **I** was able to mediate the selective deprotection of an *N*-Ts group in the presence of both Boc (**19**) and Cbz (**20**) protecting groups, highlighting the potential utility of this method for selective deprotections of aromatic amines in organic synthesis.

**Scheme 4:**
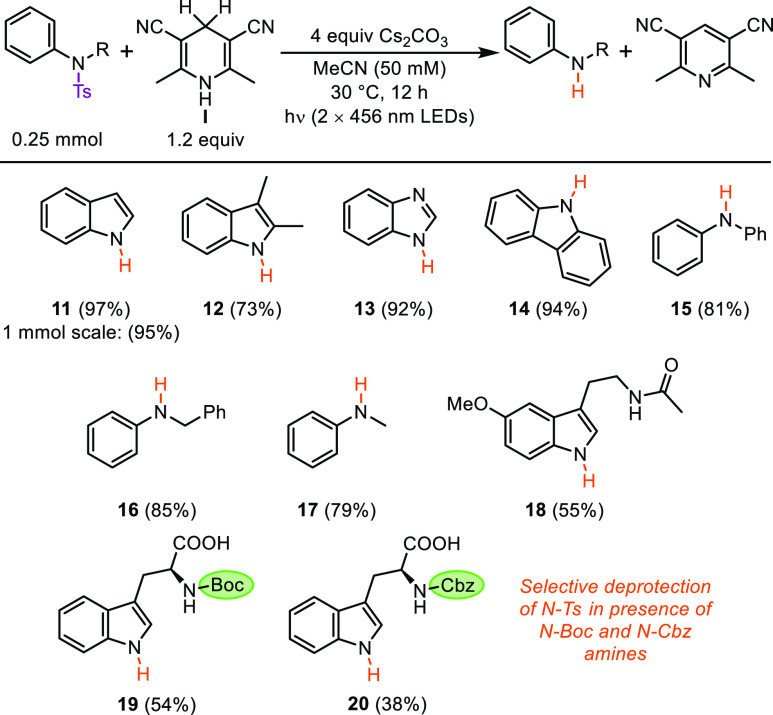
Reaction Scope for the Photodetosylation of *N*-Ts Aromatic Amines Yields of isolated, purified products after 12 h of irradiation using the optimized conditions (see General Procedure C4 in the Supporting Information).

While secondary *N*-Ts anilines underwent photodetosylation efficiently, photodetosylation of primary *N*-Ts aniline (**21**) was found to be significantly challenging under our standard conditions, despite the reaction being thermodynamically favorable based on the reported reduction potential of *N*-Ts aniline.^12a^ We posited that the decreased reactivity was attributed to the poor stability of the resulting anilide anion leaving group. After further optimization (see SI), we found that a reasonable yield (53%) of **21** could be obtained using 1,4-DHP **IV** with a phenyl group at C4 and using water as a cosolvent to stabilize the leaving group (Table 3). We initially posited that adding the phenyl group to the C4 position of the 1,4-DHP helped facilitate the initial single-electron transfer by enabling the preassociation of **IV** with the Ts group in the ground state;^15^ however, a similar level of complexation was observed between *N*-Ts aniline and the corresponding anions of 1,4-DHPs **I** and **IV** (see SI). We next considered that the substitution at C4 may influence the rate of back-electron transfer (BET), given that BET following substrate reduction is a common challenge in many photoredox systems.^16^ Therefore, we decided to examine the driving force for BET (Δ*G*_BET_) for the reaction of *N*-Ts aniline with the corresponding anions from 1,4-DHPs **I** and **IV**, where Δ*G*_BET_ is given by the negative of the energy stored in the radical (ion) pair, or *E*_ox_(donor) – *E*_red_(acceptor).^17^ Using these data, we calculated that Δ*G*_BET_ becomes more exothermic when employing the **IV** anion vs the parent **I** anion (−2.34 eV vs −2.02 eV, respectively). Given that the driving force in both these cases is so large, the BET reaction may occur in the Marcus inverted region, where the rate of BET decreases as Δ*G*_BET_ decreases.^18^ This is also in good agreement with previous reports from Gould and Farid, who demonstrated that the rates of BET decreased as the Δ*G*_BET_ decreased from −2 to −3 eV for different donor/acceptor systems.^17,19^ A similar effect was observed by Fukuzumi and co-workers for the photocatalytic oxidation of benzene to phenol.^20^ Given this precedent, we postulate that differences in BET rates, influenced by C4 substitution, may play a role in the increased reactivity for **IV** in the photodetosylation of *N*-Ts aniline. Finally, aliphatic *N*-Ts amines were found to be unreactive using our approach.

**Table 3:**
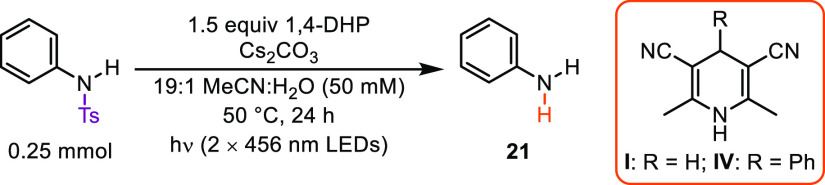
Photodetosylation of *N*-Ts Aniline

entry	DHP	equiv of Cs_2_CO_3_	Δ*G*_BET_ (eV)	yield of **21**^a^ (%)
1	**I**	5.0	–2.02	22
2	**IV**	5.0	–2.34	33
3	**IV**	2.0	–2.34	53^b^

a Yields calculated by ^1^H NMR using 1,3,5-trimethoxybenzene as an external standard.

b Isolated yield.

Given that this work was inspired by our previous observations that 1,4-DHP anions could mediate the hydrogenation of Michael acceptors under visible light irradiation,^3^ we briefly examined the ability of 1,4-DHP **I** anion to mediate these hydrogenations (Scheme 5). As anticipated, complete conversion of a range of Michael acceptors (**22**–**25**) was observed, giving the corresponding hydrogenated products in good yields. Given the propensity for Michael acceptors or other reducible functional groups such as aryl halides to undergo reduction in the presence of 1,4-DHPs under basic conditions, we believe the excited state reactivity of these anions should be carefully considered when developing and optimizing new transformations involving 1,4-DHPs.

**Scheme 5:**
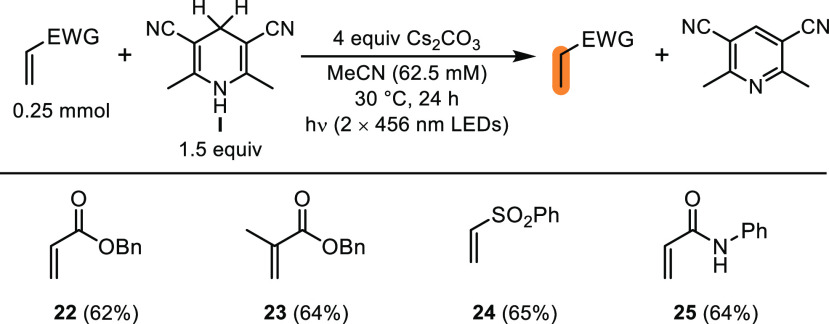
Reaction Scope for the Hydrogenation of Michael Acceptors Yields of isolated, purified products after 24 h of irradiation using the optimized conditions (see General Procedure C6 in the Supporting Information).

In summary, simple 1,4-DHPs, which can be deprotonated using a mild base to form the corresponding anion, can serve as a general platform for visible light mediated single-electron reductions. Through a combination of electrochemical and photophysical studies, the excited state oxidation potentials of 1,4-DHP anions were estimated to be approximately −2.6 V vs SCE, which was shown to be sufficiently reducing to promote the borylation of aryl chlorides and the photodetosylation of *N*-Ts aromatic amines. Furthermore, 1,4-DHP anions can effectively play the role of both a single-electron donor and hydrogen atom donor, as exemplified in the hydrodechlorination reaction of an aryl chloride. Our studies have also demonstrated that the C4 substituent of the 1,4-DHP can be used to influence the reactivity of the resulting excited state anion, reducing unwanted side reactions such as hydrogen atom transfer and back-electron transfer. Given the highly reducing excited states that can be accessed under mild conditions and the wide array of substrates that can be reduced at these potentials, we anticipate that 1,4-DHPs could find widespread use as photoreductants in organic synthesis.

## Data Availability

The data underlying this study are available in the published article and its Supporting Information.

## Supporting Information Available

Experimental procedures, reaction optimization, compound characterization, cyclic voltammetry studies, absorption and fluorescence studies and NMR spectra (PDF)
