# Supplementary material for: 1,4-Dihydropyridine Anions as Potent Single-Electron Photoreductants
Source: Org Lett. 2024 Feb 27;26(9):1975–9. doi: 10.1021/acs.orglett.4c00513 (PMC10928722; doi:10.1021/acs.orglett.4c00513)

Supporting Information

1,4-Dihydropyridine Anions as Potent Single-Electron Photoreductants

Prasadi C. Gallage, Mary G. McKee and Spencer P. Pitre*

Department of Chemistry, Oklahoma State University, 107 Physical Sciences, Stillwater, OK 74078, United States

*Email: spencer.p.pitre@okstate.edu

Table of Contents

**A.**	**General Information**	S2
	A1. Materials and Reagents	S2
	A2. Analytical Methods	S2
**B.**	**Optimization of Reaction Conditions**	S3
	B1. Hydrodechlorination of Methyl 4-Chlorobenzoate	S3
	B2. Borylation of Methyl 4-Chlorobenzoate	S4
	B3. Photodetosylation of *N*–Ts Indole	S5
	B4. Photodetosylation of *N*–Ts Aniline	S6
**C.**	**Experimental Procedures**	S8
	C1. General Procedure for Synthesis of 1,4-Dihydropyridines (DHPs)	S8
	C2. Procedure for Hydrodechlorination of Methyl 4-Chlorobenzoate	S8
	C3. General Procedure for Borylation of Aryl Chlorides	S9
	C4. General Procedure for Photodetosylation of *N*–Ts Aromatic Amines	S9
	C5. Procedure for Photodetosylation of *N*–Ts Aniline	S10
	C6. General Procedure for the Hydrogenation of Electron-Deficient Alkenes	S11
**D.**	**Picture of the Photochemical Reaction Set-Up**	S13
**E.**	**Characterization of Products**	S14
**F.**	**Electrochemical Measurements**	S25
**G.**	**Absorption and Emission Spectra**	S29
	G1. UV-Vis Studies	S29
	G2. EDA Complexation Studies	S30
	G3. Fluorescence Studies	S31
**H.**	**References**	S34
**I.**	**NMR Spectra**	S35

A. General Information

A1. Materials and Reagents

All reactions were conducted in oven-dried glassware under an inert atmosphere of argon, unless otherwise stated. All solvents and reagents were purchased from commercial suppliers (Fisher Scientific, TCI America, Sigma Aldrich, Oakwood Chemicals, Combi-Blocks Inc., Ambeed) and were used as received unless otherwise noted. Irradiation of reaction vessels was performed using two Kessil 40 W PR160-456 nm LEDs at a distance of ~6 cm. A fan was employed to ensure reactions remained at ~30 ^oC when using LEDs. Thin-layer chromatography (TLC) was conducted with silica gel 60 F254 pre-coated plates (0.25 μm thickness) and the analysis was performed using hexanes/EtOAc as the eluent and visualized by exposure to UV-light (254 nm) or potassium permanganate ($KMnO_4$) staining. Flash column chromatography was accomplished using a Biotage Isolera Four equipped with Sorbtech Purity flash column cartridges (60 Å porosity, 40-75 μm). Preparative thin layer chromatography was performed using Sorbtech silica gel prep TLC plates (w/UV254, glass backed, thickness: 1000 μm, dimensions: 20 x 20 cm).

A2. Analytical Methods

^1H NMR spectra were recorded on a 400 MHz spectrometer at 298 K and are reported relative to the signals for deuterated $CHCl_3$ (7.26 ppm). Data for ^1H NMR spectra are reported as follows: chemical shift (δ ppm), multiplicity, coupling constant, *J*, (reported in *Hz*) and integration. ^{13}C NMR spectra were recorded at 101 MHz. All ^{13}C NMR spectra were reported in terms of chemical shift in ppm relative to residual $CHCl_3$ (77.2 ppm) and were obtained with ^1H decoupling. High-resolution mass spectra were obtained with a quadrupole-Orbitrap hybrid mass spectrometer at Oklahoma State University. Absorption spectra were recorded on a Shimadzu UV-2600 UV-vis spectrometer and emission spectra were recorded on a Fluorolog3 - fluorescence spectrometer. IR spectra were recorded on a Shimadzu IRAffinity-1S FT-IR spectrophotometer equipped with a QATR 10 single reflectance ATR accessory and are reported in terms of frequency of absorption (cm^{-1}). Electrochemical studies were performed using a Pine WaveNow Potentiostat.

B. Optimization of Reaction Conditions

B1. Hydrodeclorination of Methyl 4-Chlorobenzoate

Table S1. Optimization of reaction conditions.

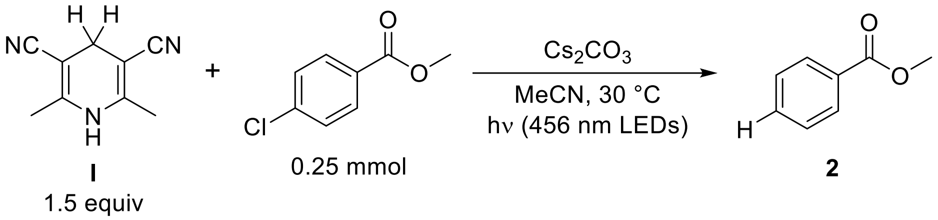

Entry	**I** (equiv)	MeCN (mL)	Cs_2CO_3 (equiv)	Time	% Yield **2**
1	1.0	5.0	5.0	18 h	60
2	1.2	5.0	5.0	18 h	69
3	1.5	5.0	5.0	18 h	75
4	2.0	5.0	5.0	18 h	77
5	1.5	3.0	5.0	18 h	70
6	1.5	4.0	5.0	18 h	77
7	1.5	4.0[a]	5.0	18 h	78
8	1.5	4.0	5.0[b]	18 h	31
9	1.5	4.0	1.0	18 h	50
10	1.5	4.0	4.0	18 h	66
11	1.5	4.0	5.0	4 h	65
12	1.5	4.0	5.0	8 h	72
13	1.5	4.0	5.0	10 h	74
14	1.5	4.0	5.0	12 h	75
15	**1.5**	**4.0**	**5.0**	**15 h**	**77**
16	1.5	4.0	-	15 h	0
17	1.5	4.0	5.0	15 h	0[c]

[a] Degassed with argon. [b] K_2CO_3 instead of C_2CO_3. [c] No light.

B2. Borylation of Methyl 4-Chlorobenzoate

Table S2. Optimization of reaction conditions.

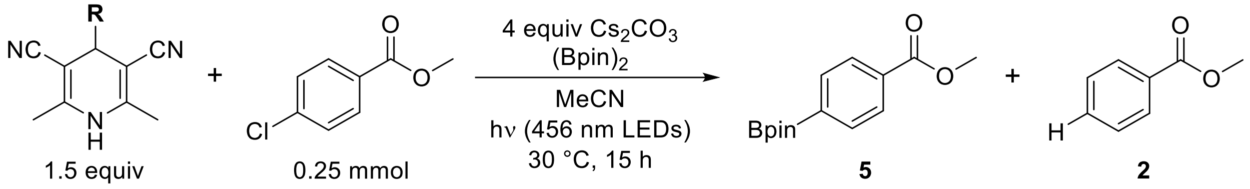

Entry	**R**	Solvent (mL)	$(Bpin)_2$ (equiv)	% Yield **5**	% Yield **2**
1	H	4.0	5	40	36
2	Me	4.0	2	35	36
3	Me	3.0	2	33	31
4	Me	3.0	5	42	16
5	Me	3.0	7	50	10
6	Me	2.0	5	45	16
7	Me	2.0	7	60	12
8	Me	1.0	5	50	15
9	Me	1.0	7	57	9
10	Me	0.75	2	40	33
11	**Me**	**0.75**	**5**	**68**	**12**
12	Me	0.5	5	47	16

B3. Photodetosylation of *N*–Ts Indole

Table S3. Optimization of reaction conditions.

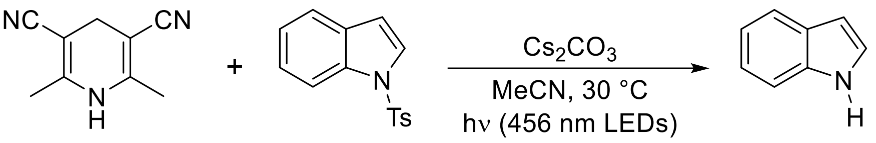

Entry	**I** (equiv)	MeCN (mL)	Cs_2CO_3 (equiv)	Time	% Yield **11**
1	1.5	3.0	5.0	18 h	74
2	1.5	4.0	5.0	18 h	80
3	1.5	5.0	5.0	18 h	83
4	1.5	6.0	5.0	18 h	84
5	1.0	5.0	5.0	18 h	72
6	1.1	5.0	5.0	18 h	75
7	1.2	5.0	5.0	18 h	81
8	1.2	5.0	1.0	18 h	66
9	1.2	5.0	2.0	18 h	72
10	1.2	5.0	3.0	18 h	76
11	1.2	5.0	4.0	18 h	99
12	1.2	5.0	4.0	8 h	89
13	1.2	5.0	4.0	10 h	91
14	**1.2**	**5.0**	**4.0**	**12 h**	**97**
15	1.2	5.0	-	18 h	0
16	1.2	5.0	4.0	8 h	0[a]

[a] No light.

B4. Photodetosylation of *N*–Ts Aniline

Table S4. Optimization of reaction conditions using DHP **I**.

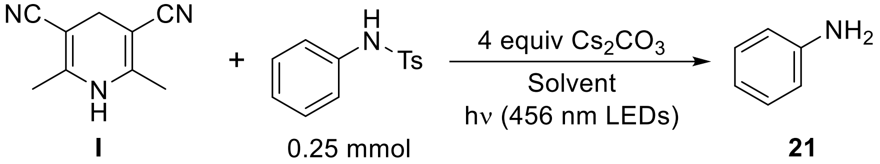

Entry	**I** (equiv)	Solvent (mL)	T (ºC)	Time	% Yield **21**
1	1.2	MeCN (5.0)	30	12 h	nd
2	1.2	6:1 MeCN:H_2O (5.0)	30	12 h	nd
3	1.2	6:1 MeCN:H_2O (5.0)	30	5 d	12
4	1.2	2:1 MeCN:H_2O (5.0)	50	24 h	<10
5	1.2	19:1 MeCN:H_2O (5.0)	50	24 h	15
6	**1.5**	**19:1 MeCN:H_2O (5.0)**	**50**	**24 h**	**22**
7	1.5	19:1 MeCN:H_2O (4.0)	50	24 h	19

Table S5. Optimization of reaction conditions using DHP **IV**.

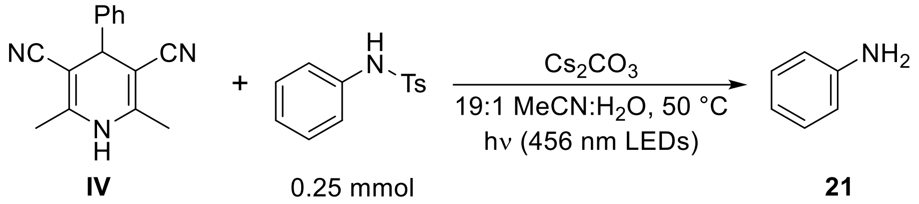

Entry	**IV** (equiv)	Solvent (mL)	Cs_2CO_3	Time	% Yield **21**
1	1.5	5.0	4 equiv	24 h	33
2	1.2	5.0	4 equiv	24 h	25
3	1.5	3.0	4 equiv	24 h	17
4	1.5	6.0	4 equiv	24 h	22
5	1.5	5.0	4 equiv	24 h	29
6	2.0	5.0	4 equiv	24 h	26
7	3.0	5.0	4 equiv	24 h	30
8	1.5	5.0	1 equiv	24 h	27
9	1.5	5.0	1.5 equiv	24 h	43
10	**1.5**	**5.0**	**2 equiv**	**24 h**	**58**
11	1.5	5.0	3 equiv	24 h	52
12	1.5	5.0	5 equiv	24 h	39
13	1.5	5.0	6 equiv	24 h	42
14	1.5	1.0	2 equiv	24 h	20
15	1.5	2.0	2 equiv	24 h	28
16	1.5	3.0	2 equiv	24 h	35
17	1.5	4.0	2 equiv	24 h	42
18	1.5	6.0	2 equiv	24 h	49
19	1.5	7.0	2 equiv	24 h	39

C. Experimental Procedures

C1. General Procedure for Synthesis of 1,4-Dihydropyridines (DHPs)

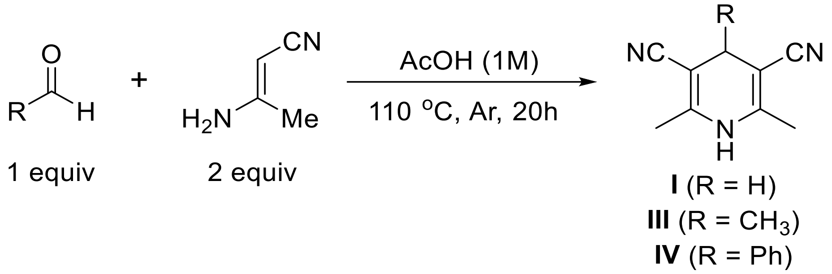

In an oven-dried round bottom flask with a PTFE-coated stir bar, the required aldehyde (1.0 equiv.), 3-aminocrotononitrile (2.0 equiv.) and glacial acetic acid (1.0 M) were charged under argon, and then the mixture was heated at 110 °C for 20 hours. The reaction was allowed to cool to room temperature, diluted with distilled water and extracted three times with EtOAc. The combined organic layers were washed with saturated $NaHCO_3$ and brine, dried over $MgSO_4$ and concentrated. The crude material was purified by flash column chromatography. The spectral data for **III** and **IV** agreed with those previously reported.[1,2]

Characterization for DHP I:

Physical State: Light-yellow solid. **^{1}H NMR** (400 MHz, $CDCl_3$) δ 5.65 (s, 1H), 3.20 (s, 2H), 2.03 (s, 6H). **^{13}C NMR** (101 MHz, $CDCl_3$) δ 146.72, 118.97, 80.16, 25.71, 18.73. **R_f:** 0.34 (1:1 Hex:EtOAc). **HRMS:** calculated **m/z** for $[M+H]^+$:160.0874, measured **m/z** for $[M+H]^+$:160.0869. **IR (neat, cm^{-1}):** 3293, 3251, 3133, 3015, 2872, 2200, 1670, 1514, 1388, 1282, 1014, 687.

C2. Hydrodechlorination of Methyl 4-Chlorobenzoate.

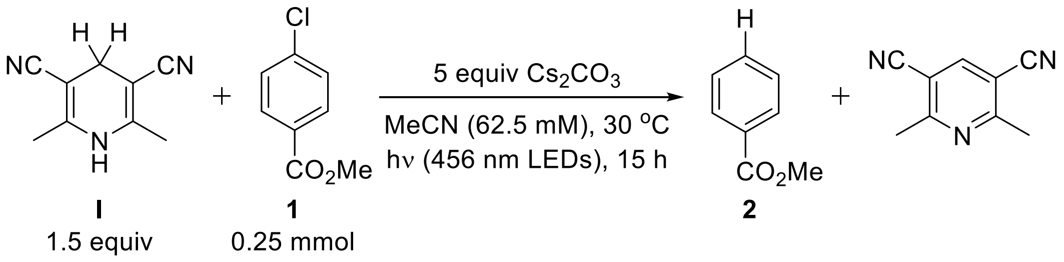

An oven-dried Pyrex tube (10 mL) equipped with a magnetic stir bar was charged with 1,4-DHP **I** (60 mg, 0.375 mmol, 1.5 equiv), methyl 4-chlorobenzoate (**1**) (43 mg, 0.25 mmol, 1 equiv) and Cs_2CO_3 (407 mg, 1.25 mmol, 5 equiv). Dry MeCN (dried over 3 Å molecular sieves) was added

(4.0 mL, 62.5 mM), and the reaction mixture was sonicated and then irradiated with two Kessil 40 W PR160-456 nm LEDs (~ 6 cm from reaction vessel) for 15 h at 30 °C. The reaction mixture was transferred into a separatory funnel and diluted with 15 mL of anhydrous ethyl ether and washed with 15 mL of brine. The aqueous phase was extracted with 15 mL of anhydrous ethyl ether. The combined organic phases were dried over $MgSO_4$, and the filtrate was concentrated *in vacuo*. The crude reaction mixture was purified by flash column chromatography using a Biotage Isolera Four. Yield is reported as isolated yield of the purified product **2**.

C3. General Procedure for Borylation of Aryl Chlorides.

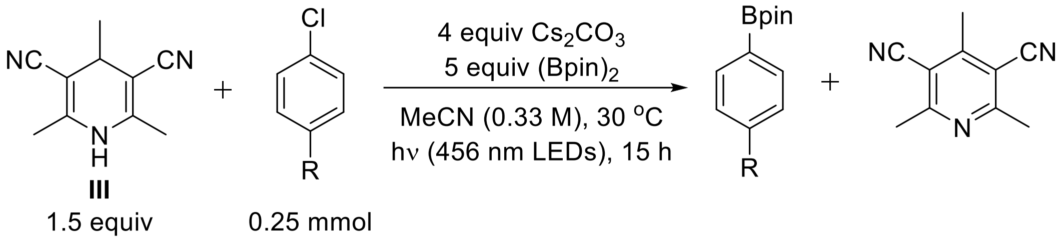

An oven-dried Pyrex tube (10 mL) equipped with a magnetic stir bar was charged with 1,4-DHP **III** (0.375 mmol, 1.5 equiv), 4-*R*-substituted chlorobenzene (0.25 mmol, 1 equiv), Cs_2CO_3 (407 mg, 1.25 mmol, 5 equiv) and bis(pinacolato)diboron (317 mg, 1.25 mmol, 5 equiv). Dry MeCN (dried over 3 Å molecular sieves) was added (0.75 mL, 0.33 M), and the reaction mixture was sonicated and then irradiated with two Kessil 40 W PR160-456 nm LEDs (~ 6 cm from reaction vessel) for 15 h at 30 °C. The reaction mixture was transferred into a separatory funnel and diluted with 10 mL of anhydrous ethyl ether and washed with 10 mL of brine. The aqueous phase was extracted with 10 mL of anhydrous ethyl ether. The combined organic phases were dried over $MgSO_4$, and the filtrate was concentrated *in vacuo*. The crude reaction mixture was purified by flash column chromatography using a Biotage Isolera Four. Yields were reported as isolated yield of the purified products.

C4. General Procedure for Photodetosylation of *N*-Ts Aromatic Amines.

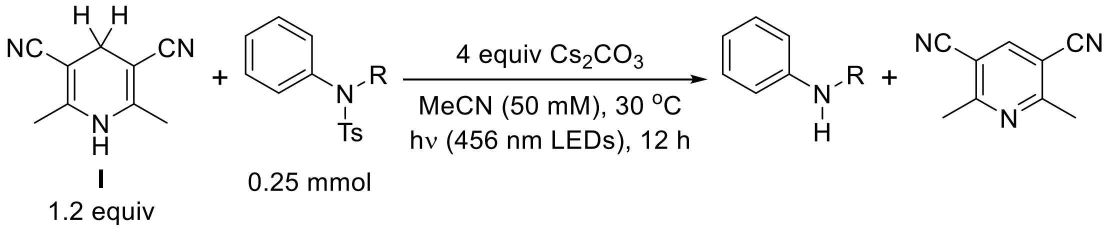

An oven-dried Pyrex tube (10 mL) equipped with a magnetic stir bar was charged with 1,4-DHP **I** (0.3 mmol, 1.2 equiv), *N*-tosyl aromatic amine (0.25 mmol, 1 equiv) and Cs_2CO_3 (326 mg, 1.0 mmol, 4 equiv). Dry MeCN (dried over 3 Å molecular sieves) was added (5.0 mL, 50 mM), and the reaction mixture was sonicated and then irradiated with two Kessil 40 W PR160-456 nm LEDs (~ 6 cm from reaction vessel) for 12 h at 30 °C. The reaction mixture was transferred into a separatory funnel and diluted with 15 mL of anhydrous ethyl ether and washed with 15 mL of brine. The aqueous phase was extracted with 15 mL of anhydrous ethyl ether. The combined organic phases were dried over $MgSO_4$, and the filtrate was concentrated *in vacuo*. The crude reaction mixture was purified by flash column chromatography using a Biotage Isolera Four. Yields were reported as isolated yield of the purified products.

C5. Procedure for Photodetosylation of *N*-Ts Aniline.

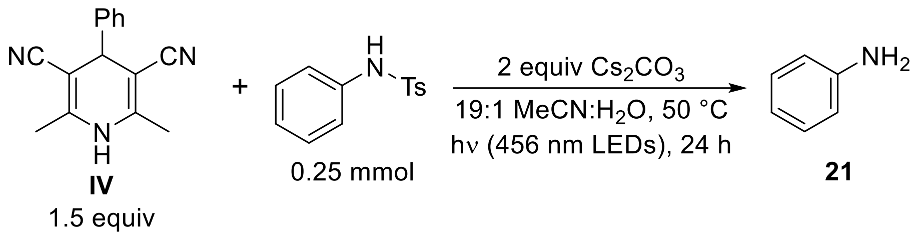

An oven-dried Pyrex tube (10 mL) equipped with a magnetic stir bar was charged with 1,4-DHP **IV** (88 mg, 0.375 mmol, 1.5 equiv), *N*-tosylaniline (62 mg, 0.25 mmol, 1 equiv) and Cs_2CO_3 (163 mg, 0.5 mmol, 2 equiv). A mixture of 19:1 MeCN: H_2O was added (5.0 mL, 50 mM), and the reaction mixture was sonicated and then irradiated with two Kessil 40 W PR160-456 nm LEDs (~ 6 cm from reaction vessel) for 24 h at 50 °C. The reaction mixture was transferred into a separatory funnel and diluted with 15 mL of anhydrous ethyl ether and washed with 15 mL of brine. The aqueous phase was extracted with 15 mL of anhydrous ethyl ether. The combined organic phases were dried over $MgSO_4$, and the filtrate was concentrated *in vacuo*. The crude reaction mixture was purified by flash column chromatography using a Biotage Isolera Four. Yield is reported as isolated yield of the purified product **21**.

C6. General Procedure for the Hydrogenation of Electron-Deficient Alkenes

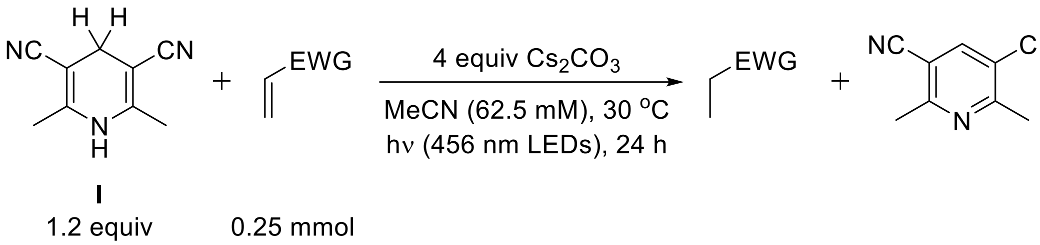

An oven-dried Pyrex tube (10 mL) equipped with a magnetic stir bar was charged with 1,4-DHP **I** (0.3 mmol, 1.2 equiv), electron-deficient alkene (0.25 mmol, 1 equiv) and Cs_2CO_3 (326 mg, 1.0 mmol, 4 equiv). Dry MeCN (dried over 3 Å molecular sieves) was added (4.0 mL, 62.5 mM), and the reaction mixture was sonicated and then irradiated with two Kessil 40 W PR160-456 nm LEDs (~ 6 cm from reaction vessel) for 24 h at 30 °C. The reaction mixture was transferred into a separatory funnel and diluted with 15 mL of anhydrous ethyl ether and washed with 15 mL of brine. The aqueous phase was extracted with 15 mL of anhydrous ethyl ether. The combined organic phases were dried over $MgSO_4$, and the filtrate was concentrated *in vacuo*. The crude reaction mixture was purified by flash column chromatography using a Biotage Isolera Four. Yields were reported as isolated yield of the purified products.

C7. Procedure for 1 mmol Scale Photodetosylation of *N*-Ts Indole

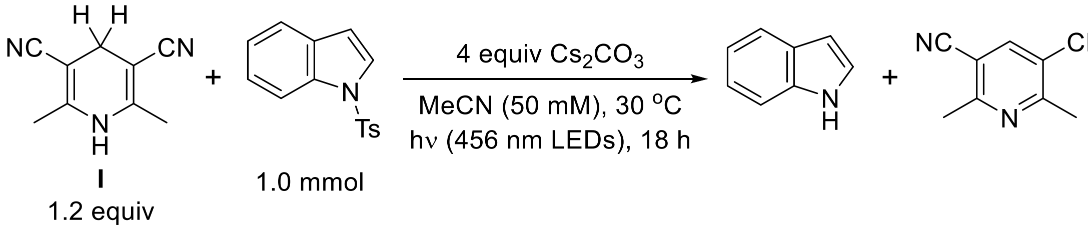

An oven-dried cylindrical pressure vessel (48 mL) equipped with a magnetic stir bar was charged with 1,4-DHP **I** (1.2 mmol, 1.2 equiv), *N*-tosyl indole (1.0 mmol, 1 equiv) and Cs_2CO_3 (1.3 g, 4.0 mmol, 4 equiv). Dry MeCN (dried over 3 Å molecular sieves) was added (20 mL, 50 mM), and the reaction mixture was sonicated and then irradiated with two Kessil 40 W PR160-456 nm LEDs (~ 6 cm from reaction vessel) for 18 h at 30 °C. The reaction mixture was transferred into a separatory funnel and diluted with 50 mL of anhydrous ethyl ether and washed with 50 mL of brine. The aqueous phase was extracted with 50 mL of anhydrous ethyl ether. The combined organic phases were dried over $MgSO_4$, and the filtrate was concentrated *in vacuo*. The crude

reaction mixture was purified by flash column chromatography using a Biotage Isolera Four. Yields were reported as isolated yield of the purified products.

D. Picture of the Photochemical Reaction Set-Up

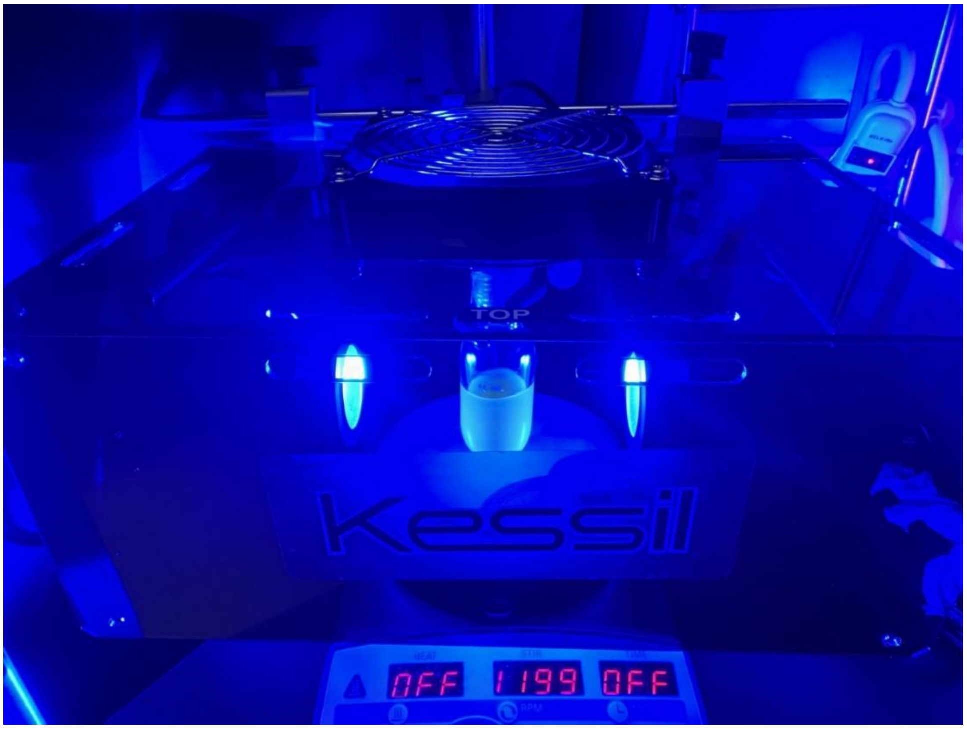

Figure S1. Picture of the photochemistry set-up employed in this work (1.0 mmol scale).

E. Characterization of Products

Methyl benzoate (2)

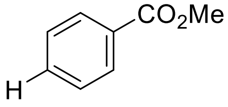

Prepared according to the procedure **C2** described above using **I** (60 mg, 0.38 mmol, 1.5 equiv), methyl 4-chlorobenzoate (**1**) (43 mg, 0.25 mmol, 1 equiv) and Cs_2CO_3 (407 mg, 1.25 mmol, 5 equiv) in 4 mL of dry MeCN. The crude material was purified by flash column chromatography (0 → 5% EtOAc in hexane) to afford the title compound as a white solid in 78% yield (27 mg). Spectral data are in accordance with those reported in the literature.

^{1}H NMR (400 MHz, $CDCl_3$) δ 8.10 – 8.01 (m, 2H), 7.61 – 7.52 (m, 1H), 7.48 – 7.39 (m, 2H), 3.92 (s, 3H). **^{13}C NMR** (101 MHz, $CDCl_3$) δ 167.3, 133.0, 130.3, 129.7, 128.5, 52.2.

R_f: 0.65 (4:1 Hex:EtOAc).

Reference: *Org. Lett.* **2011**, *13*, 5766-5769

4-(4,4,5,5-Tetramethyl-1,3,2-dioxaborolan-2-yl)benzonitrile (3)

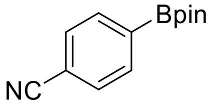

Prepared according to the general procedure **C3** described above using **III** (65 mg, 0.38 mmol, 1.5 equiv), 4-clorobenzonitrile (34 mg, 0.25 mmol, 1 equiv), Cs_2CO_3 (407 mg, 1.25 mmol, 5 equiv) and B_2pin_2 (317 mg, 1.25 mmol, 5 equiv) in 0.75 mL of dry MeCN. The crude material was purified by flash column chromatography (0 → 10% EtOAc in hexane) to afford the title compound as a white solid in 49% yield (28 mg). Spectral data are in accordance with those reported in the literature.

^{1}H NMR (400 MHz, $CDCl_3$) δ ^{1}H NMR (400 MHz, $CDCl_3$) δ 7.88 (d, *J* = 8.2 Hz, 2H), 7.64 (d, *J* = 8.1 Hz, 2H), 1.35 (s, 12H). **^{13}C NMR** (101 MHz, $CDCl_3$) δ 135.2, 131.3, 119.0, 114.7, 84.7, 77.4, 25.0. C_{ipso}-B not detected.

R_f: 0.59 (6:1 Hex:EtOAc).

Reference: *Chem. Eur. J.* **2021**, *27*, 15396-15405.

4,4,5,5-Tetramethyl-2-(4-(trifluoromethyl)phenyl)-1,3,2-dioxaborolane (4)

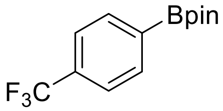

Prepared according to the general procedure **C3** described above using **III** (65 mg, 0.38 mmol, 1.5 equiv), 1-chloro-4-(trifluoromethyl)benzene (35 mg, 0.25 mmol, 1 equiv), Cs_2CO_3 (407 mg, 1.25 mmol, 5 equiv) and B_2pin_2 (317 mg, 1.25 mmol, 5 equiv) in 0.75 mL of dry MeCN. The crude material was purified by flash column chromatography (0 → 10% EtOAc in hexane) to afford the title compound as a white solid in 37% yield (25 mg). Spectral data are in accordance with those reported in the literature.

^{1}H NMR (400 MHz, $CDCl_3$) δ 7.91 (d, *J* = 7.9 Hz, 2H), 7.61 (d, *J* = 7.8 Hz, 2H), 1.36 (s, 12H). **^{13}C NMR** (101 MHz, $CDCl_3$) δ 135.0, 135.0, 124.7, 124.3, 84.3, 24.9. CF_3 not detected. **^{19}F NMR** (376 MHz, $CDCl_3$) δ -63.04. **R_f:** 0.60 (6:1 Hex:EtOAc).

Reference: *Chem. Eur. J.* **2021**, *27*, 15396-15405.

Methyl 4-(4,4,5,5-tetramethyl-1,3,2-dioxaborolan-2-yl)benzoate (5)

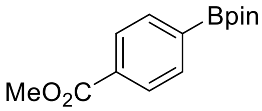

Prepared according to the general procedure **C3** described above using **III** (65 mg, 0.38 mmol, 1.5 equiv), methyl 4-chlorobenzoate (43 mg, 0.25 mmol, 1 equiv), Cs_2CO_3 (407 mg, 1.25 mmol, 5 equiv) and B_2pin_2 (317 mg, 1.25 mmol, 5 equiv) in 0.75 mL of dry MeCN. The crude material was purified by flash column chromatography (0 → 8% EtOAc in hexane) to afford the title compound as a white solid in 65% yield (43 mg). Spectral data are in accordance with those reported in the literature.

^{1}H NMR (400 MHz, $CDCl_3$) δ ^{1}H NMR (400 MHz, $CDCl_3$) δ 8.02 (d, *J* = 8.3 Hz, 2H), 7.87 (d, *J* = 8.1 Hz, 2H), 3.91 (s, 3H), 1.36 (s, 12H). **^{13}C NMR** (101 MHz, $CDCl_3$) δ 167.3, 159.3, 134.8, 132.5, 128.8, 84.3, 52.3, 25.0. **R_f:** 0.78 (1:1 Hex:EtOAc).

Reference: *Org. Lett.* **2011**, *13*, 5766-5769

4,4,5,5-Tetramethyl-2-phenyl-1,3,2-dioxaborolane (6)

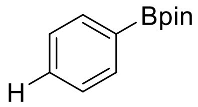

Prepared according to the general procedure **C3** described above using **III** (65 mg, 0.38 mmol, 1.5 equiv), methyl chlorobenzene (26 μL, 0.25 mmol, 1 equiv), Cs_2CO_3 (407 mg, 1.25 mmol, 5 equiv) and B_2pin_2 (317 mg, 1.25 mmol, 5 equiv) in 0.75 mL of dry MeCN. The crude material was purified by flash column chromatography (0 → 10% EtOAc in hexane) to afford the title compound as a white solid in 30% yield (16 mg). Spectral data are in accordance with those reported in the literature.

^{1}H NMR (400 MHz, $CDCl_3$) δ 7.81 (d, *J* = 6.5 Hz, 2H), 7.48 – 7.43 (m, 1H), 7.39 – 7.34 (m, 2H), 1.35 (s, 12H). **^{13}C NMR** (101 MHz, $CDCl_3$) δ 134.9, 131.4, 127.9, 83.9, 25.0. C_{ipso}-B not detected.

R_f: 0.58 (6:1 Hex:EtOAc).

Reference: *Chem. Eur. J.* **2021**, *27*, 15396-15405.

2-([1,1'-Biphenyl]-4-yl)-4,4,5,5-tetramethyl-1,3,2-dioxaborolane (7)

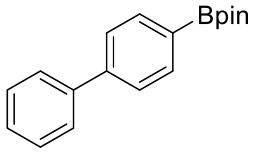

Prepared according to the general procedure **C3** described above using **III** (65 mg, 0.38 mmol, 1.5 equiv), methyl 4-chloro-1,1'-biphenyl (47 mg, 0.25 mmol, 1 equiv), Cs_2CO_3 (407 mg, 1.25 mmol, 5 equiv) and B_2pin_2 (317 mg, 1.25 mmol, 5 equiv) in 0.75 mL of dry MeCN. The crude material was purified by flash column chromatography (0 → 10% EtOAc in hexane) to afford the title compound as a white solid in 51% yield (36 mg). Spectral data are in accordance with those reported in the literature.

^{1}H NMR (400 MHz, $CDCl_3$) δ 7.89 (d, *J* = 8.1 Hz, 2H), 7.66 – 7.58 (m, 4H), 7.45 (t, *J* = 7.5 Hz, 2H), 7.39 – 7.33 (m, 1H), 1.37 (s, 12H). **^{13}C NMR** (101 MHz, $CDCl_3$) δ 144.0, 141.2, 135.4, 128.9, 127.7, 127.4, 126.6, 84.0, 25.0. **R_f:** 0.85 (4:1 Hex:EtOAc)

Reference: *Chem. Eur. J.* **2021**, *27*, 15396-15405.

1*H*-indole (11)

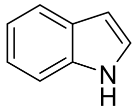

Prepared according to the general procedure **C4** described above using **I** (48 mg, 0.3 mmol, 1.2 equiv), *N*-Ts indole[3] (68 mg, 0.25 mmol, 1 equiv) and Cs_2CO_3 (326 mg, 1.00 mmol, 4 equiv) in 5 mL of dry MeCN. The crude material was purified by flash column chromatography (0 → 5% EtOAc in hexane) to afford the title compound as a white solid in 97% yield (28 mg). Spectral data are in accordance with those reported in the literature.

1 mmol scale reaction: Prepared according to procedure **C7** described above using **I** (192 mg, 1.2 mmol, 1.2 equiv), *N*-Ts indole[3] (271 mg, 1.0 mmol, 1 equiv) and Cs_2CO_3 (1.3 g, 4.0 mmol, 4 equiv) in 20 mL of dry MeCN. The crude material was purified by flash column chromatography (0 → 5% EtOAc in hexane) to afford the title compound as a white solid in 95% yield (134 mg). Spectral data are in accordance with those reported in the literature.

^{1}H NMR (400 MHz, $CDCl_3$) δ 8.12 (bs, 1H), 7.67 (d, *J* = 7.8 Hz, 1H), 7.41 (d, *J* = 8.1 Hz, 1H), 7.24 – 7.19 (m, 2H), 7.16 – 7.11 (m, 1H), 6.60 – 6.55 (m, 1H). **^{13}C NMR** (101 MHz, $CDCl_3$) δ 135.9, 128.0, 124.2, 122.1, 120.9, 120.0, 111.1, 102.8. **R_f:** 0.64 (4:1 Hex:EtOAc).

Reference: *Angew. Chem. Int. Ed.* **2020**, *59*, 1634-1643.

2,3-Dimethyl-1*H*-indole (12)

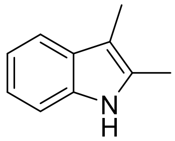

Prepared according to the general procedure **C4** described above using **I** (48 mg, 0.3 mmol, 1.2 equiv), *N*-Ts dimethylindole[3] (75 mg, 0.25 mmol, 1 equiv) and Cs_2CO_3 (326 mg, 1.00 mmol, 4 equiv) in 5 mL of dry MeCN. The crude material was purified by flash column chromatography (0 → 5% EtOAc in hexane) to afford the title compound as a white solid in 73% yield (26 mg). Spectral data are in accordance with those reported in the literature.

^{1}H NMR (400 MHz, $CDCl_3$) δ 7.67 (bs, 1H), 7.47 (d, *J* = 6.9 Hz, 1H), 7.28 – 7.22 (m, 1H), 7.14 – 7.05 (m, 2H), 2.37 (s, 3H), 2.23 (s, 3H). **^{13}C NMR** (101 MHz, $CDCl_3$) δ 135.3, 130.7, 129.6, 121.0, 119.1, 118.1, 110.1, 107.3, 11.7, 8.6. **R_f:** 0.55 (4:1 Hex:EtOAc).

Reference: *Tetrahedron Lett.* **2011**, *52*, 6758-6767.

1*H*-Benzo[*d*]imidazole (13)

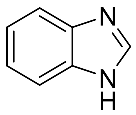

Prepared according to the general procedure **C4** described above using **I** (48 mg, 0.3 mmol, 1.2 equiv), *N*-Ts benzo[*d*]imidazole[3] (68 mg, 0.25 mmol, 1 equiv) and Cs_2CO_3 (326 mg, 1.00 mmol, 4 equiv) in 5 mL of dry MeCN. The crude material was purified by flash column chromatography (0 → 15% MeOH in DCM) to afford the title compound as a white solid in 92% yield (27 mg). Spectral data are in accordance with those reported in the literature.

^{1}H NMR (400 MHz, DMSO) δ 12.48 (bs, 1H), 8.22 (s, 1H), 7.65 – 7.53 (m, 2H), 7.23 – 7.14 (m, 2H). **^{13}C NMR** (101 MHz, DMSO-d_6) δ 142.1, 138.2, 121.9, 115.4.

R_f: 0.45 (5:1 DCM:MeOH).

Reference: *J. Mol. Struct.* **2012**, *1027*, 156-161.

9*H*-Carbazole (14)

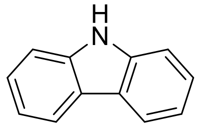

Prepared according to the general procedure **C4** described above using **I** (48 mg, 0.3 mmol, 1.2 equiv), *N*-Ts carbazole[4] (80 mg, 0.25 mmol, 1 equiv) and Cs_2CO_3 (326 mg, 1.00 mmol, 4 equiv) in 5 mL of dry MeCN. The crude material was purified by flash column chromatography (0 → 5% EtOAc in hexane) to afford the title compound as a white solid in 94% yield (39 mg). Spectral data are in accordance with those reported in the literature.

^{1}H NMR (400 MHz, $CDCl_3$) δ 8.09 (d, *J* = 6.0 Hz, 2H), 8.04 (bs, 1H), 7.47 – 7.39 (m, 4H), 7.29 – 7.19 (m, 2H). **^{13}C NMR** (101 MHz, $CDCl_3$) δ 139.6, 126.0, 123.5, 120.5, 119.6, 110.7. **R_f:** 0.44 (4:1 Hex:EtOAc).

Reference: *Org. Lett.* **2015**, *17*, 4372−4375.

Diphenylamine (15)

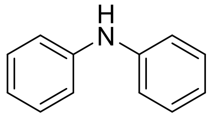

Prepared according to the general procedure **C4** described above using **I** (48 mg, 0.3 mmol, 1.2 equiv), *N*-Ts diphenylamine[3] (81 mg, 0.25 mmol, 1 equiv) and Cs_2CO_3 (326 mg, 1.00 mmol, 4 equiv) in 5 mL of dry MeCN. The crude material was purified by flash column chromatography (0 → 15% EtOAc in hexane) to afford the title compound as a colorless crystalline solid in 81% yield (34 mg). Spectral data are in accordance with those reported in the literature.

^{1}H NMR (400 MHz, $CDCl_3$) δ 7.33 – 7.19 (m, 3H), 7.11 – 7.03 (m, 4H), 6.98 – 6.88 (m, 2H), 5.67 (bs, 1H). **^{13}C NMR** (101 MHz, $CDCl_3$) δ 143.2, 129.5, 121.1, 117.9. **R_f:** 0.61 (4:1 Hex:EtOAc).
Reference: *J. Organomet. Chem.* **2017**, *841*, 57-61.

***N*-Benzylaniline (16)**

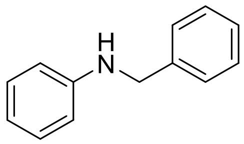

Prepared according to the general procedure **C4** described above using **I** (48 mg, 0.3 mmol, 1.2 equiv), *N*-Ts benzylaniline[3] (84 mg, 0.25 mmol, 1 equiv) and Cs_2CO_3 (326 mg, 1.00 mmol, 4 equiv) in 5 mL of dry MeCN. The crude material was purified by flash column chromatography (0 → 10% EtOAc in hexane) to afford the title compound as a colorless liquid in 85% yield (39 mg). Spectral data are in accordance with those reported in the literature.

^{1}H NMR (400 MHz, $CDCl_3$) δ ^{1}H NMR (400 MHz, $CDCl_3$) δ 7.43 – 7.34 (m, 4H), 7.33 – 7.27 (m, 1H), 7.23 – 7.15 (m, 2H), 6.74 (t, *J* = 7.3 Hz, 1H), 6.66 (d, *J* = 7.5 Hz, 2H), 4.35 (s, 2H), 4.04 (bs, 1H). **^{13}C NMR** (101 MHz, $CDCl_3$) δ 148.3, 139.6, 129.4, 128.8, 127.6, 127.4, 117.7, 113.0, 48.5. **R_f:** 0.75 (4:1 Hex:EtOAc).

Reference: *Adv. Synth. Catal.* **2019**, *361*, 3800-3806.

***N*-Methylaniline (17)**

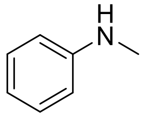

Prepared according to the general procedure **C4** described above using **I** (48 mg, 0.3 mmol, 1.2 equiv), *N*-Ts methylaniline[3] (65 mg, 0.25 mmol, 1 equiv) and Cs_2CO_3 (326 mg, 1.00 mmol, 4 equiv) in 5 mL of dry MeCN. The crude material was purified by flash column chromatography (0 → 8% EtOAc in hexane) to afford the title compound as a yellow oil in 79% yield (21 mg). Spectral data are in accordance with those reported in the literature.

^{1}H NMR (400 MHz, $CDCl_3$) δ 7.23 – 7.15 (m, 2H), 6.74 – 6.68 (m, 1H), 6.65 – 6.58 (m, 2H), 3.69 (bs, 1H), 2.84 (s, 3H). **^{13}C NMR** (101 MHz, $CDCl_3$) δ 149.5, 129.3, 117.4, 112.6, 30.9. **R_f:** 0.46 (4:1 Hex:EtOAc).

Reference: *Adv. Synth. Catal.* **2019**, *361*, 3800-3806.

***N*-(2-(5-Methoxy-1*H*-indol-3-yl)ethyl)acetamide (18)**

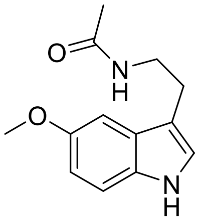

Prepared according to the general procedure **C4** described above using **I** (48 mg, 0.3 mmol, 1.2 equiv), *N*-Ts (2-(5-methoxyindol-3-yl)ethyl)acetamide[3] (115 mg, 0.25 mmol, 1 equiv) and Cs_2CO_3 (326 mg, 1.00 mmol, 4 equiv) in 5 mL of dry MeCN. The crude material was purified by flash column chromatography (0 → 20% MeOH in DCM) to afford the title compound as a white solid in 55% yield (33 mg). Spectral data are in accordance with those reported in the literature.

^{1}H NMR (400 MHz, $CDCl_3$) δ 8.02 (bs, 1H), 7.28 (s, 1H), 7.04 (d, J = 2.4 Hz, 1H), 7.02 (s, 1H), 6.88 (dd, J = 8.8, 2.4 Hz, 1H), 5.64 (bs, 1H), 3.86 (s, 3H), 3.60 (q, J = 6.5 Hz, 2H), 2.95 (t, J = 6.7 Hz, 2H), 1.94 (s, 3H). **^{13}C NMR** (101 MHz, $CDCl_3$) δ 170.4, 154.3, 131.7, 127.9, 122.9, 112.9, 112.7, 112.2, 100.6, 56.1, 39.9, 25.4, 23.5. **R_f:** 0.80 (3:2 DCM:MeOH).

Reference: *Nat. Commun*. **2022**, *13*, 7961-7970.

(*tert*-Butoxycarbonyl)tryptophan (19)

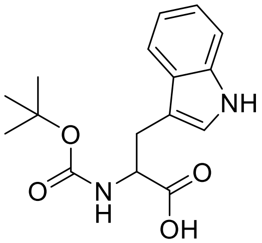

Prepared according to the general procedure **C4** described above using **I** (48 mg, 0.3 mmol, 1.2 equiv), *N*-Ts (*tert*-butoxycarbonyl)tryptophan[5] (115 mg, 0.25 mmol, 1 equiv) and Cs_2CO_3 (326 mg, 1.00 mmol, 4 equiv) in 5 mL of dry MeCN. The crude material was purified by flash column chromatography (0 → 30% EtOAc in hexane) to afford the title compound as a white solid in 54% yield (41 mg). Spectral data are in accordance with those reported in the literature.

^{1}H NMR (400 MHz, Acetone-d_6) δ 10.07 (bs, 1H), 7.62 (d, *J* = 7.8 Hz, 1H), 7.38 (d, *J* = 8.0 Hz, 1H), 7.26 – 7.20 (m, 1H), 7.13 – 7.06 (m, 1H), 7.06 – 6.99 (m, 1H), 5.88 (d, *J* = 8.2 Hz, 1H), 4.55 – 4.39 (m, 1H), 3.40 – 3.09 (m, 3H), 1.37 (s, 9H). **^{13}C NMR** (101 MHz, Acetone-d_6) δ 173.9, 156.2, 137.6, 128.7, 124.3, 122.1, 119.6, 119.3, 112.2, 111.2, 79.2, 55.2, 28.5, 28.2. **R_f:** 0.48 (4:1 DCM:MeOH)

Reference: *ACS Catal.* **2017**, *7*, 6821-6826.

((Benzyloxy)carbonyl)tryptophan (20)

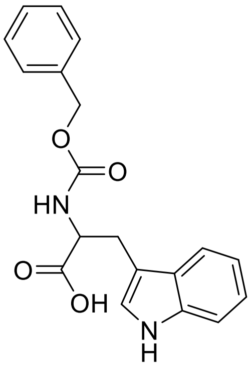

Prepared according to the general procedure **C4** described above using **I** (48 mg, 0.3 mmol, 1.2 equiv), *N*-Ts ((benzyloxy)carbonyl)tryptophan[6] (123 mg, 0.25 mmol, 1 equiv) and Cs_2CO_3 (326 mg, 1.00 mmol, 4 equiv) in 5 mL of dry MeCN. The crude material was purified by flash column chromatography (0 → 50% EtOAc in hexane) to afford the title compound as a white solid in 38% yield (32 mg). Spectral data are in accordance with those reported in the literature.

^{1}H NMR (400 MHz, CD_3OD) δ 10.26 (bs, 1H), 7.56 (d, *J* = 8.0 Hz, 1H), 7.35 – 7.23 (m, 6H), 7.16 – 7.05 (m, 3H), 7.03 – 6.95 (m, 1H), 5.08 – 4.98 (m, 2H), 4.54 – 4.46 (m, 1H), 3.37 – 3.33 (m, 1H), 3.19 – 3.04 (m, 1H). **^{13}C NMR** (101 MHz, CD_3OD) δ 175.7, 158.4, 138.2, 138.0, 129.4, 128.9, 128.8, 128.7, 124.5, 122.4, 119.8, 119.3, 112.2, 111.1, 67.5, 56.4, 28.7. **R_f:** 0.45 (4:1 DCM:MeOH)

Reference: *Bioorg. Med. Chem. Lett.* **2016**, *26*, 5000-5006.

Aniline (21)

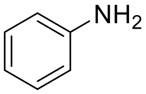

Prepared according to procedure **C5** described above using **IV** (88 mg, 0.38 mmol, 1.5 equiv), *N*-Ts aniline[3] (62 mg, 0.25 mmol, 1 equiv) and Cs_2CO_3 (163 mg, 0.50 mmol, 2 equiv) in 5 mL of dry MeCN. The crude material was purified by flash column chromatography (0 → 20% EtOAc in hexane) to afford the title compound as a light-yellow oil in 53% yield (12 mg). Spectral data are in accordance with those reported in the literature.

^{1}H NMR (400 MHz, $CDCl_3$) δ 7.20 – 7.11 (m, 2H), 6.76 (t, *J* = 7.4 Hz, 1H), 6.69 (d, *J* = 7.3 Hz, 2H), 3.64 (s, 2H). **^{13}C NMR** (101 MHz, $CDCl_3$) δ 146.5, 129.4, 118.7, 115.2. **R_f:** 0.36 (4:1 Hex:EtOAc).

Reference: *ACS Catal.* **2022**, *12*, 24, 15590-15599.

Benzyl propionate (22)

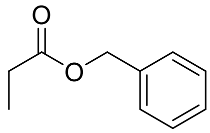

Prepared according to procedure **C6** described above using **I** (48 mg, 0.3 mmol, 1.2 equiv), Benzyl acrylate (41 μL, 0.25 mmol, 1 equiv) and Cs_2CO_3 (326 mg, 1.00 mmol, 4 equiv) in 4 mL of dry MeCN. The crude material was purified by flash column chromatography (0 → 10% EtOAc in hexane) to afford the title compound as a colorless oil in 62% yield (26 mg). Spectral data are in accordance with those reported in the literature.

^{1}H NMR (400 MHz, $CDCl_3$) δ 7.41 – 7.28 (m, 5H), 5.12 (s, 2H), 2.39 (q, *J* = 7.6 Hz, 2H), 1.17 (t, *J* = 7.6 Hz, 3H). **^{13}C NMR** (101 MHz, $CDCl_3$) δ 174.5, 136.3, 128.7, 128.5, 128.3, 66.3, 27.7, 9.2. **R_f:** 0.82 (1:1 Hex:EtOAc).
Reference: *Tetrahedron* **2005,** *61*, 2217-2231.

Benzyl isobutyrate (23)

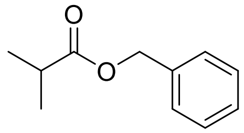

Prepared according to procedure **C6** described above using **I** (48 mg, 0.3 mmol, 1.2 equiv), Benzyl methacrylate (42 μL, 0.25 mmol, 1 equiv) and Cs_2CO_3 (326 mg, 1.00 mmol, 4 equiv) in 4 mL of dry MeCN. The crude material was purified by flash column chromatography (0 → 10% EtOAc in hexane) to afford the title compound as a colorless oil in 64% yield (29 mg). Spectral data are in accordance with those reported in the literature.
^{1}H NMR (400 MHz, $CDCl_3$) δ 7.40 – 7.28 (m, 5H), 5.12 (s, 2H), 2.69 – 2.51 (m, 1H), 1.20 (s, 3H), 1.19 (s, 3H). **^{13}C NMR** (101 MHz, $CDCl_3$) δ 177.1, 136.4, 128.7, 128.2, 128.1, 66.2, 34.2, 19.1. **R_f:** 0.66 (4:1 Hex:EtOAc).
Reference: *Tetrahedron* **2005,** *61*, 2217-2231.

(Ethylsulfonyl)benzene (24)

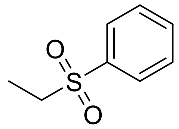

Prepared according to procedure **C6** described above using **I** (48 mg, 0.3 mmol, 1.2 equiv), (Vinylsulfonyl)benzene (42 mg, 0.25 mmol, 1 equiv) and Cs_2CO_3 (326 mg, 1.00 mmol, 4 equiv) in 4 mL of dry MeCN. The crude material was purified by flash column chromatography (0 → 10% EtOAc in hexane) to afford the title compound as a colorless oil in 65% yield (28 mg). Spectral data are in accordance with those reported in the literature.

^{1}H NMR (400 MHz, $CDCl_3$) δ 7.94 – 7.88 (m, 2H), 7.70 – 7.63 (m, 1H), 7.61 – 7.53 (m, 2H), 3.12 (q, *J* = 7.4 Hz, 2H), 1.28 (t, *J* = 7.4 Hz, 3H). **^{13}C NMR** (101 MHz, $CDCl_3$) δ 138.7, 133.8, 129.4, 128.4, 50.7, 7.6. **R_f:** 0.63 (1:1 Hex:EtOAc).
Reference: *J. Am. Chem. Soc.* **2021**, *143*, 25, 9657-9663.

N-phenylpropionamide (25)

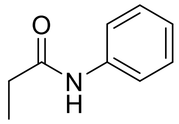

Prepared according to procedure **C6** described above using **I** (48 mg, 0.3 mmol, 1.2 equiv), *N*-phenylacrylamide (37 mg, 0.25 mmol, 1 equiv) and Cs_2CO_3 (326 mg, 1.00 mmol, 4 equiv) in 4 mL of dry MeCN. The crude material was purified by flash column chromatography (0 → 10% EtOAc in hexane) to afford the title compound as a white solid in 64% yield (24 mg). Spectral data are in accordance with those reported in the literature.
^{1}H NMR (400 MHz, $CDCl_3$) δ 7.51 (d, *J* = 7.9 Hz, 2H), 7.33 (t, *J* = 7.8 Hz, 2H), 7.16 (s, 1H), 7.10 (t, *J* = 7.4 Hz, 1H), 2.39 (q, *J* = 7.5 Hz, 2H), 1.25 (t, *J* = 7.5 Hz, 3H). **^{13}C NMR** (101 MHz, $CDCl_3$) δ 172.1, 138.1, 129.2, 124.3, 119.9, 30.9, 9.8. **R_f:** 0.54 (1:1 Hex:EtOAc).
Reference: *Tetrahedron* **2009,** *65*, 7790-7793.

F. Electrochemical Measurements

Cyclic voltammograms and electrochemical potentials were obtained with a Pine WaveNow Potentiostat. All the samples were prepared with 0.3 mmol of substrate in 30 mL of 0.1 M tetra-n-butylammonium hexafluorophosphate in dry, degassed acetonitrile. Measurements were taken using a glassy carbon working electrode (3 mm diameter, 0.07 cm^2), a platinum wire counter electrode, and a 4.0 M KCl Ag/AgCl reference electrode. The working electrode was polished using a 0.05 μm alumina slurry on microcloth before use. All measurements were performed at room temperature. Reduction of 4-chlorobiphenyl was measured by beginning with an initial potential of −1.0 V vs. Ag/AgCl (reductive scan) with a switch in potential at −2.50 V and a scan rate of 100 mV/s. Oxidations of 1,4-DHPs and their anions were measured by beginning with an initial potential of +1.00 V vs. Ag/AgCl (oxidative scan) with switching the potential at +1.50 V and a scan rate of 100 mV/s. Solutions of 1,4-DHP anions were prepared using 0.33 mmol (1.1 equiv) of KO^{t}Bu. The glassy carbon working electrode was cleaned between each scan. Data was analyzed using AFTERMATH software by identifying the maximum current (Cp) and determining the potential (Ep/2) at half of the maximum current (Cp/2). The obtained value was referenced to Ag/AgCl and converted to SCE by subtracting 0.03 V.

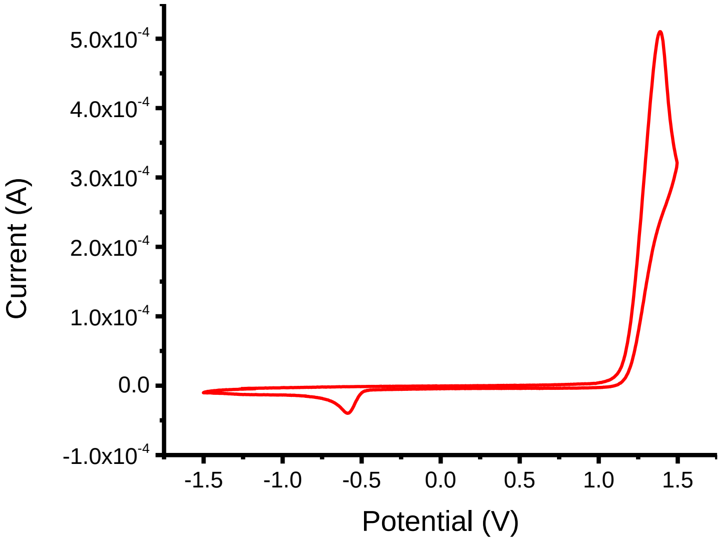

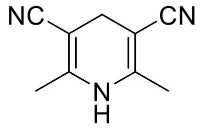

$E^p_{1/2}$ = 1.27 V (1.24 V vs. SCE)

Figure S2. Cyclic voltammogram of 1,4-DHP **I** using the IUPAC plotting convention. Starting point: +1.0 V, oxidative scan.

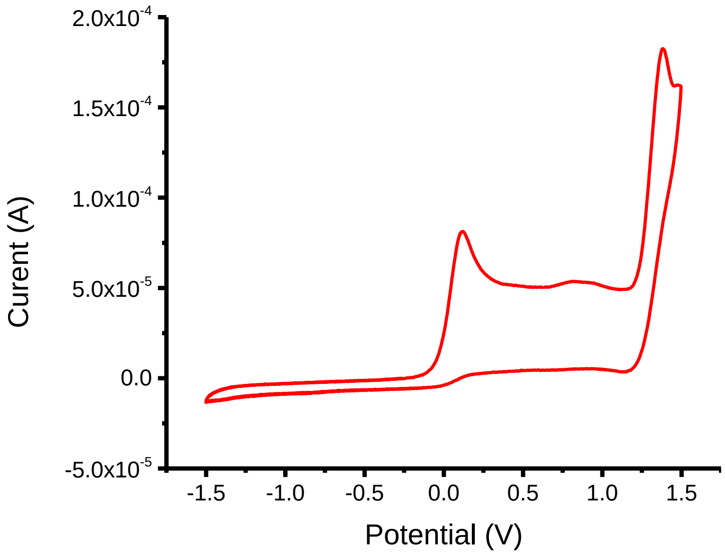

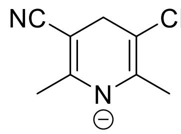

$E^{p}_{1/2}$ = 0.04 V (0.01 V vs. SCE)

Figure S3. Cyclic voltammogram of 1,4-DHP **I** + KO^{t}Bu using the IUPAC plotting convention. Starting point: +1.0 V, oxidative scan.

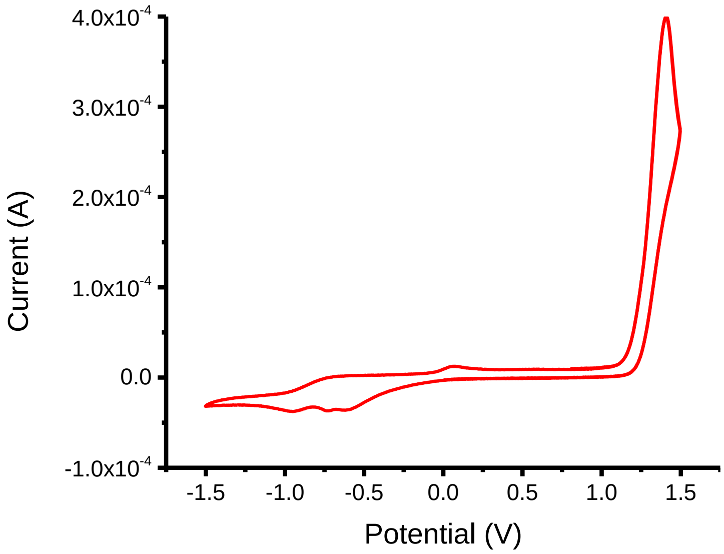

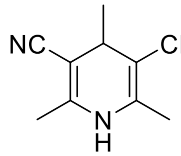

$E^{p}_{1/2}$ = 1.30 V (1.27 V vs. SCE)

Figure S4. Cyclic voltammogram of 1,4-DHP **III** using the IUPAC plotting convention. Starting point: +1.0 V, oxidative scan.

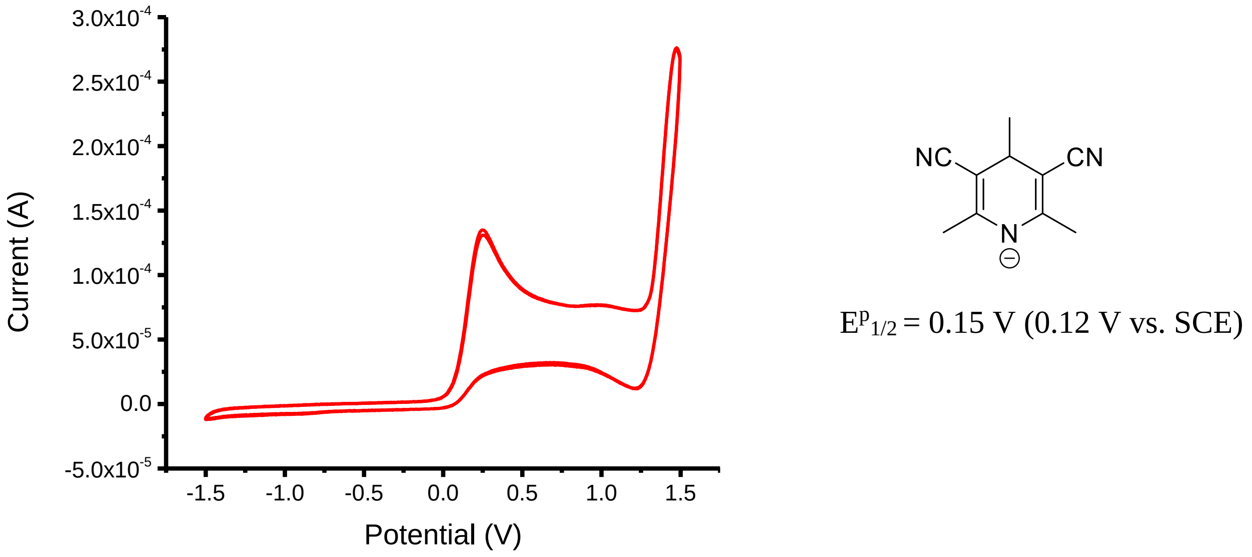

Figure S5. Cyclic voltammogram of 1,4-DHP **III** + KO^{t}Bu using the IUPAC plotting convention. Starting point: +1.0 V, oxidative scan.

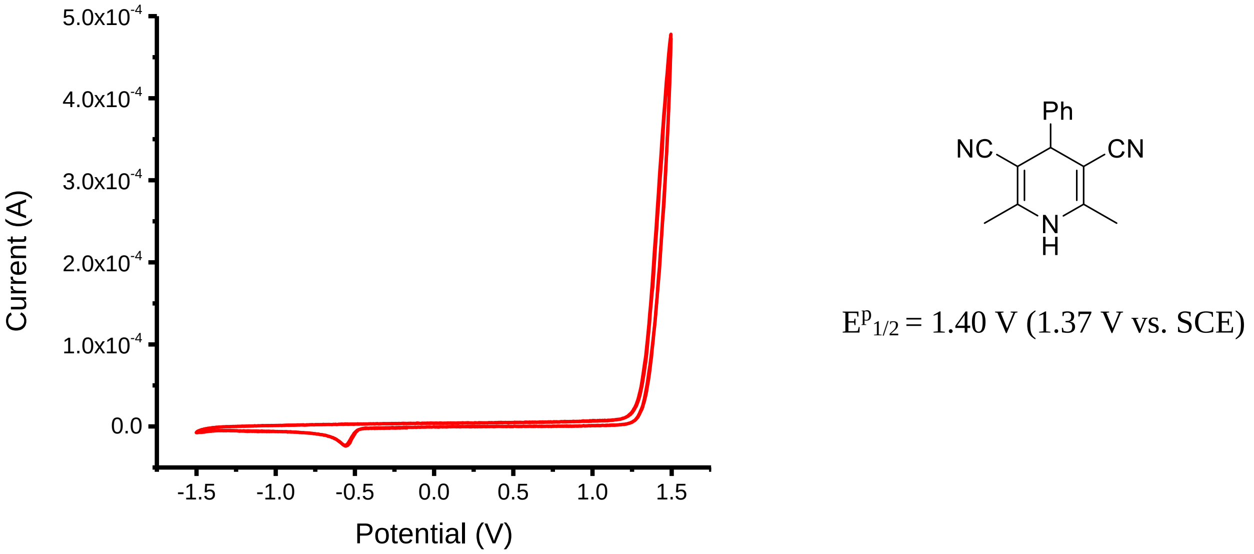

Figure S6. Cyclic voltammogram of 1,4-DHP **IV** using the IUPAC plotting convention. Starting point: +1.0 V, oxidative scan.

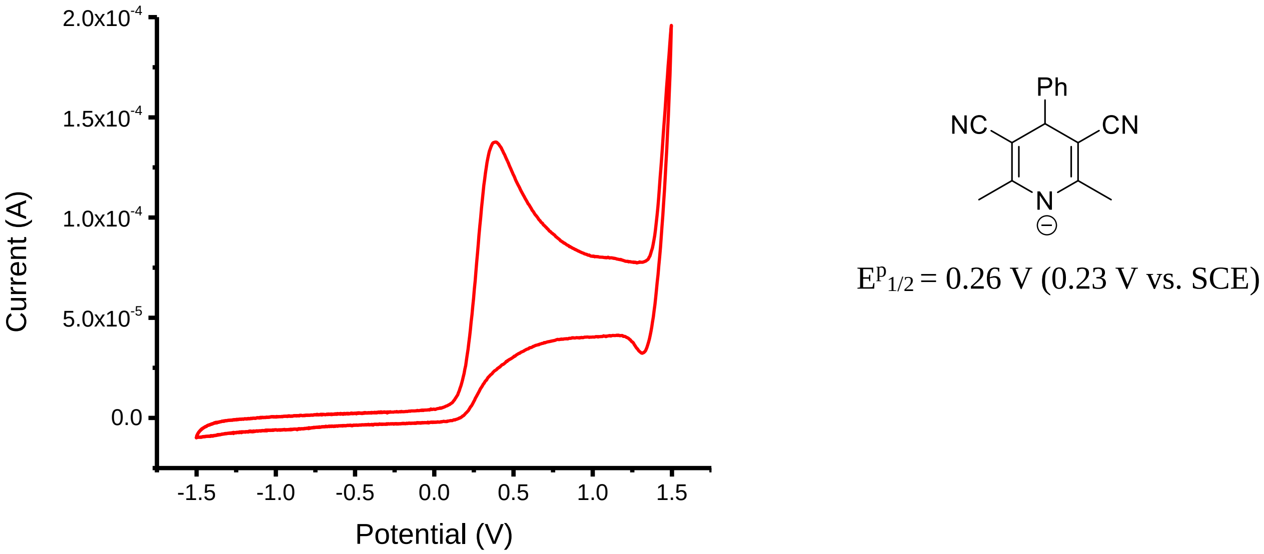

Figure S7. Cyclic voltammogram of 1,4-DHP **IV** + KO^{t}Bu using the IUPAC plotting convention. Starting point: +1.0 V, oxidative scan.

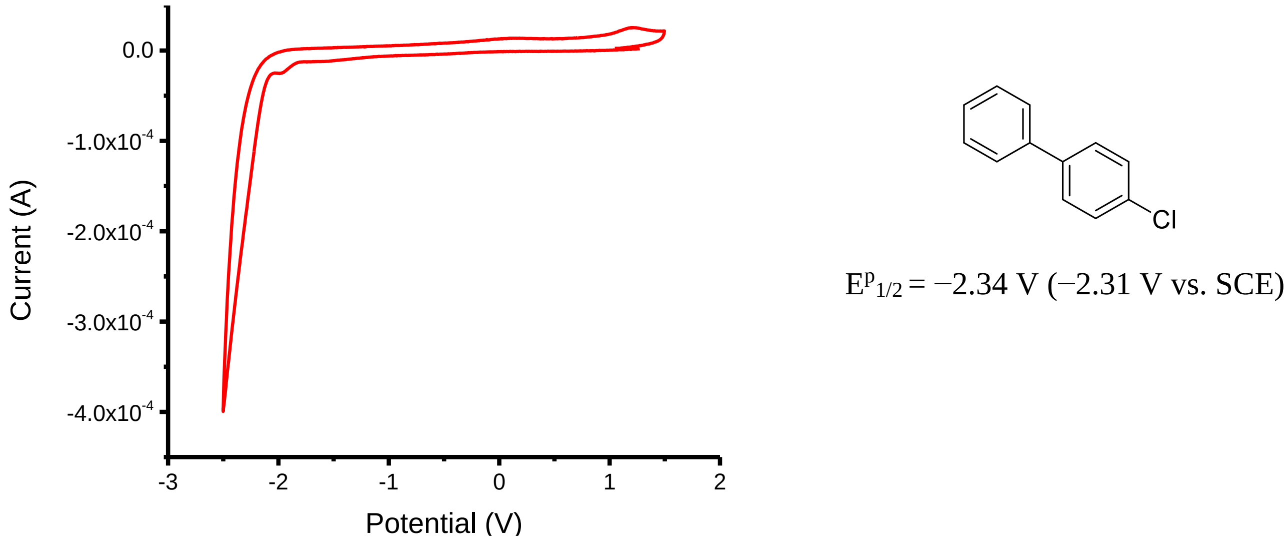

Figure S8. Cyclic voltammogram of 4-chlorobiphenyl using the IUPAC plotting convention. Starting point: −1.0 V, reductive scan.

G. Absorption and Emission Spectra

G1. UV-Vis Studies

All 1,4-DHP solutions were transferred to 3.5 mL macro fluorescence cuvettes and scanned from the 300–700 nm region using a Shimadzu UV-2600 UV-vis spectrometer.

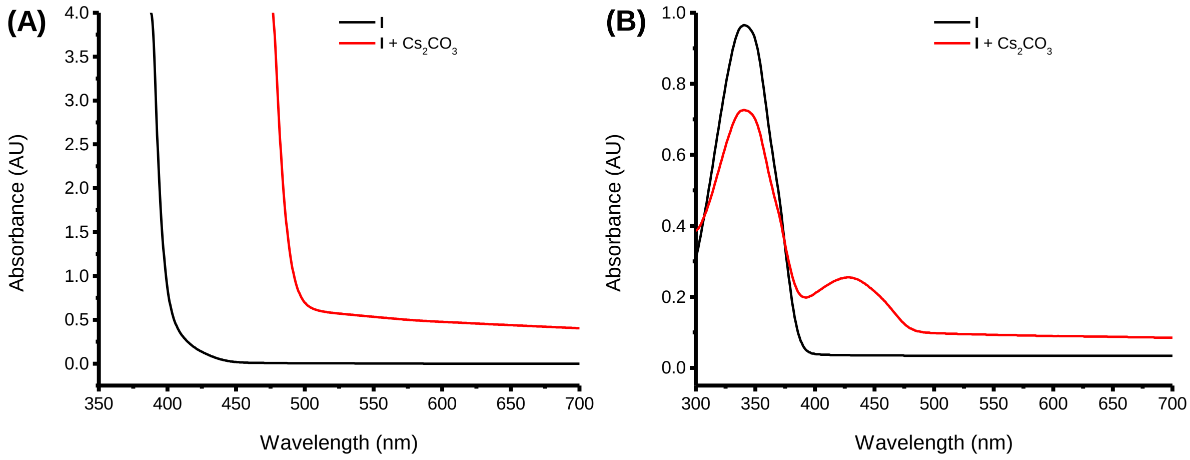

Figure S9. Absorption spectra of 1,4- DHP **I** and **I** + Cs_2CO_3. **(A)** Typical reaction concentration: [**I**] = 75 mM, [Cs_2CO_3] = 250 mM in MeCN. **(B)** [**I**] = 125 μM, [Cs_2CO_3] = 0.625 μM.

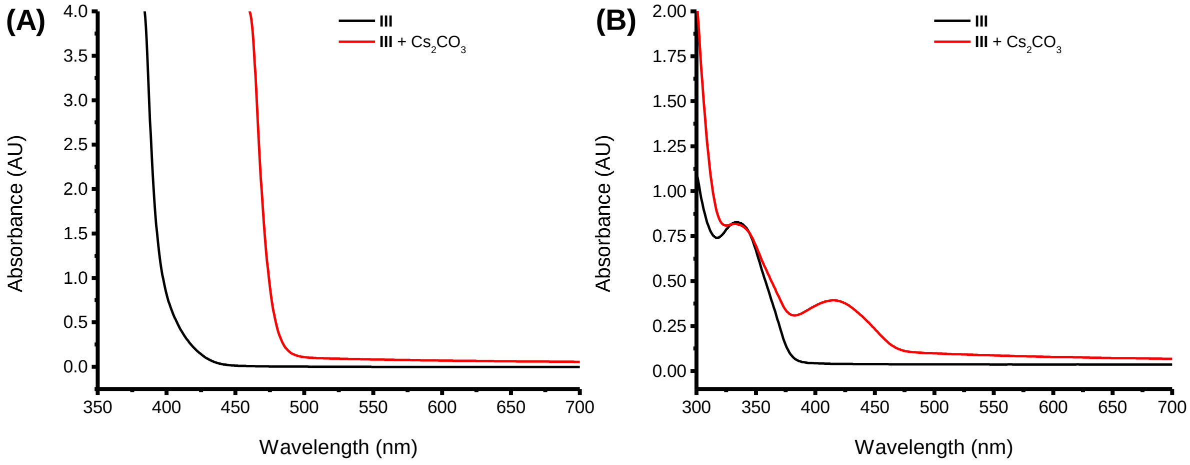

Figure S10. Absorption spectra of 1,4-DHP **III** and **III** + Cs_2CO_3. **(A)** Typical reaction concentration: [**III**] = 75 mM, [Cs_2CO_3] = 250 mM in MeCN. **(B)** [**III**] = 125 μM, [Cs_2CO_3] = 0.625 μM.

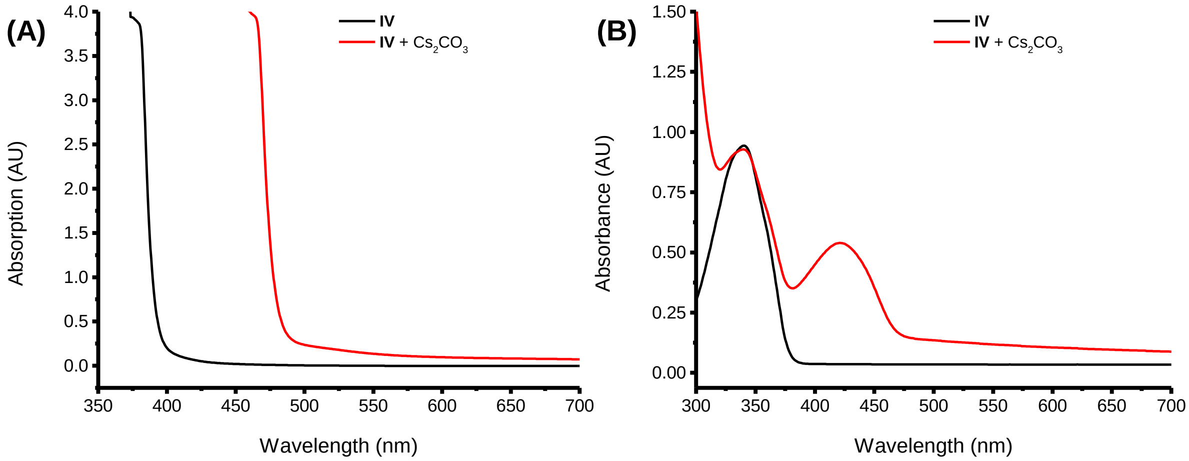

Figure S11. Absorption spectra of 1,4-DHP **IV** and **IV** + Cs_2CO_3. **(A)** Typical reaction concentration: [**IV**] = 75 mM, [Cs_2CO_3] = 250 mM in MeCN. **(B)** [**IV**] = 125 μM, [Cs_2CO_3] = 0.625 μM.

G2. EDA Complexation Studies

To rationalize the difference in reactivity observed for DHPs **I** and **IV** for the photodetosylation of *N*-tosyl aniline (see Tables S4 and S5), we investigated the possibility of the formation of electron donor-acceptor (EDA) complexes between the respective DHP anions and *N*-tosyl aniline. The formation of an EDA complex can be ascribed to the appearance of a new absorption band upon the mixing of donor and acceptor compounds that is distinct from the absorption of the individual reagents. Furthermore, it has previously been reported that *N*-tosyl compounds can serve as acceptors in EDA complex formation.[7] To determine if EDA complexation was occurring between *N*-tosyl aniline and the 1,4-DHPs anions of **I** and **IV**, we performed a series of UV-vis studies. As shown in Figures S12 and S13, a new absorption band is observed upon mixing the individual reaction components, indicative of EDA complex formation. However, a similar degree of complexation with *N*-tosyl aniline was observed for 1,4-DHP anions **I** and **IV**. Therefore, the increased reactivity observed for 1,4-DHP **IV** in our photodetosylation reactions is likely not attributable to preferential formation of an EDA complex.

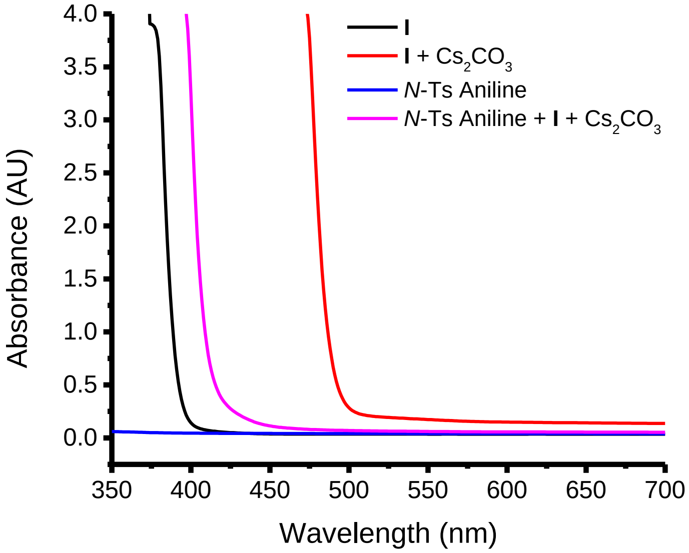

Figure S12. Absorption spectra of 1,4-DHP **I**, **I** + Cs_2CO_3, *N*–Ts aniline, and **I** + Cs_2CO_3 + *N*–Ts aniline. [**I**] = 75 mM, [Cs_2CO_3] = 100 mM, [*N*–Ts aniline] = 50 mM in MeCN.

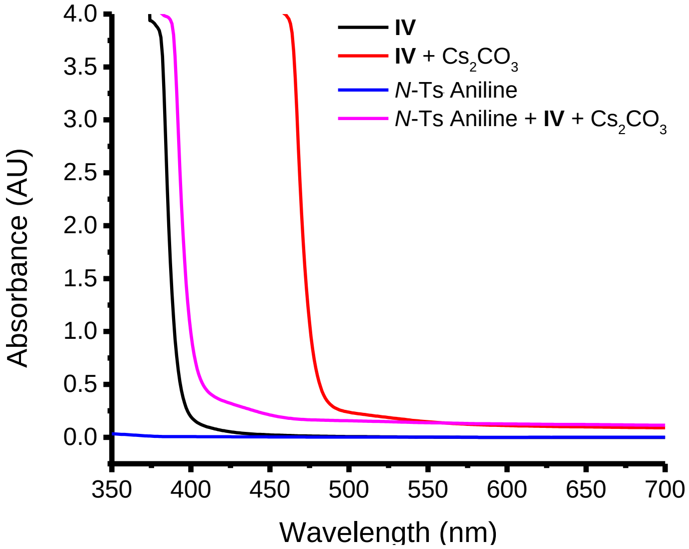

Figure S13. Absorption spectra of 1,4-DHP **IV**, **IV** + Cs_2CO_3, *N*–Ts aniline, and **IV** + Cs_2CO_3 + *N*–Ts aniline. [**IV**] = 75 mM, [Cs_2CO_3] = 100 mM, [*N*–Ts aniline] = 50 mM in MeCN.

G3. Fluorescence Studies

All 1,4-DHP solutions were transferred to 3.5 mL macro fluorescence cuvettes and emission spectra were recorded on a Fluorolog3 fluorescence spectrometer. Solutions of 1,4-DHP anions were prepared by adding 1.1 equiv of KO^{t}Bu.

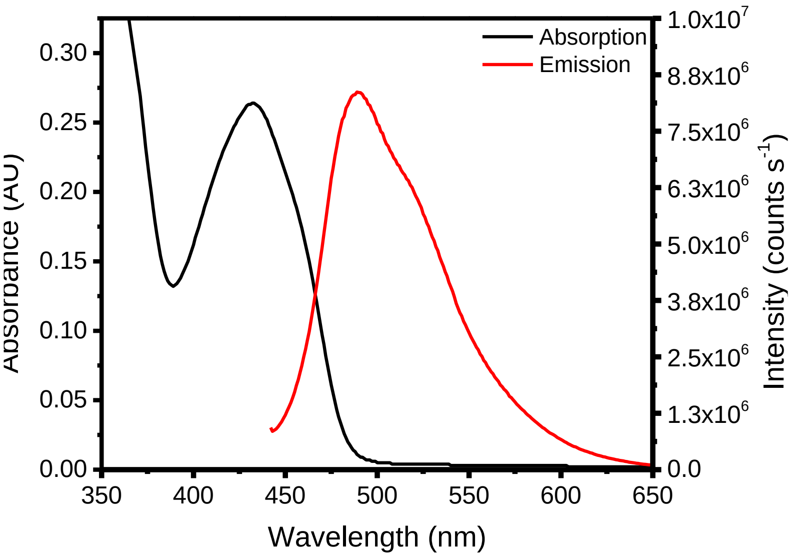

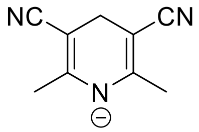

$E_{0,0}$ = 466 nm = 61.4 kcal/mol
= 2.66 eV

Figure S14. Absorption spectrum and emission spectrum of 1,4-DHP **I** anion using an excitation wavelength of 432 nm. [**I**] = 0.25 mM, [KO^{t}Bu] = 0.275 mM in MeCN.

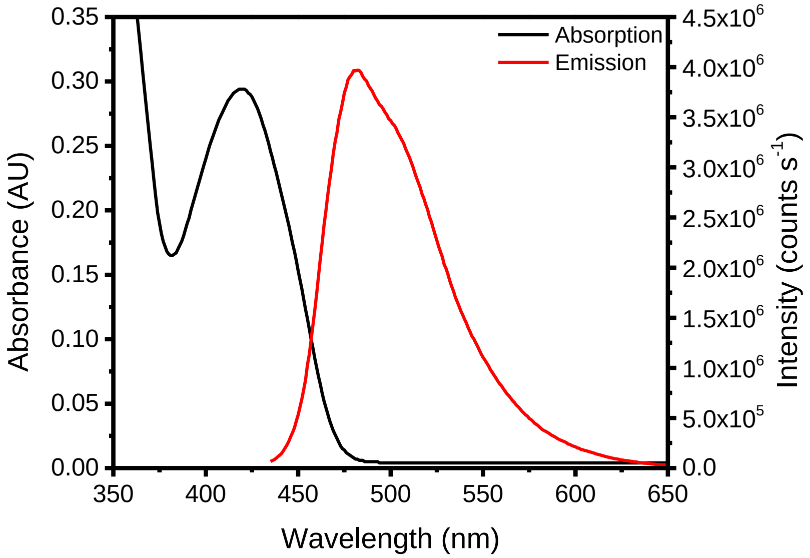

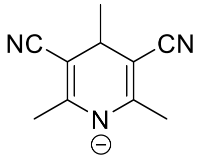

$E_{0,0}$ = 457 nm = 62.6 kcal/mol
= 2.71 eV

Figure S15. Absorption spectrum and emission spectrum of 1,4-DHP **III** anion using an excitation wavelength of 421 nm. [**III**] = 0.25 mM, [KO^{t}Bu] = 0.275 mM in MeCN.

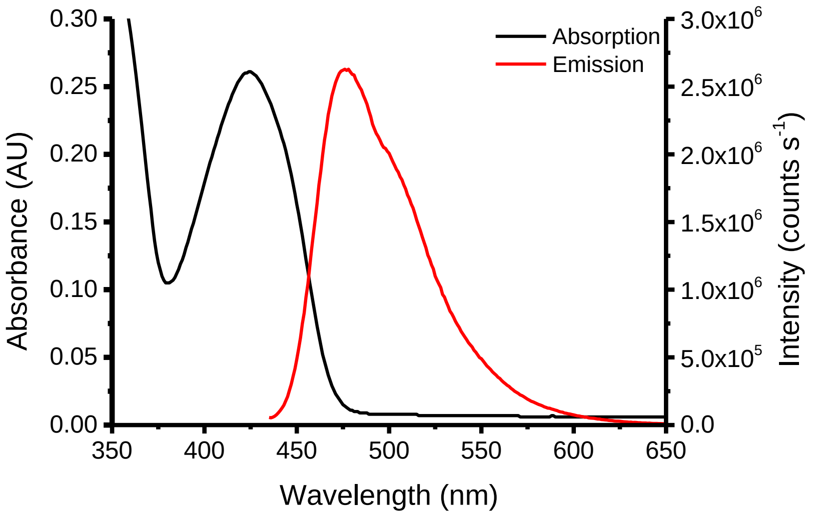

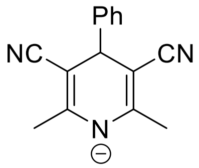

$\boldsymbol{E}_{\mathbf{0,0}}$ = 456 nm = 62.7 kcal/mol
= 2.72 eV

Figure S16. Absorption spectrum and emission spectrum of 1,4-DHP **IV** anion using an excitation wavelength of 424 nm. [**IV**] = 0.25 mM, [KO^{t}Bu] = 0.275 mM in MeCN.

H. References

1. Bin, C.; Deng-hui, W.; Li-zhu, W.; Li-ping, Z.; Zhen-he, T., Synthesis of 3,5-Dicyano-2,4,6-trimethylpyridine by photoinduced aromatization of 1,4-Dihydro-3,5-dicyano-2,4,6-trimethylpyridine. *Imaging Science and Photochemistry* **2007**, *25*, 161–164.
2. Luis, A. C.; Alfonso, G. O.; Rocio, M.; Monica, P.; Carmen, S. M., Synthesis of 2,3,4,7-tetrahydro[1,4]thiazepines from thiazolidines and β-enaminonitriles. *Tetrahedron*, **2008**, *64*, 3691–3700.
3. MacKenzie, I. A.; Wang, L.; Onuska, N. P. R; Williams, O. F.; Begam, K.; Moran, A. M.; Dunietz, B. D.; Nicewicz, D. A. Discovery and Characterization of an Acridine Radical Photoreductant. *Nature* **2020,** *580*, 76–80.
4. Chakrabarti, A.; Biswas, G. K.; Chakraborty, D. P. Photo-Fries Rearrangements in *N*-Sulphonyl Carbazoles. *Tetrahedron* **1989,** *45*, 5059–5064.
5. Long, Y.; Zhang, F.; Huang, S.; Zheng, Y. Preparation of Pyridine Bicyclic Compounds for Treating Retroviral Integrase Mediated Disease. CN104003986, 2014.
6. Auberger, N.; Stanovych, A.; Thétiot-Laurent, S. Efficient Synthesis of Both Diastereomers of β, γ-Diamino Acids from Phenylalanine and Tryptophan. *Amino Acids* **2016**, *48*, 2237–2242.
7. Hamada, T.; Nishida, A.; Matsumoto, Y.; Yonemitsu, O. Photohydrolysis of Sulfonamides via Donor-acceptor Ion Pairs with Electron-Donating Aromatics and its Application to the Selective Detosylation of Lysine Peptides. *J. Am. Chem. Soc.* **1980**, *102*, 3978–3980.

I. NMR Spectra

Methyl benzoate (2): ^{1}H NMR (400 MHz, $CDCl_3$)

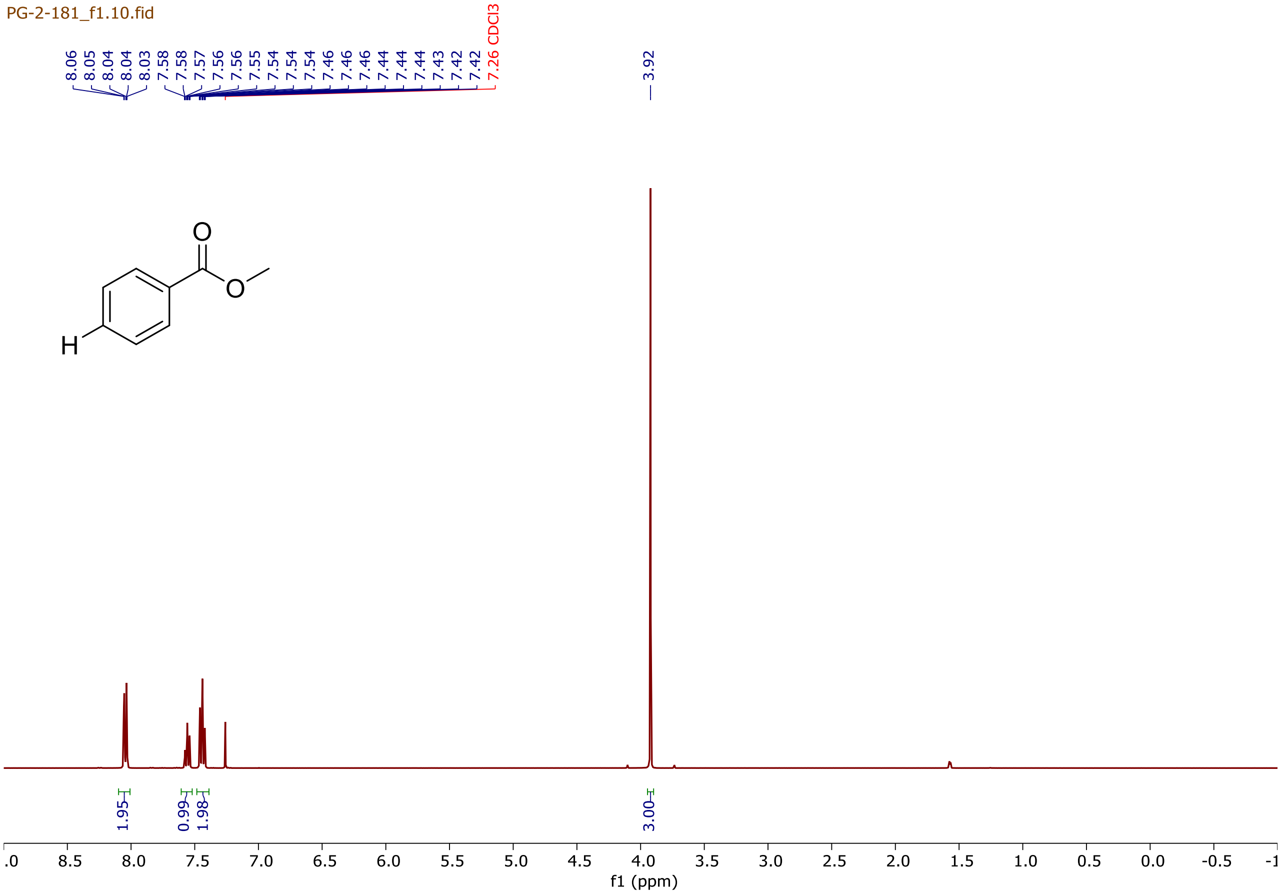

^{13}C NMR (101 MHz, $CDCl_3$)

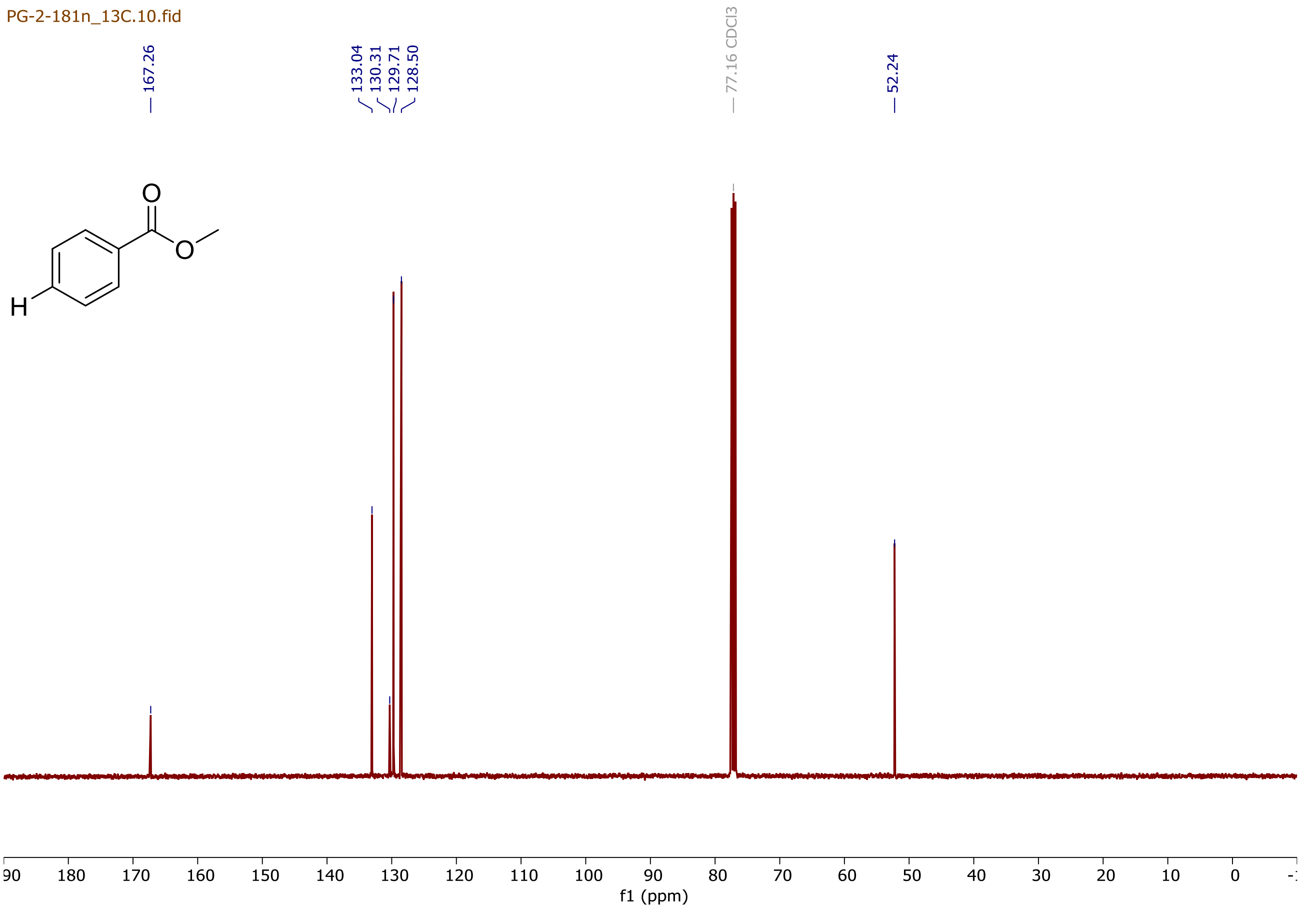

4-(4,4,5,5-Tetramethyl-1,3,2-dioxaborolan-2-yl)benzonitrile (3): ^{1}H NMR (400 MHz, $CDCl_3$)

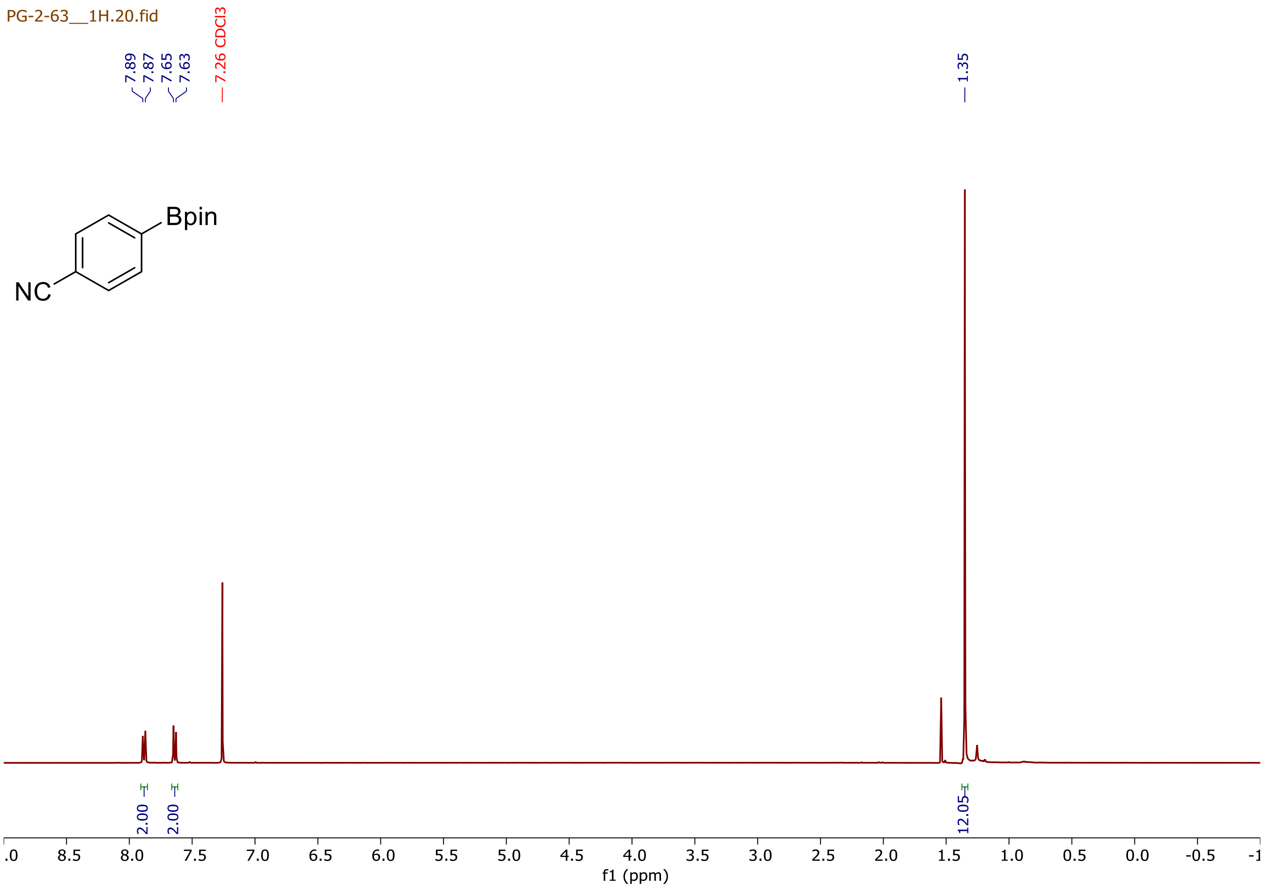

^{13}C NMR (101 MHz, $CDCl_3$)

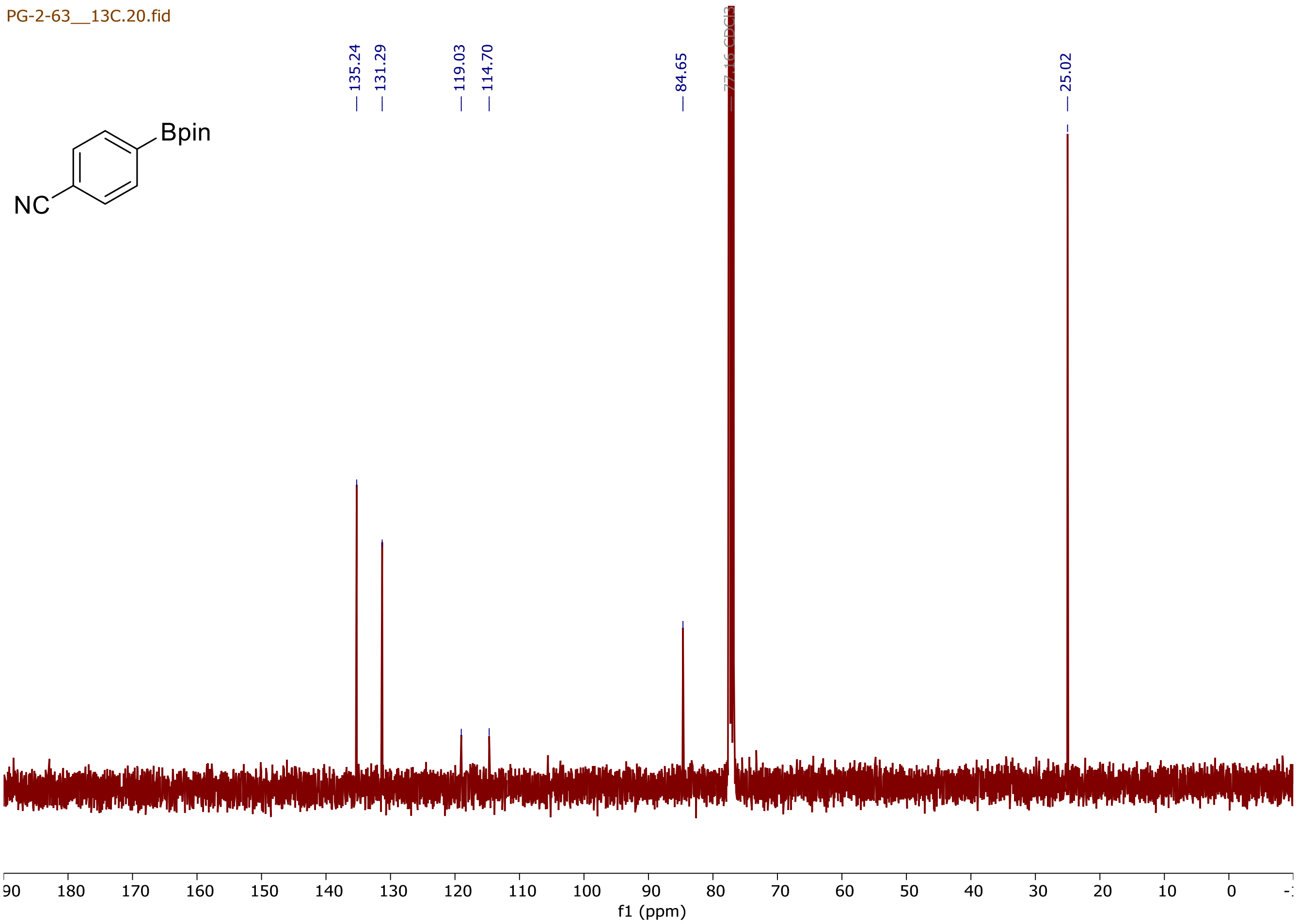

4,4,5,5-Tetramethyl-2-(4-(trifluoromethyl)phenyl)-1,3,2-dioxaborolane (4):
^{1}H NMR (400 MHz, $CDCl_3$)

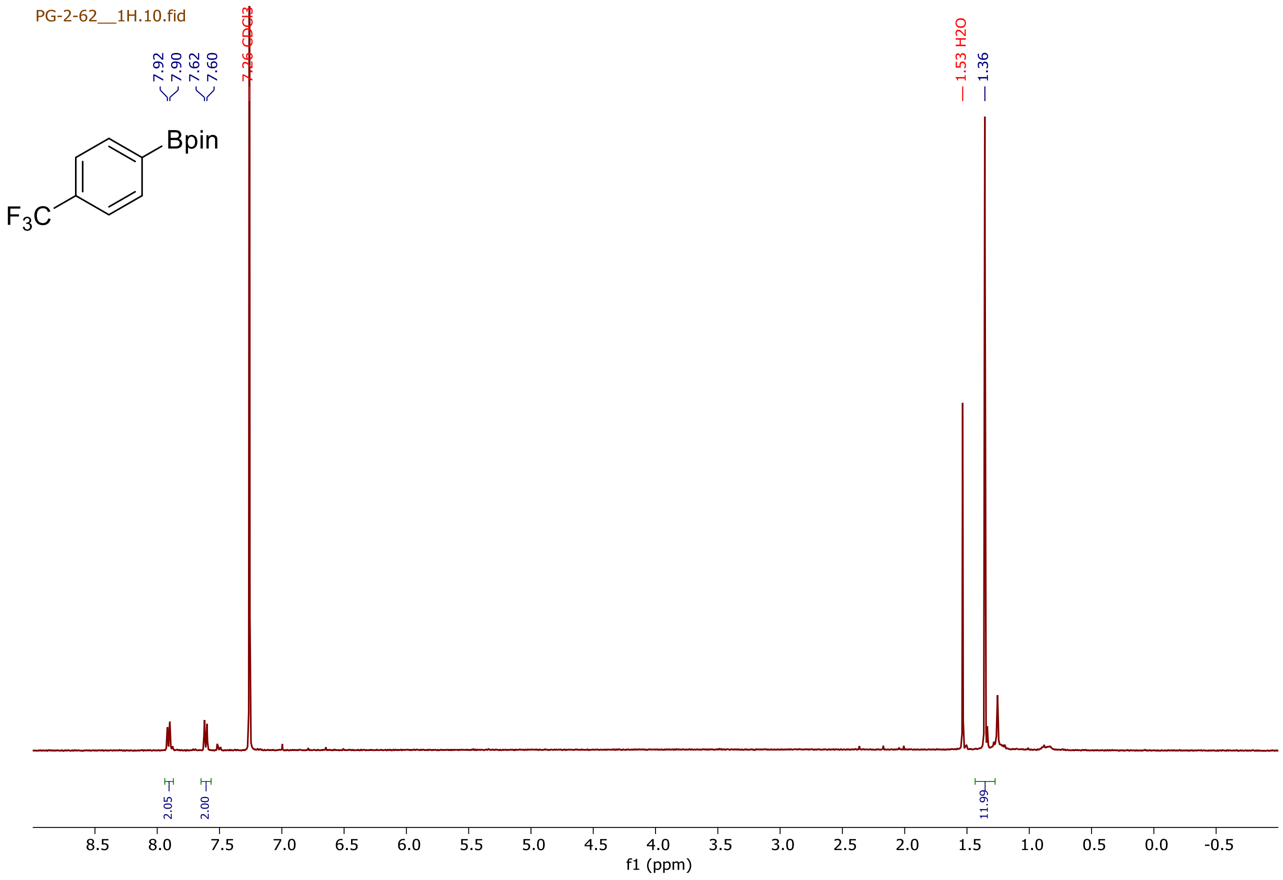

^{13}C NMR (101 MHz, $CDCl_3$)

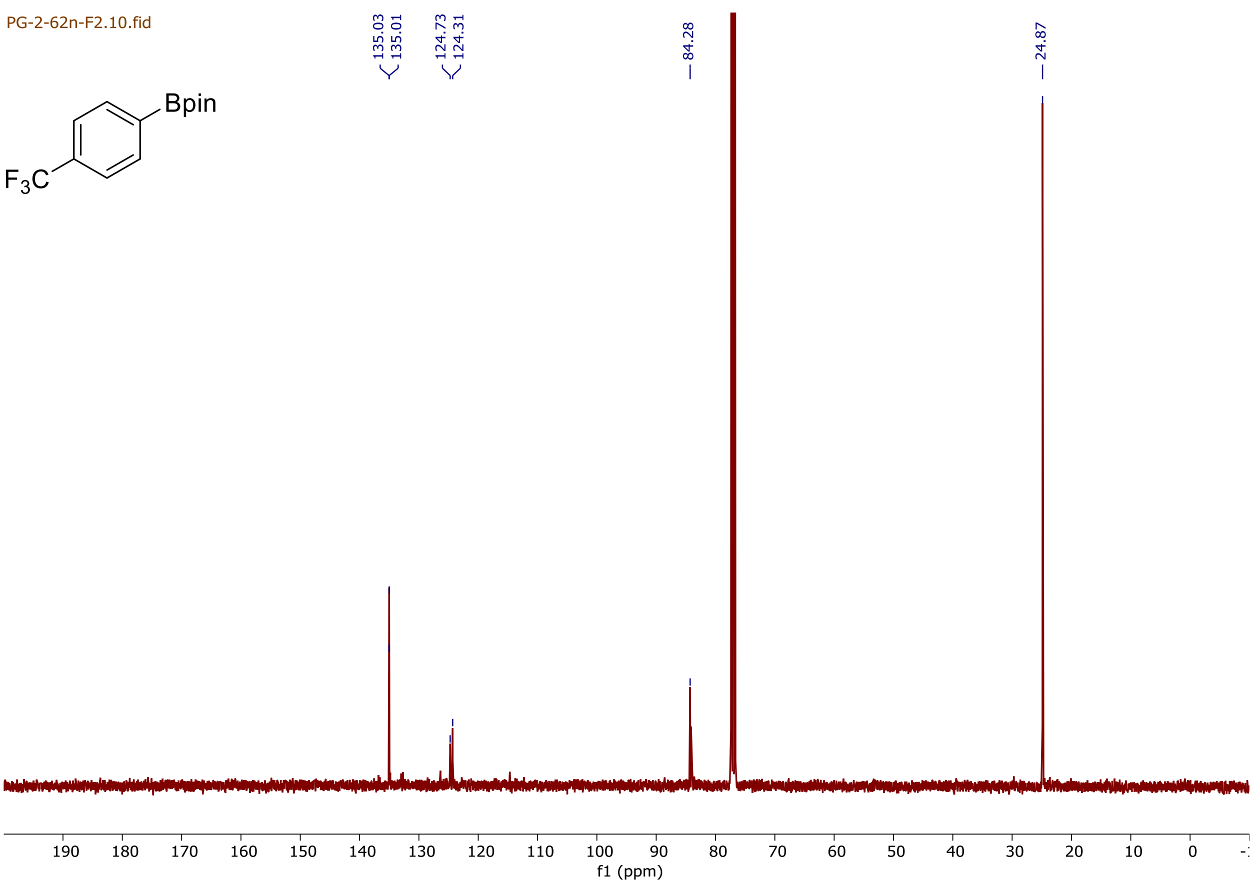

^{19}F NMR (376 MHz, $CDCl_3$)

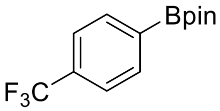

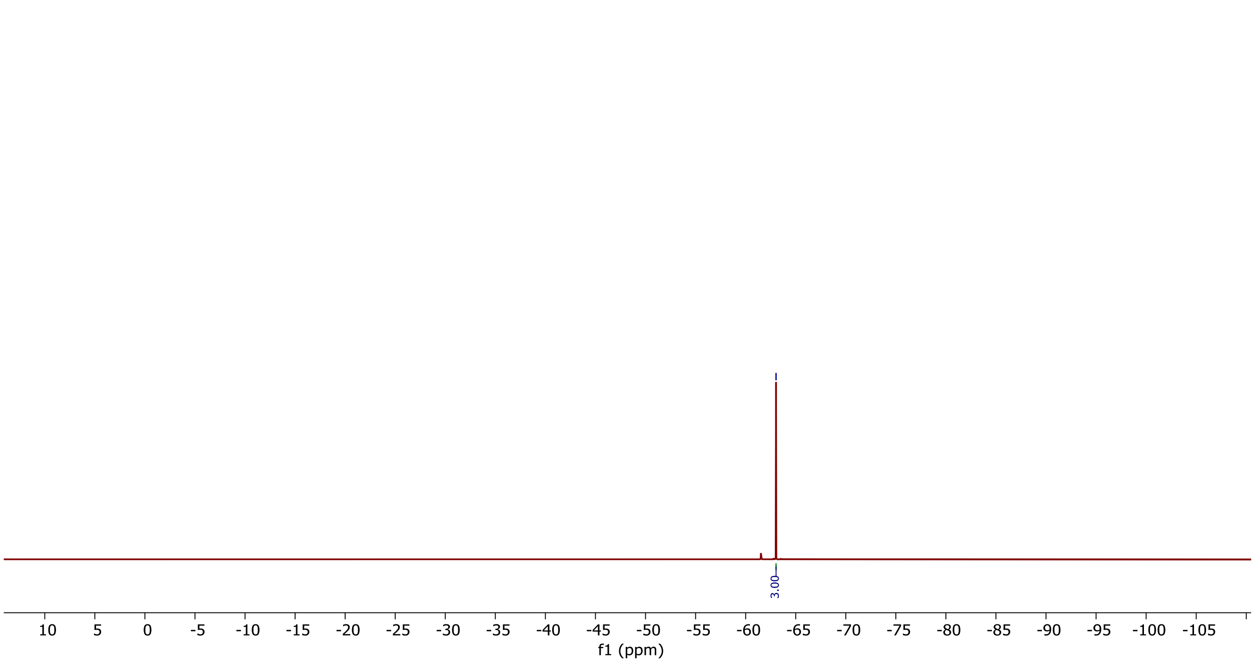

Methyl 4-(4,4,5,5-tetramethyl-1,3,2-dioxaboralan-2-yl)benzoate (5):
^{1}H NMR (400 MHz, $CDCl_3$)

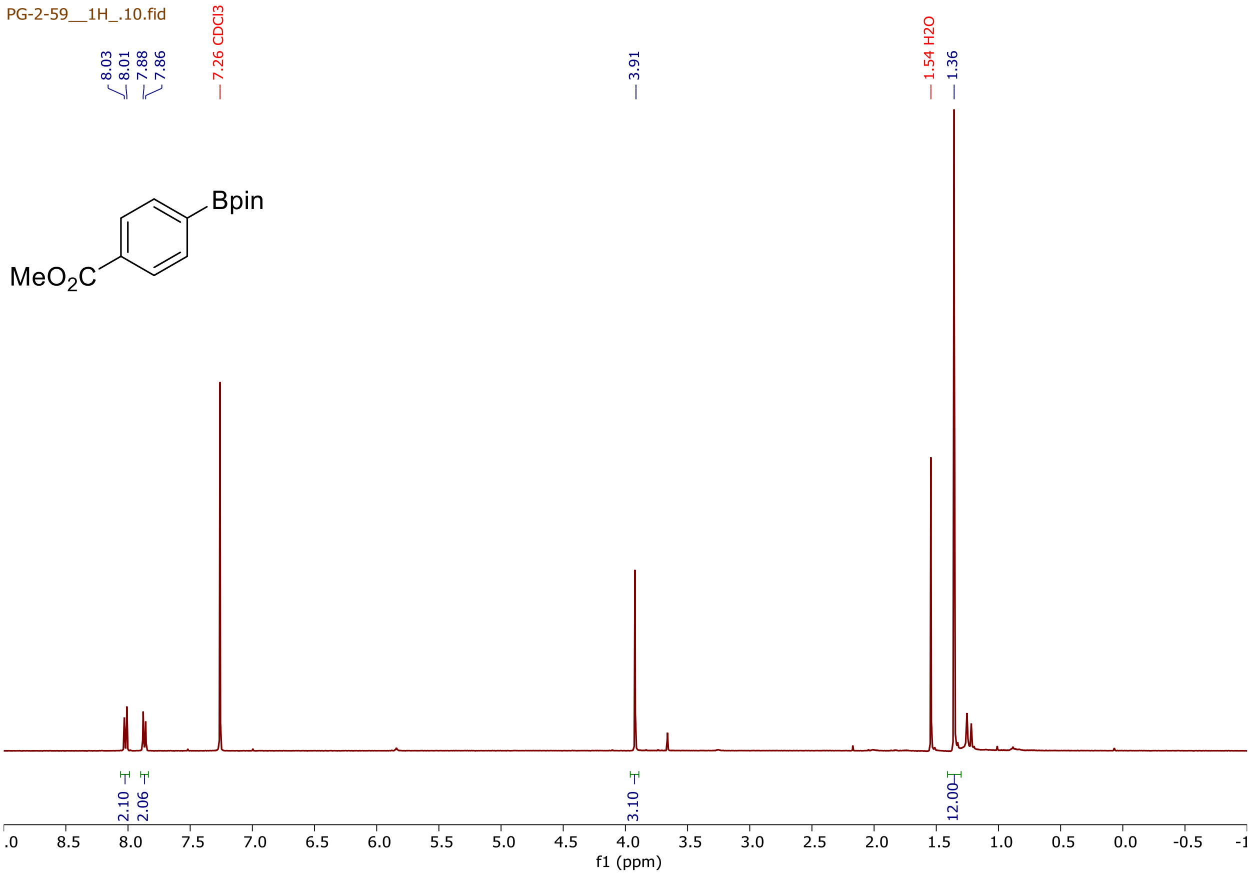

^{13}C NMR (101 MHz, $CDCl_3$)

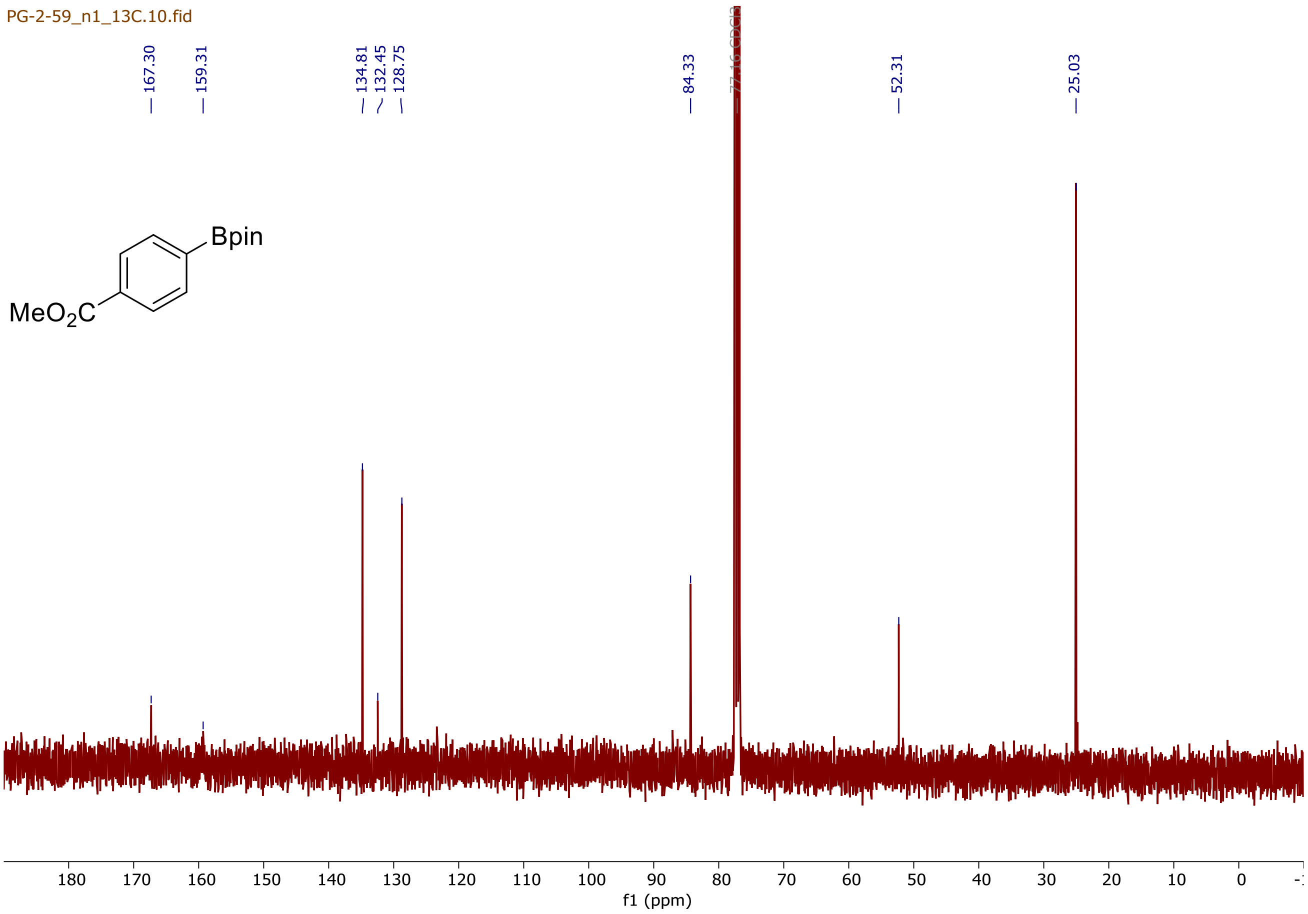

4,4,5,5-Tetramethyl-2-phenyl)-1,3,2-dioxaborolane (6): ^{1}H NMR (400 MHz, $CDCl_3$)

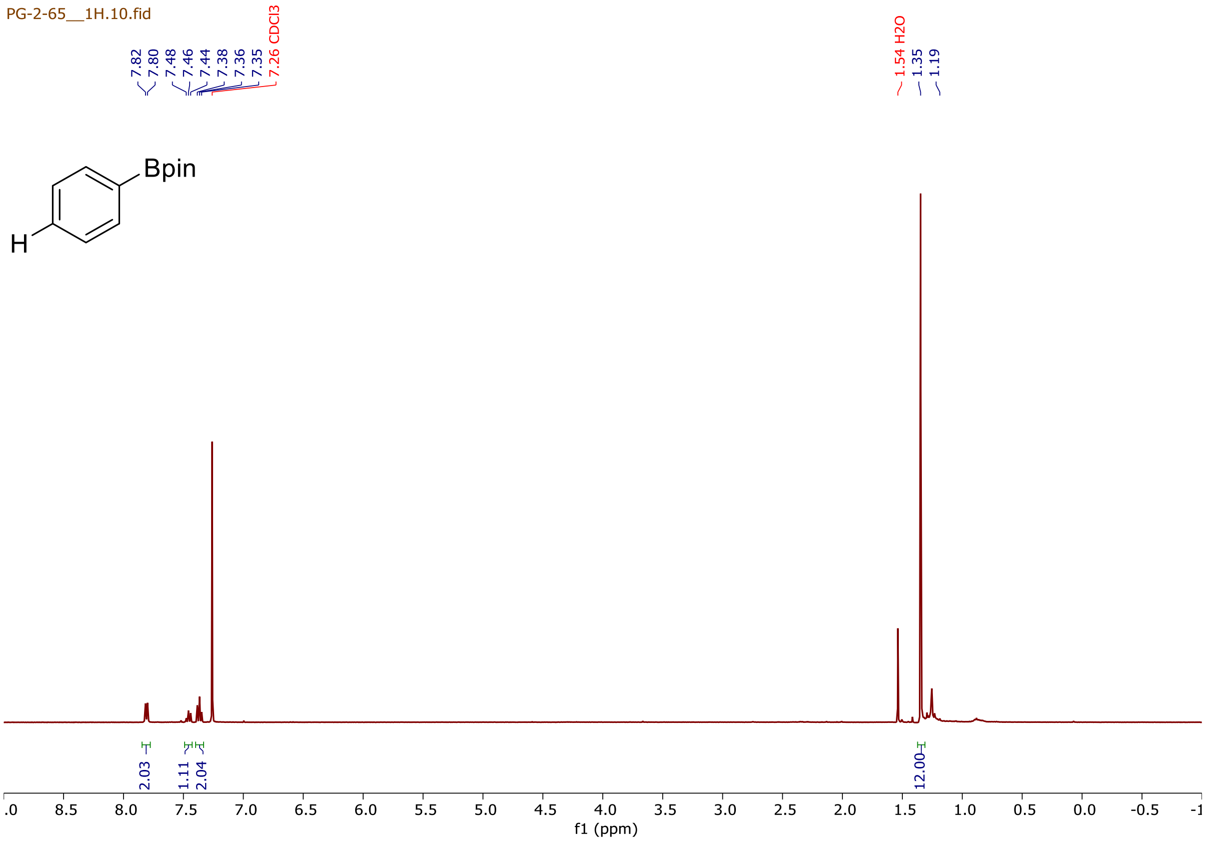

^{13}**C NMR** (101 MHz, $CDCl_3$)

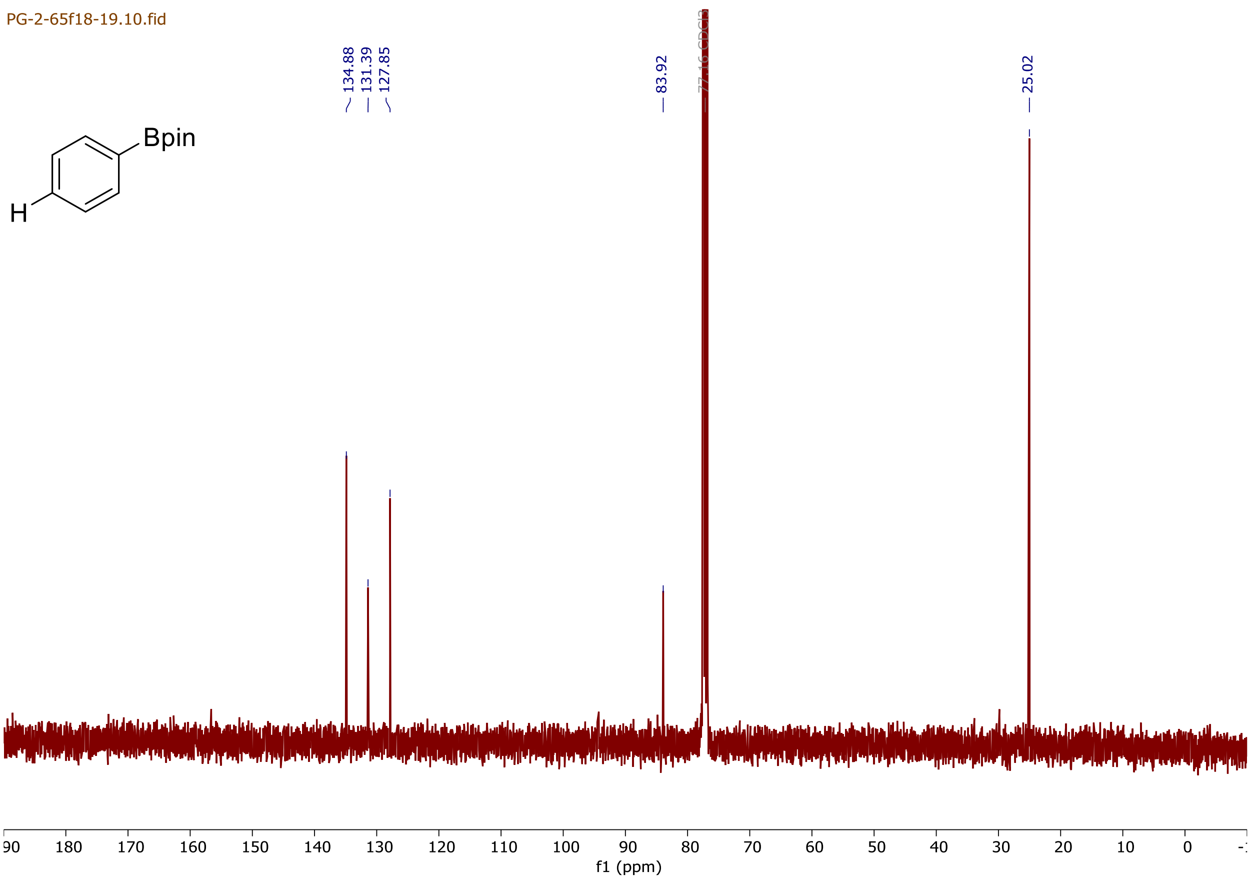

2-([1,1-Biphenyl]-4-yl)4,4,5,5-tetramethyl-1,3,2-dioxaborolane (7):
^{1}H NMR (400 MHz, $CDCl_3$)

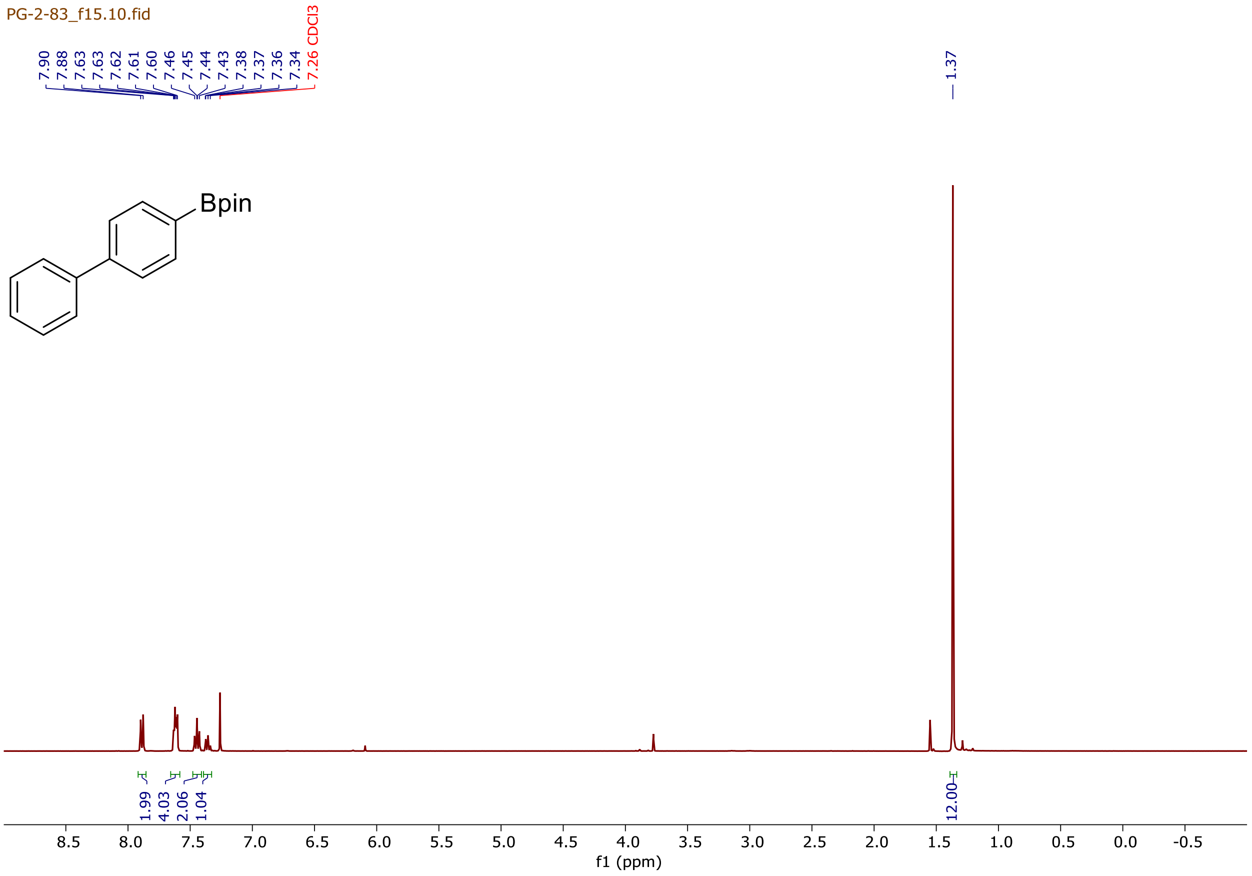

^{13}C NMR (101 MHz, $CDCl_3$)

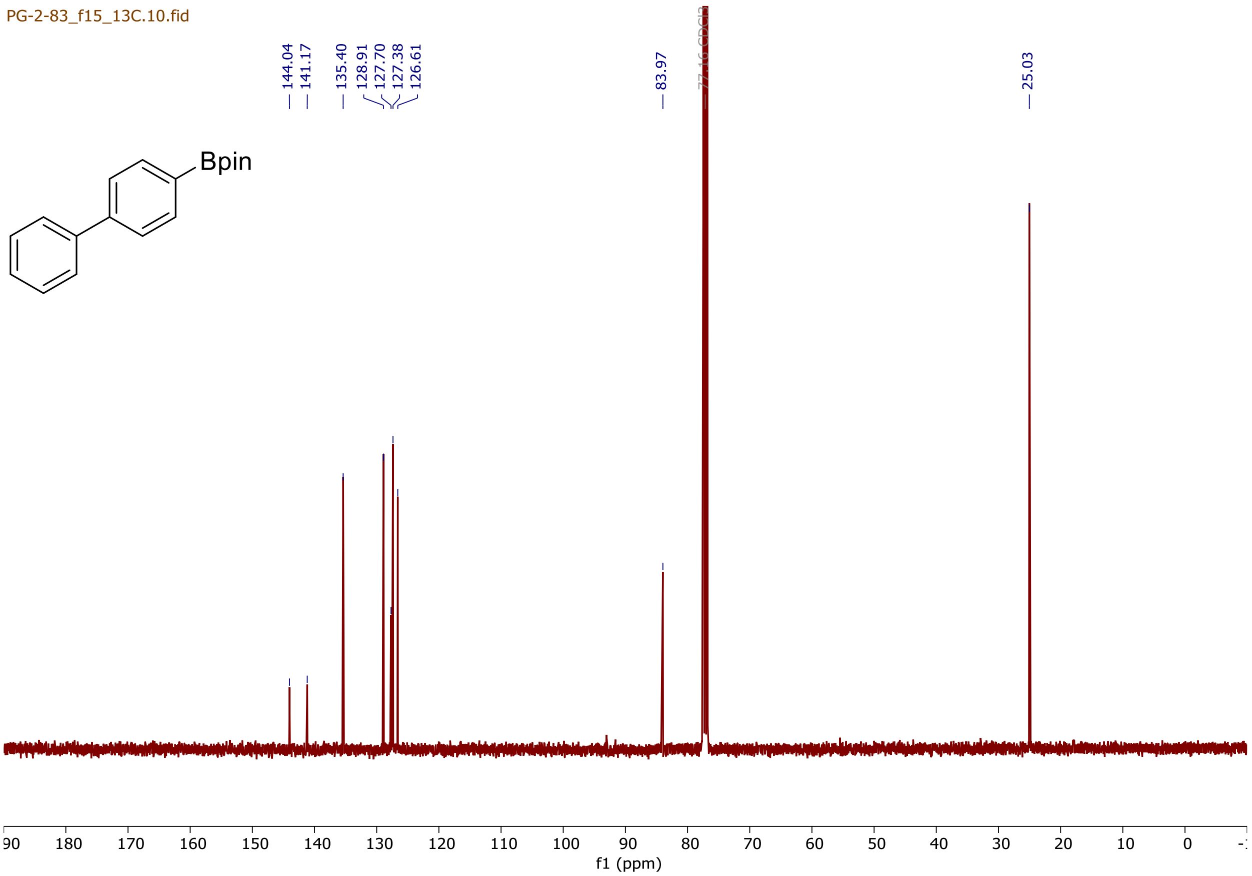

1*H*-indole (11): ^{1}H NMR (400 MHz, $CDCl_3$)

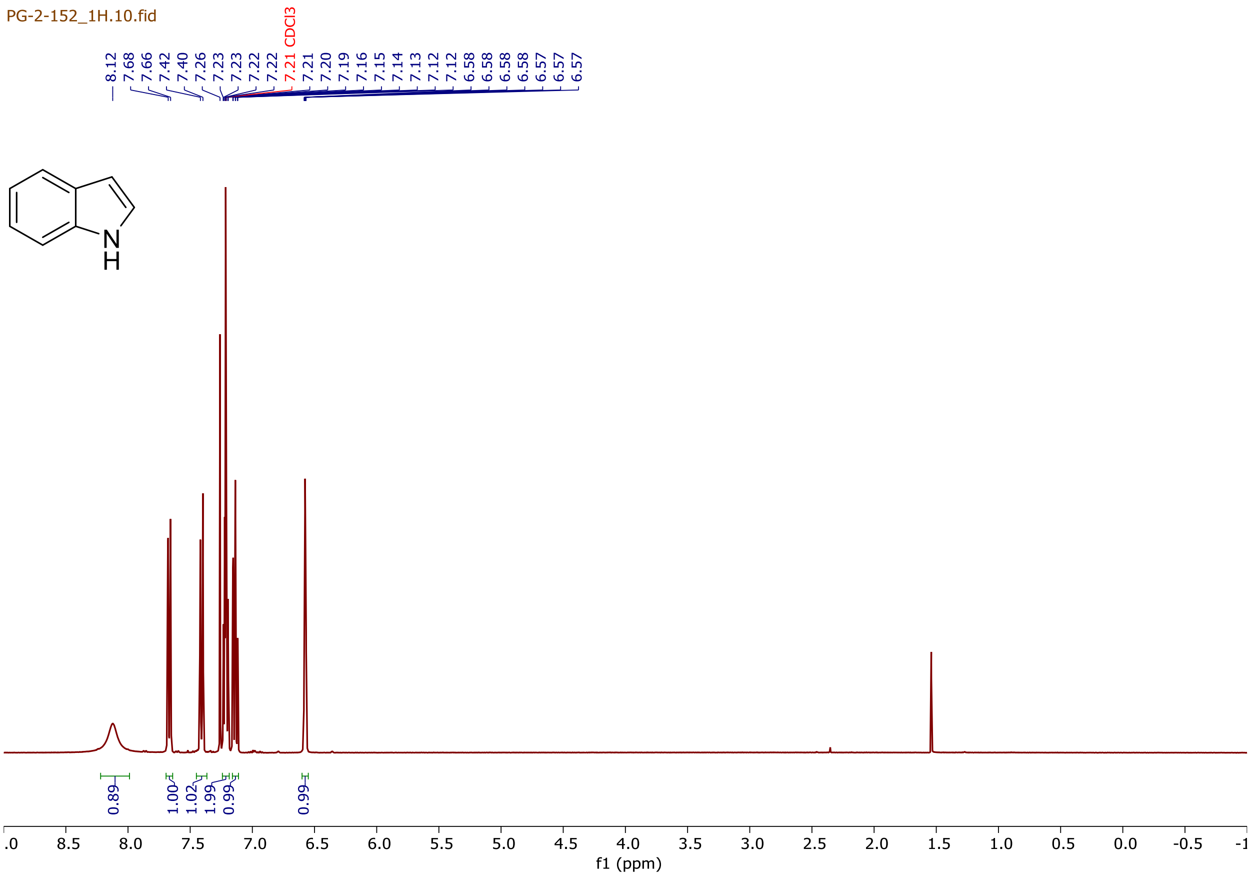

^{13}C NMR (101 MHz, $CDCl_3$)

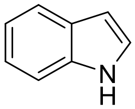

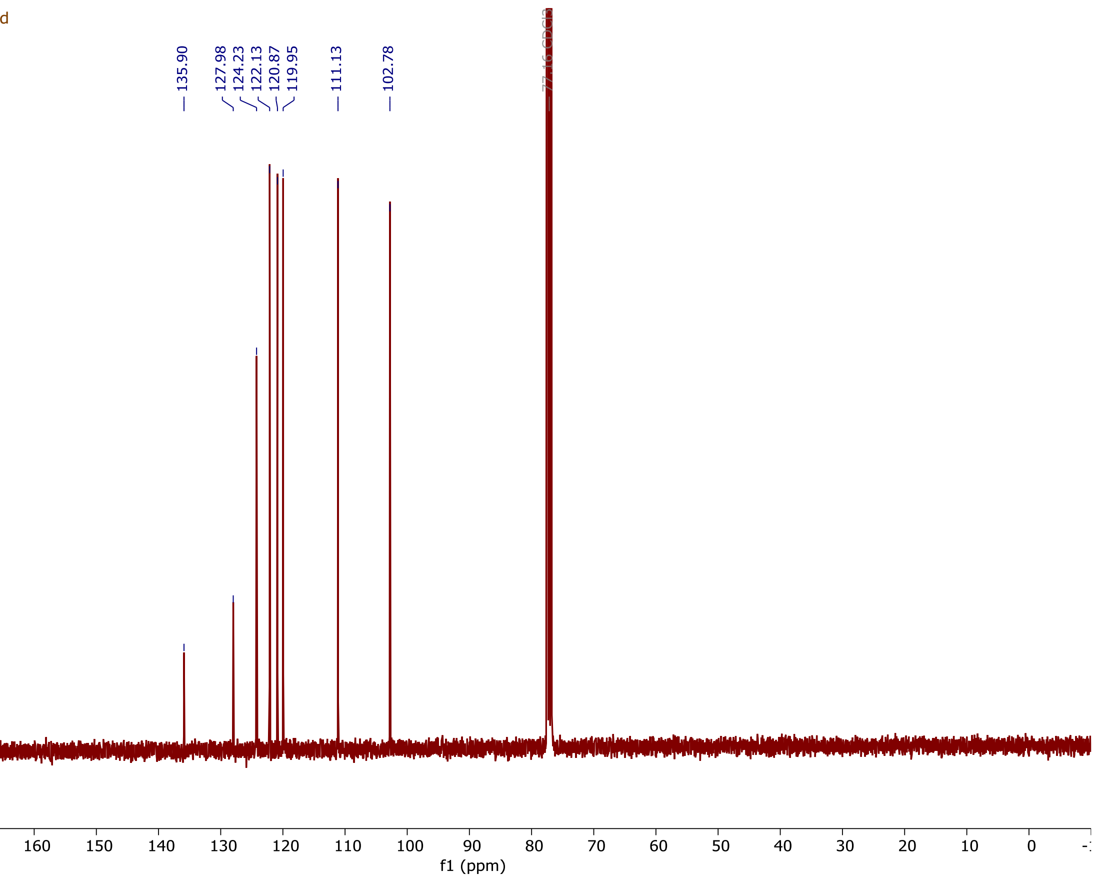

2,3-Dimethyl-1*H*-indole (12): ^{1}H NMR (400 MHz, $CDCl_3$)

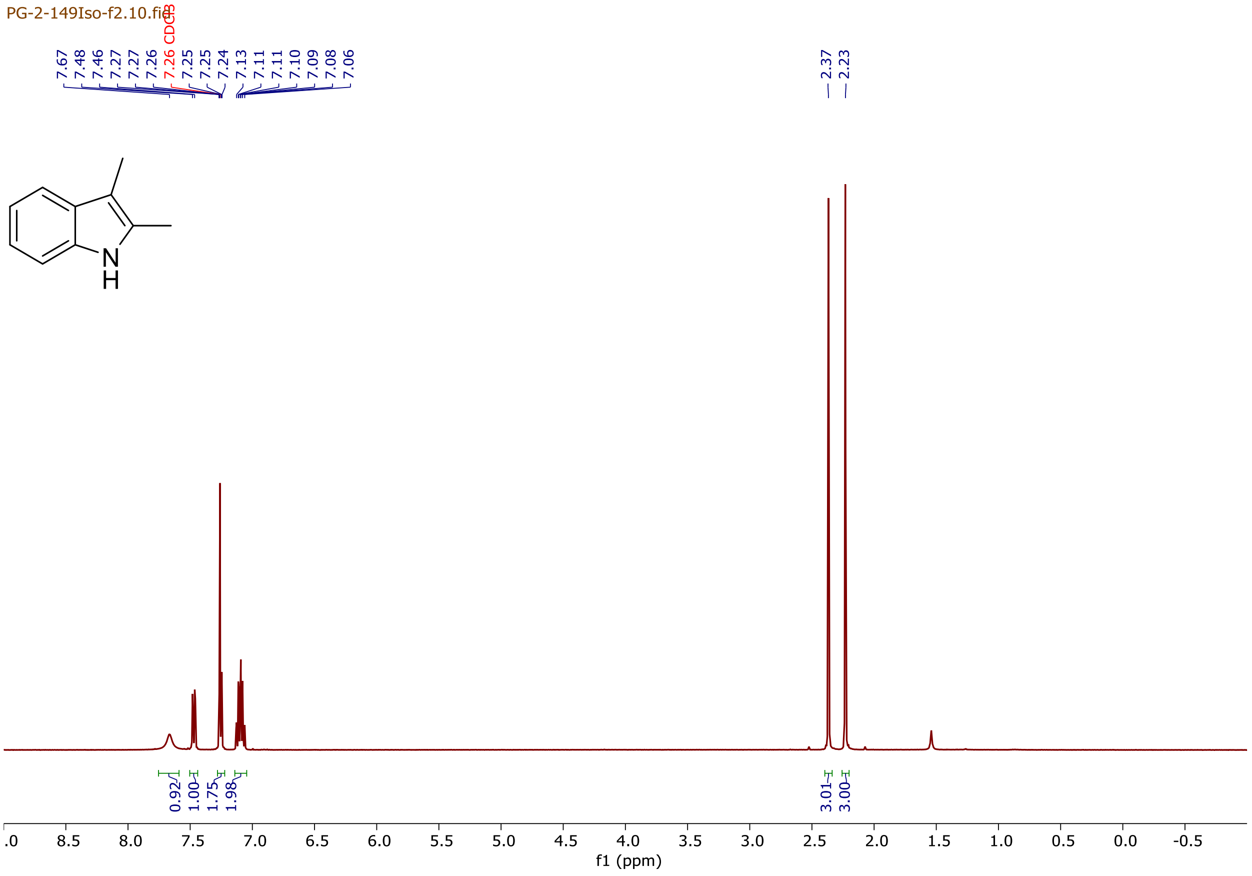

^{13}**C NMR** (101 MHz, $CDCl_3$)

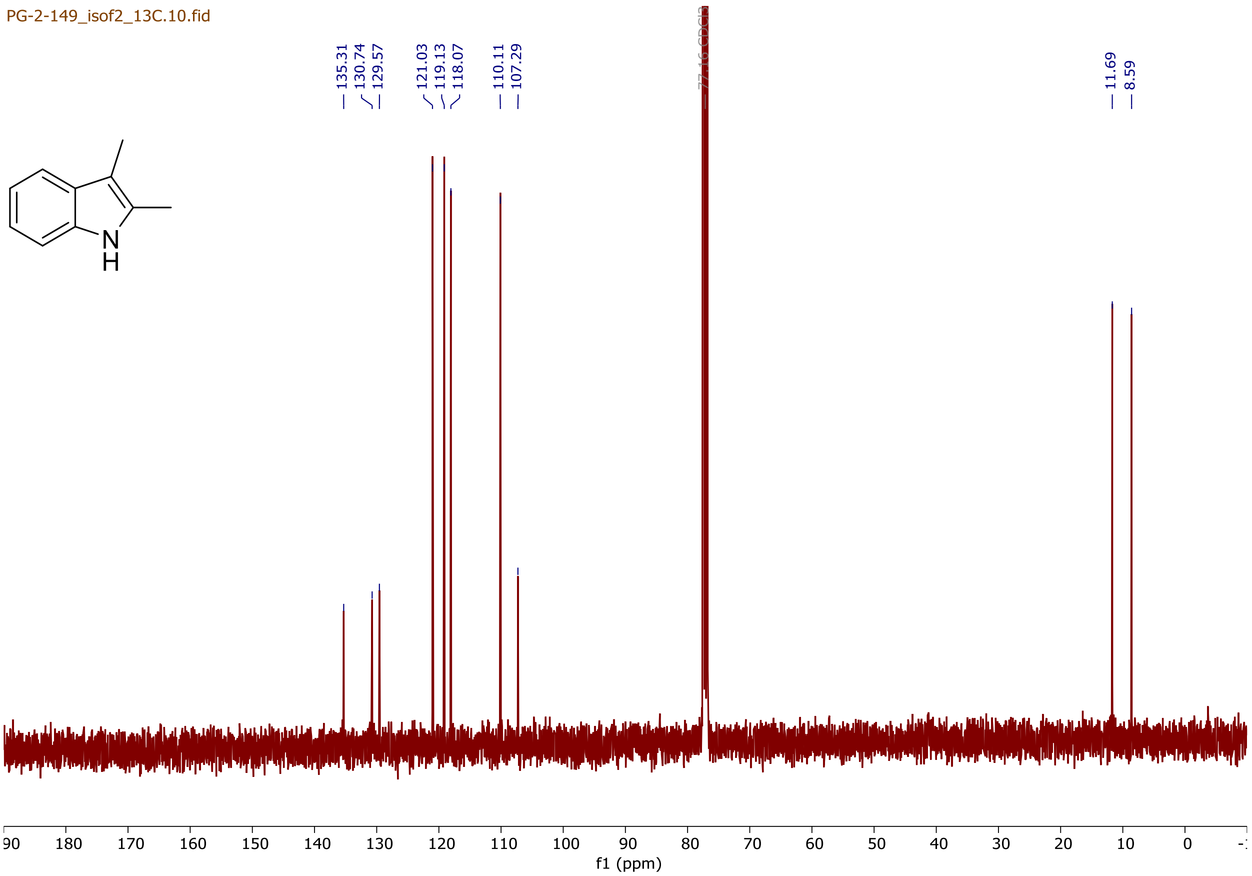

1*H*-Benzo[*d*]imidazole (13): ^{1}H NMR (400 MHz, DMSO-d_6)

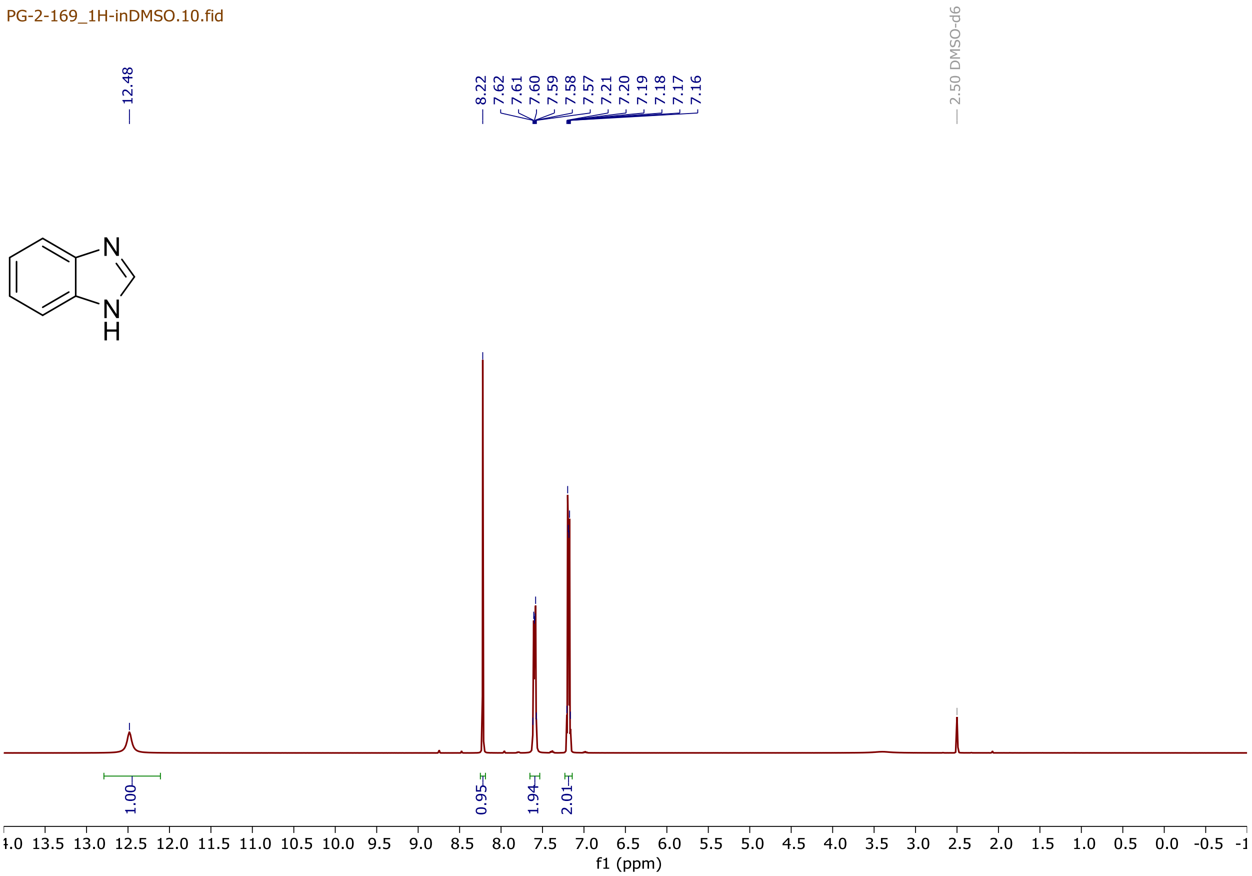

^{13}C NMR (101 MHz, DMSO-d$_6$)

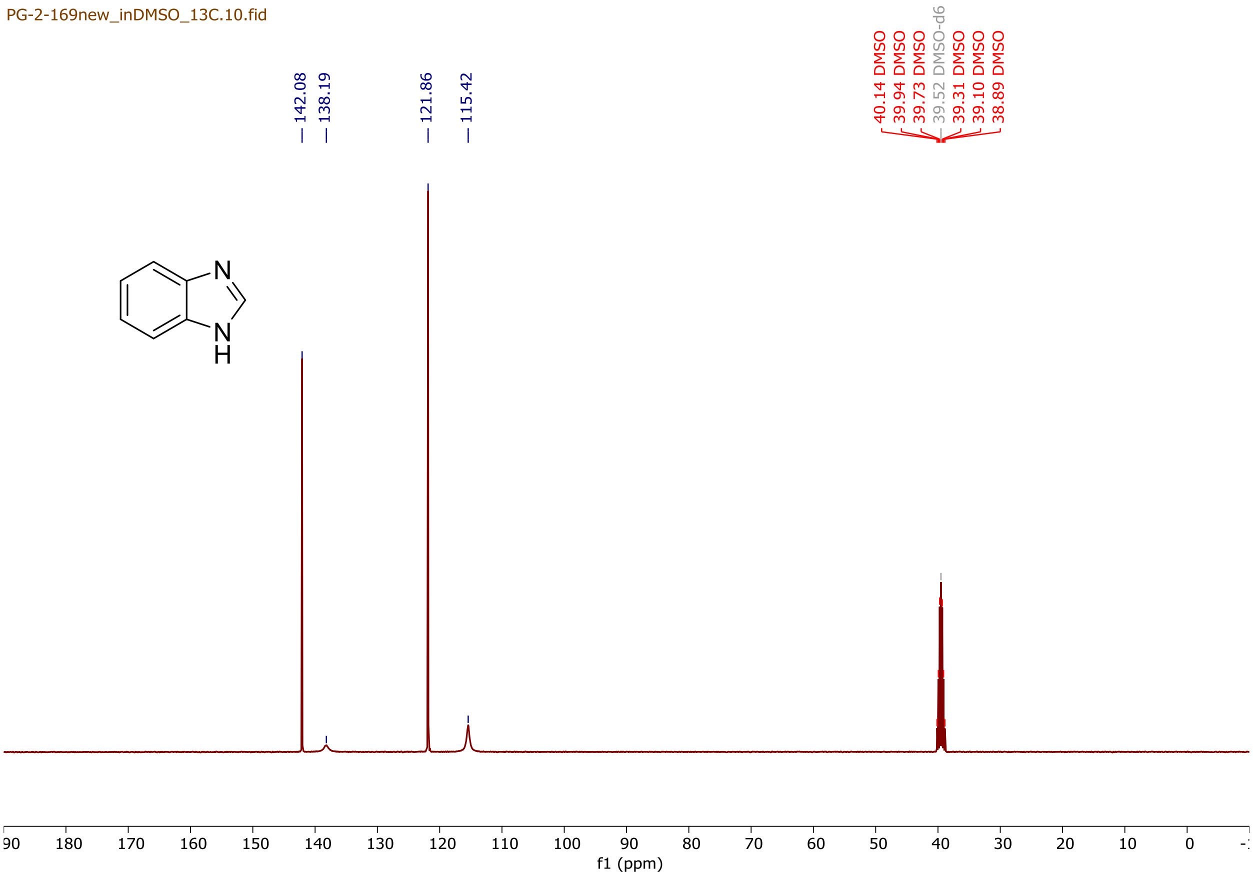

9*H*-Carbazole (14): ^{1}H NMR (400 MHz, $CDCl_3$)

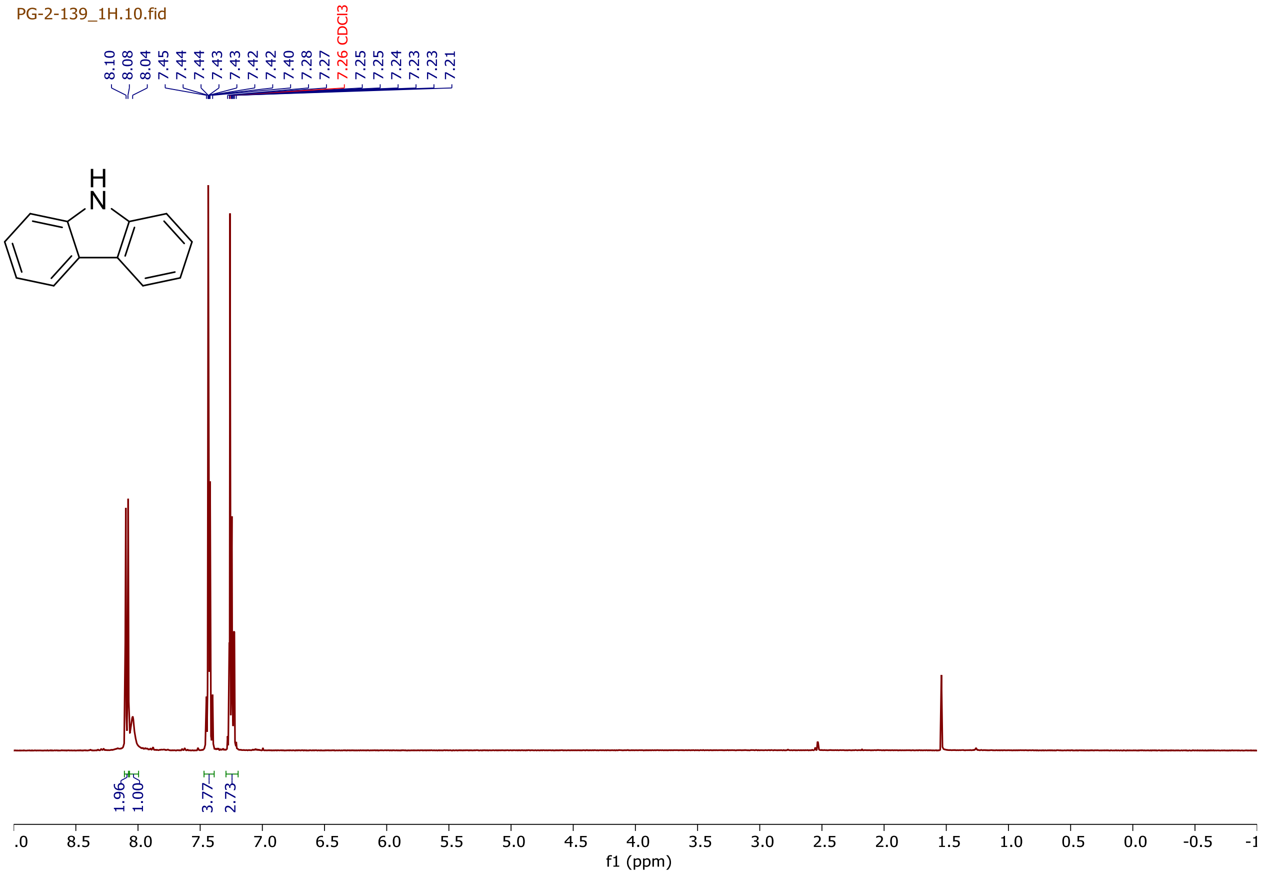

^{13}C NMR (101 MHz, $CDCl_3$)

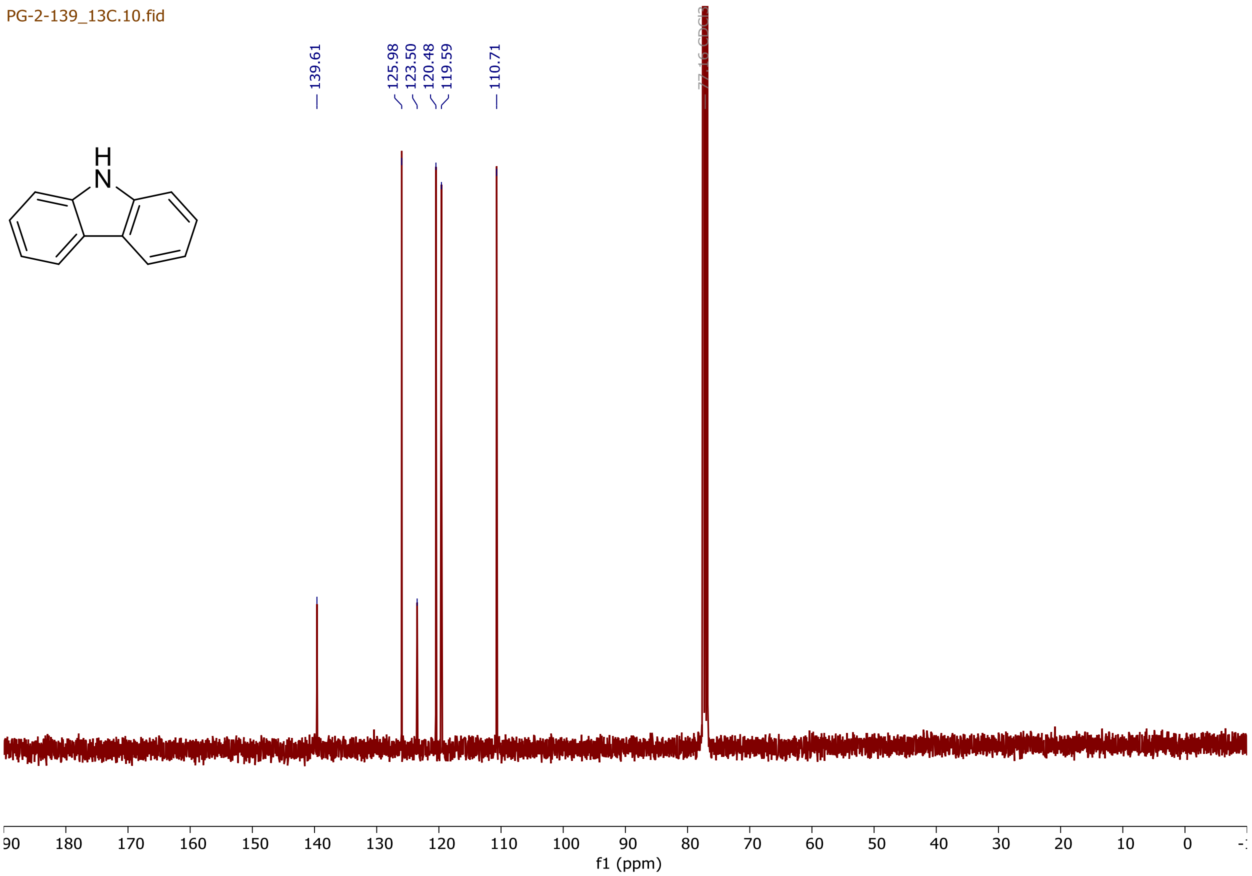

Diphenylamine (15): ^{1}H NMR (400 MHz, $CDCl_3$)

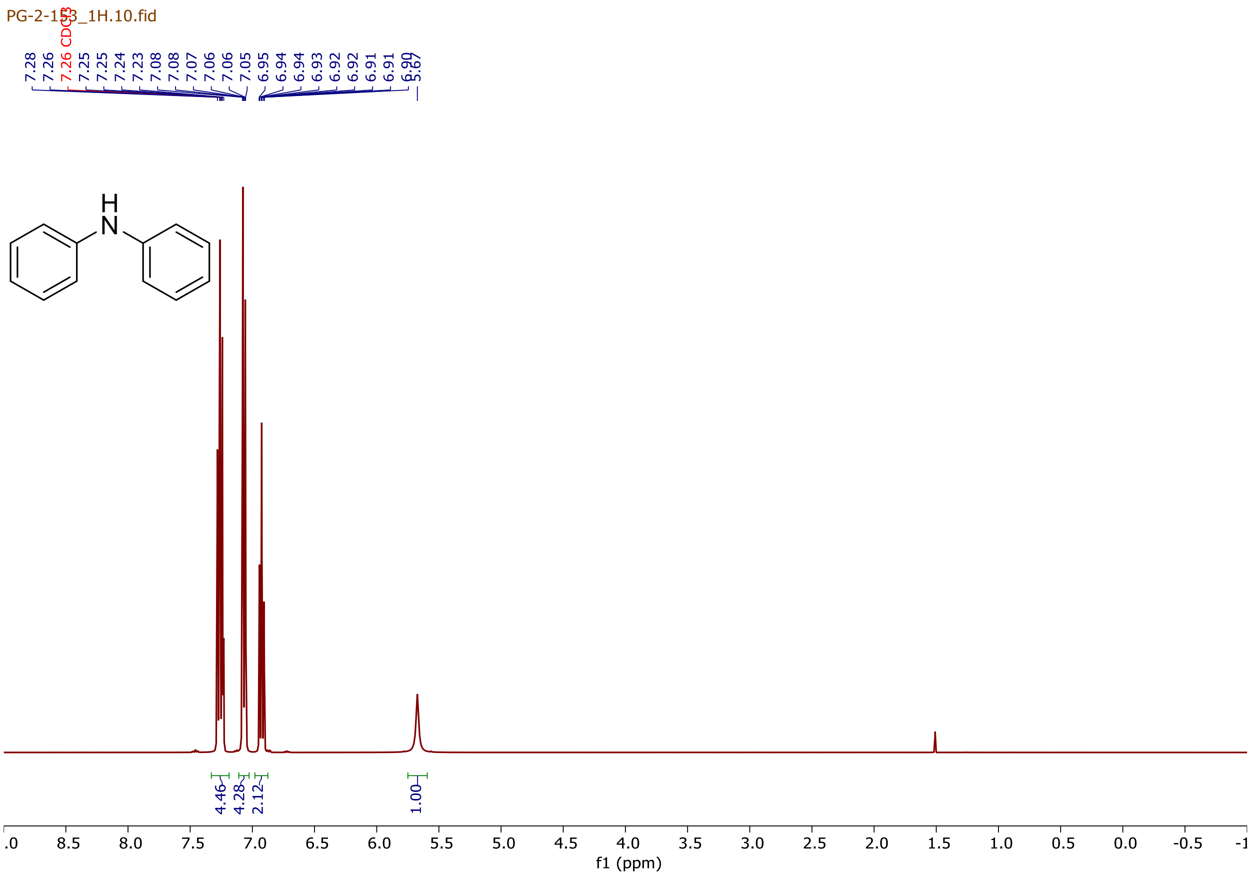

^{13}C NMR (101 MHz, $CDCl_3$)

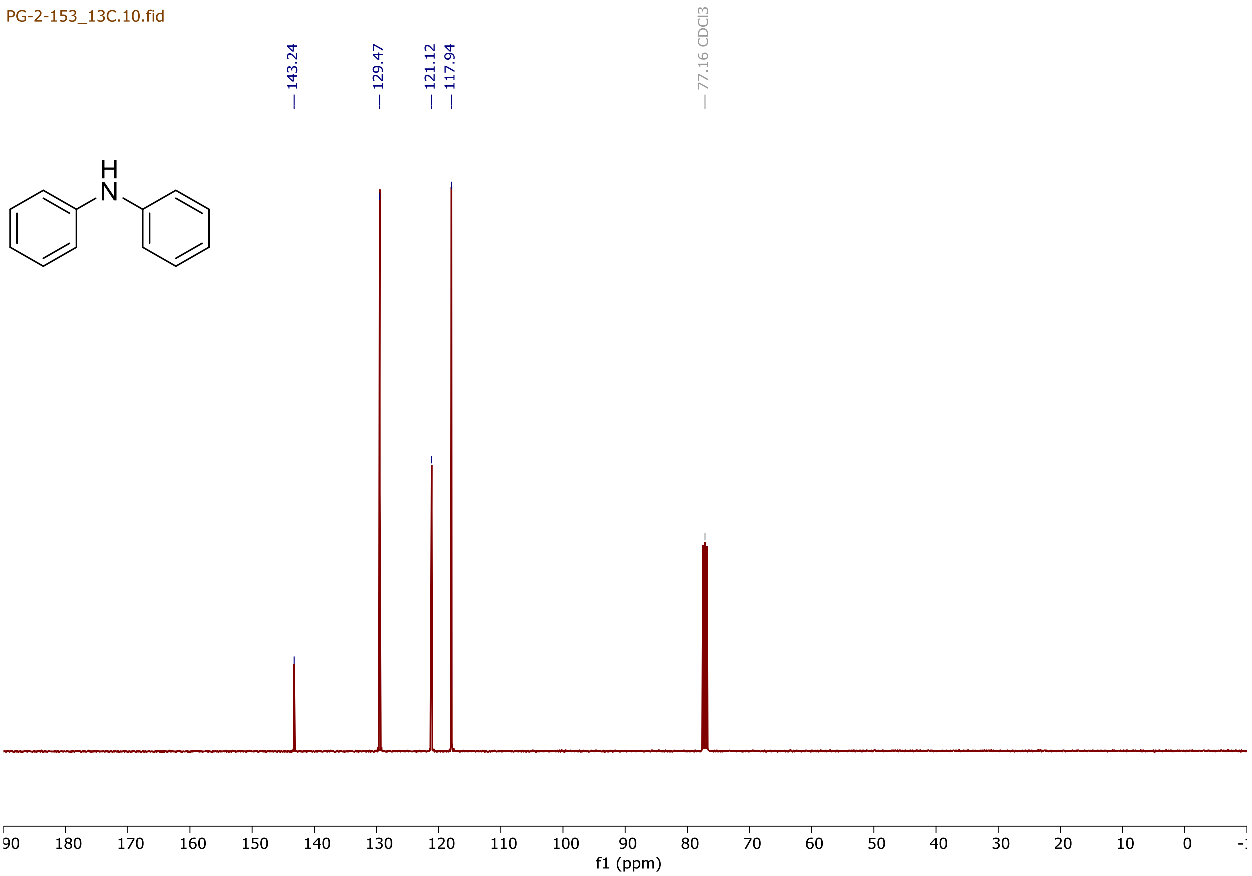

***N*-Benzylamine (16): ^{1}H NMR** (400 MHz, $CDCl_3$)

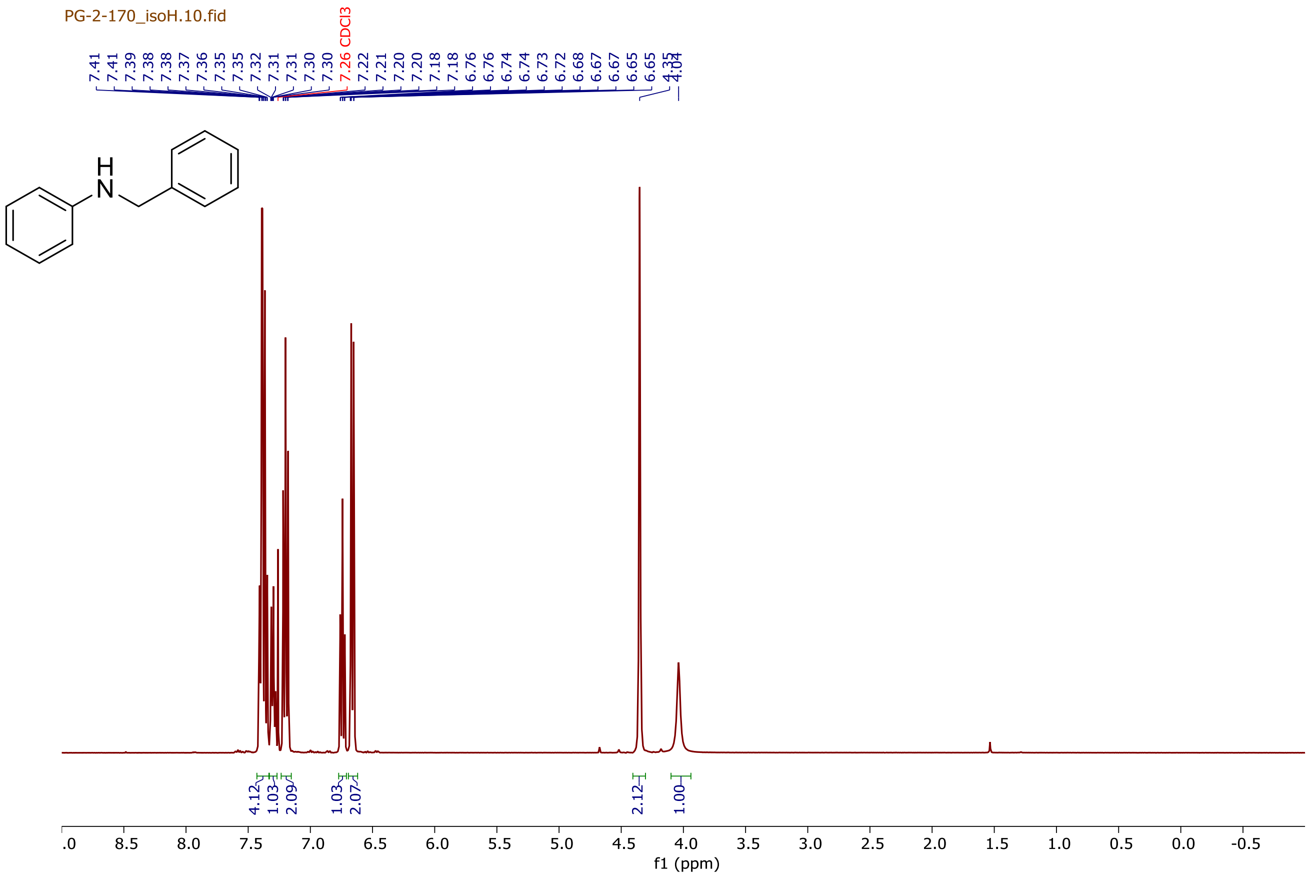

^{13}C NMR (101 MHz, $CDCl_3$)

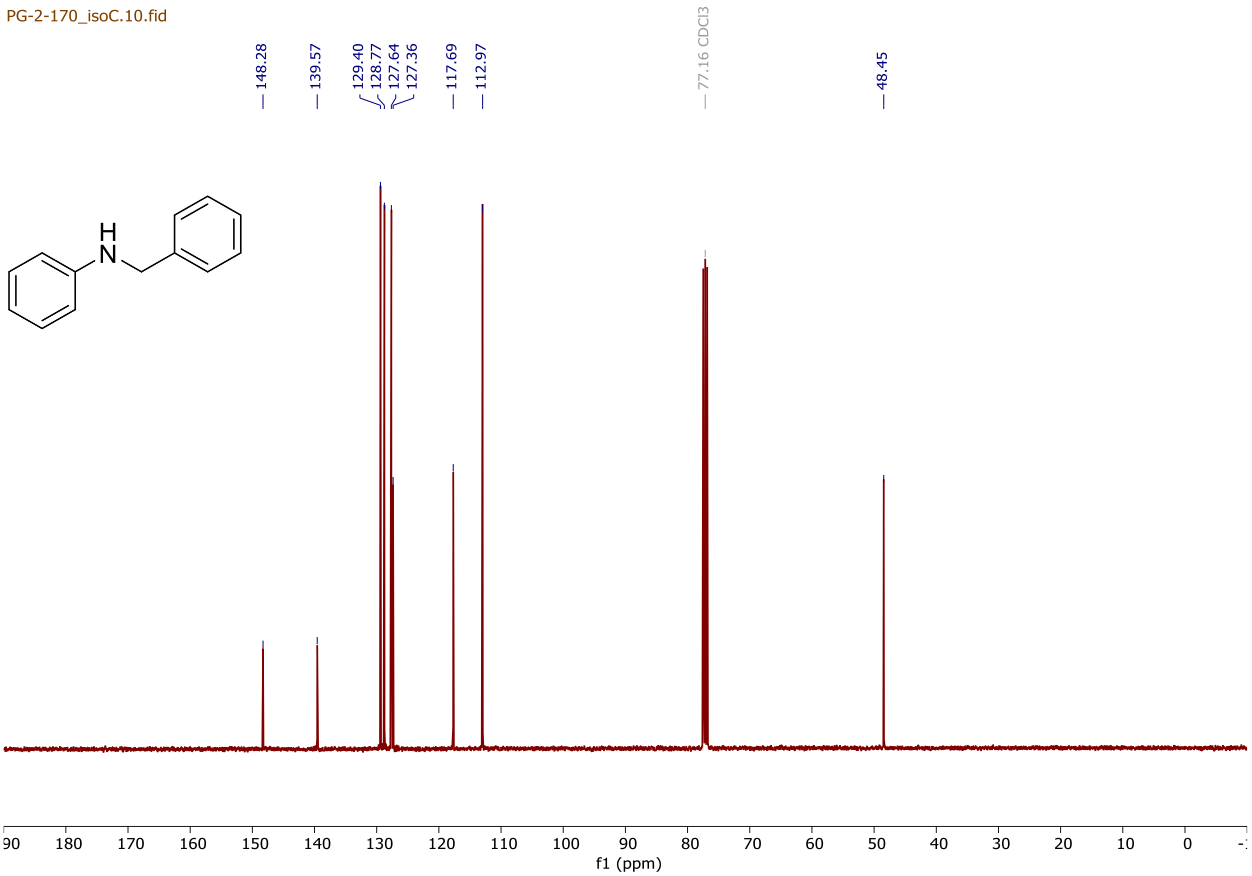

***N*-Methylaniline (17): ^{1}H NMR** (400 MHz, $CDCl_3$)

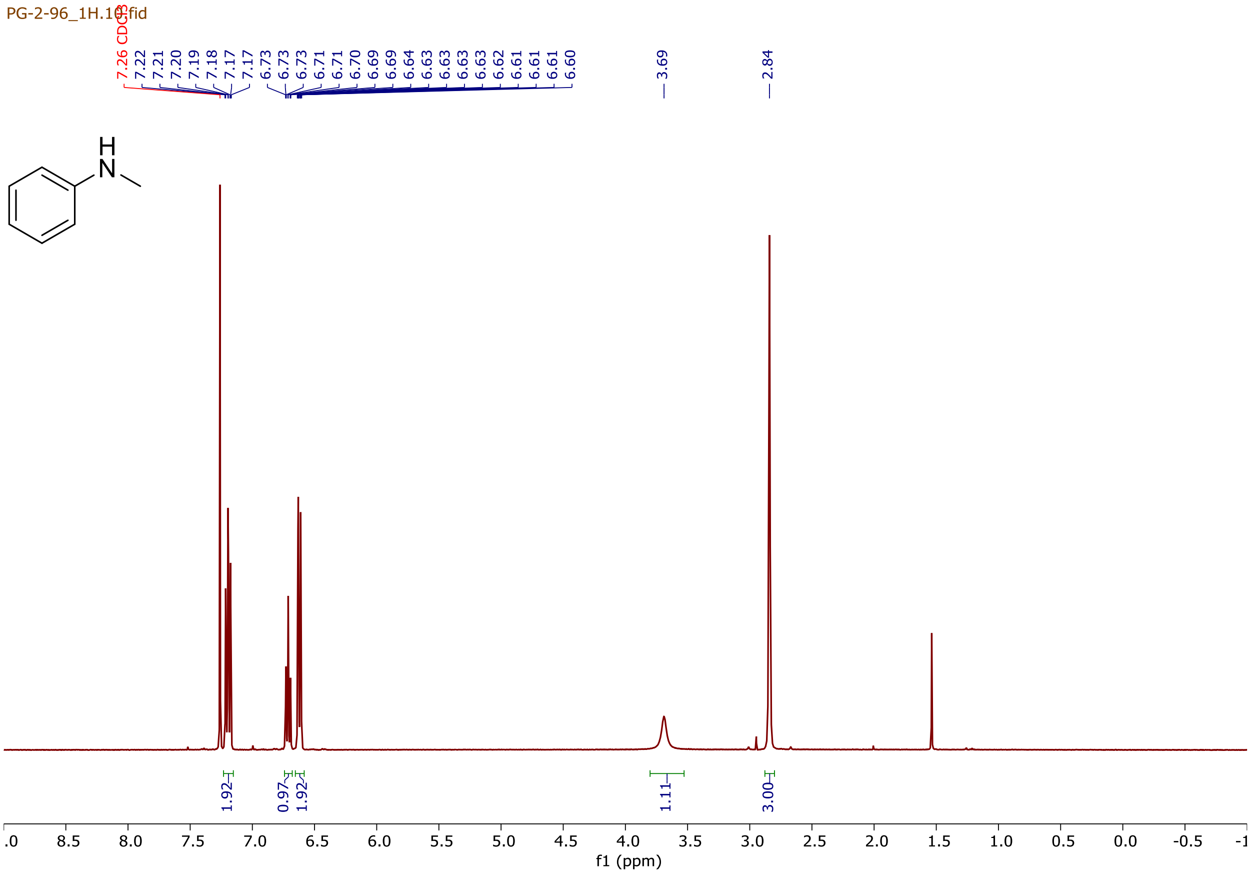

13C NMR (101 MHz, $CDCl_3$)

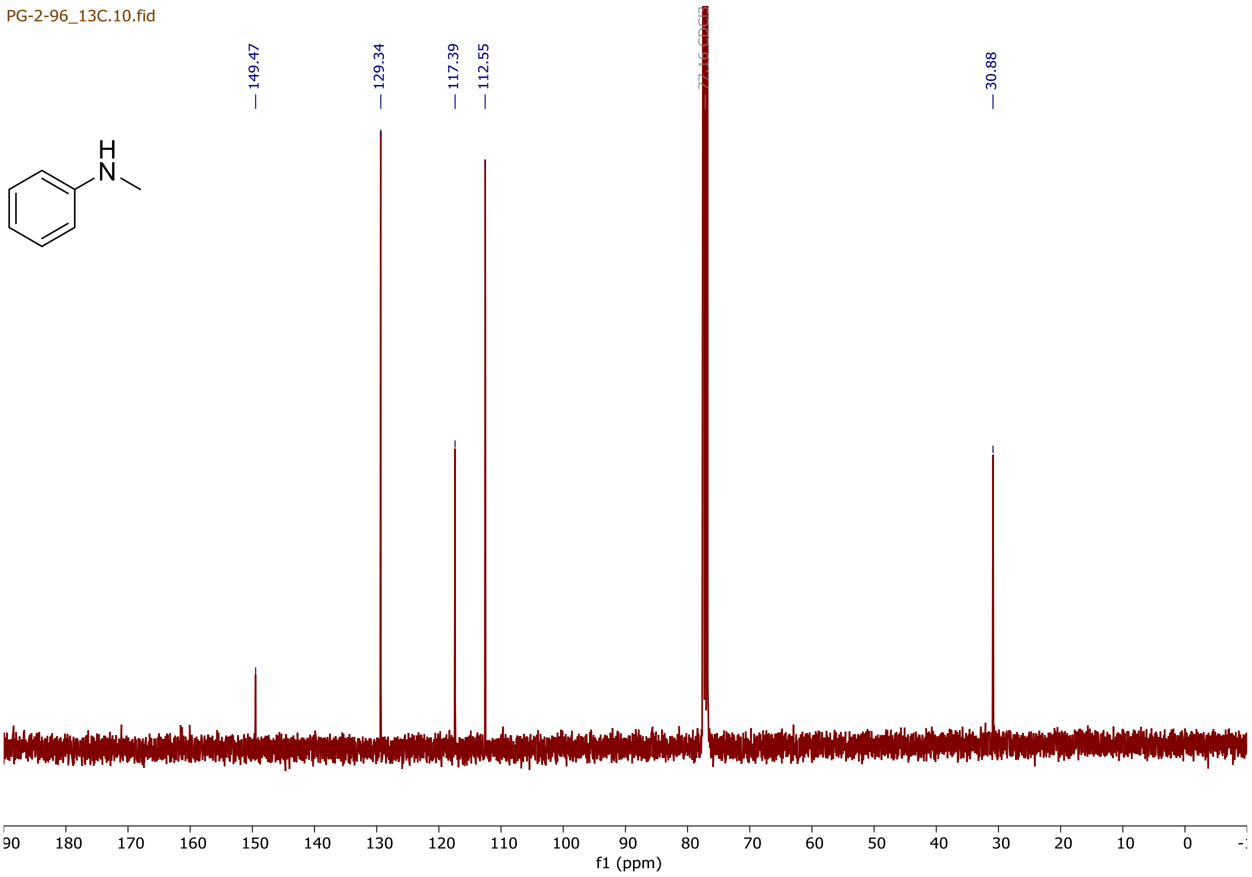

***N*-(2-(5-Methoxy-1*H*-indole-3-yl)ethyl)acetamide (18): ^{1}H NMR** (400 MHz, $CDCl_3$)

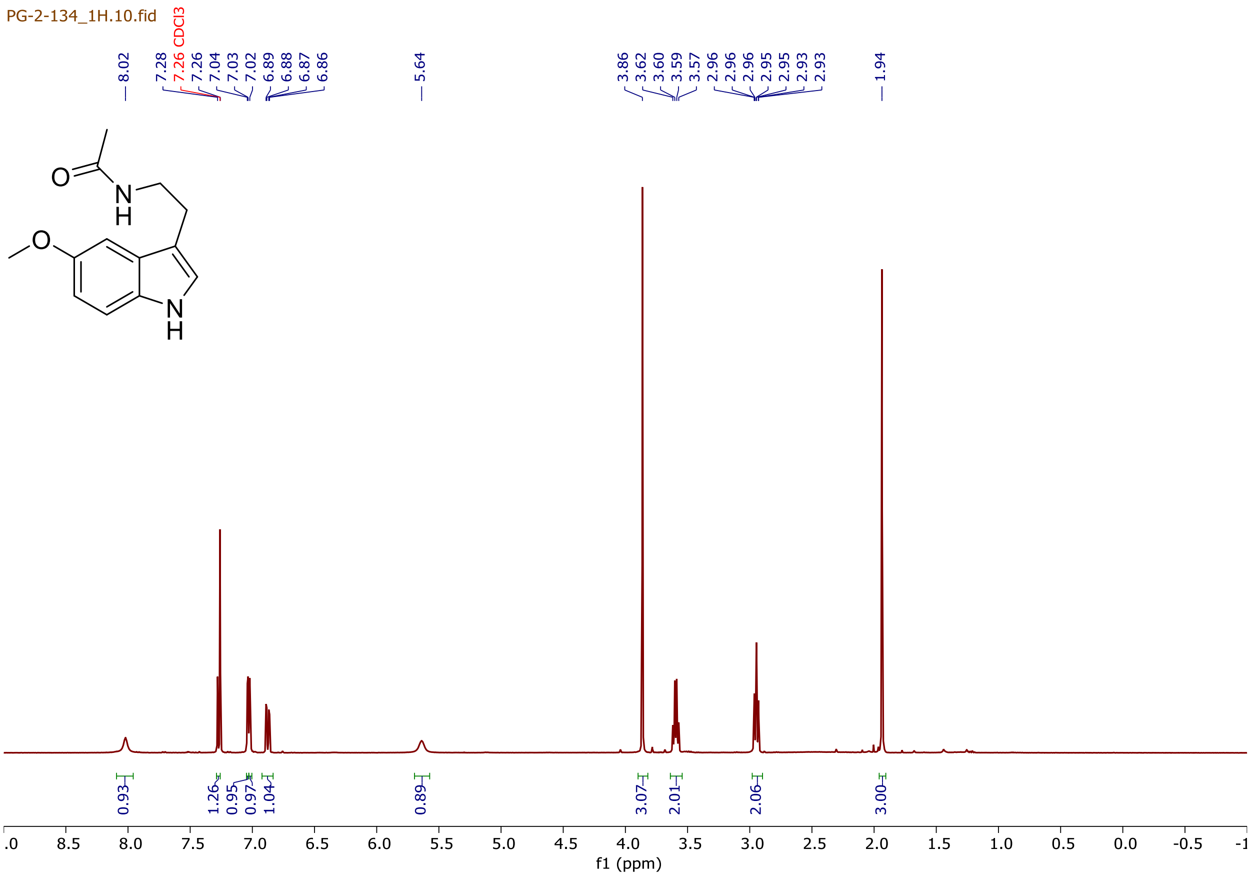

^{13}C NMR (101 MHz, $CDCl_3$)

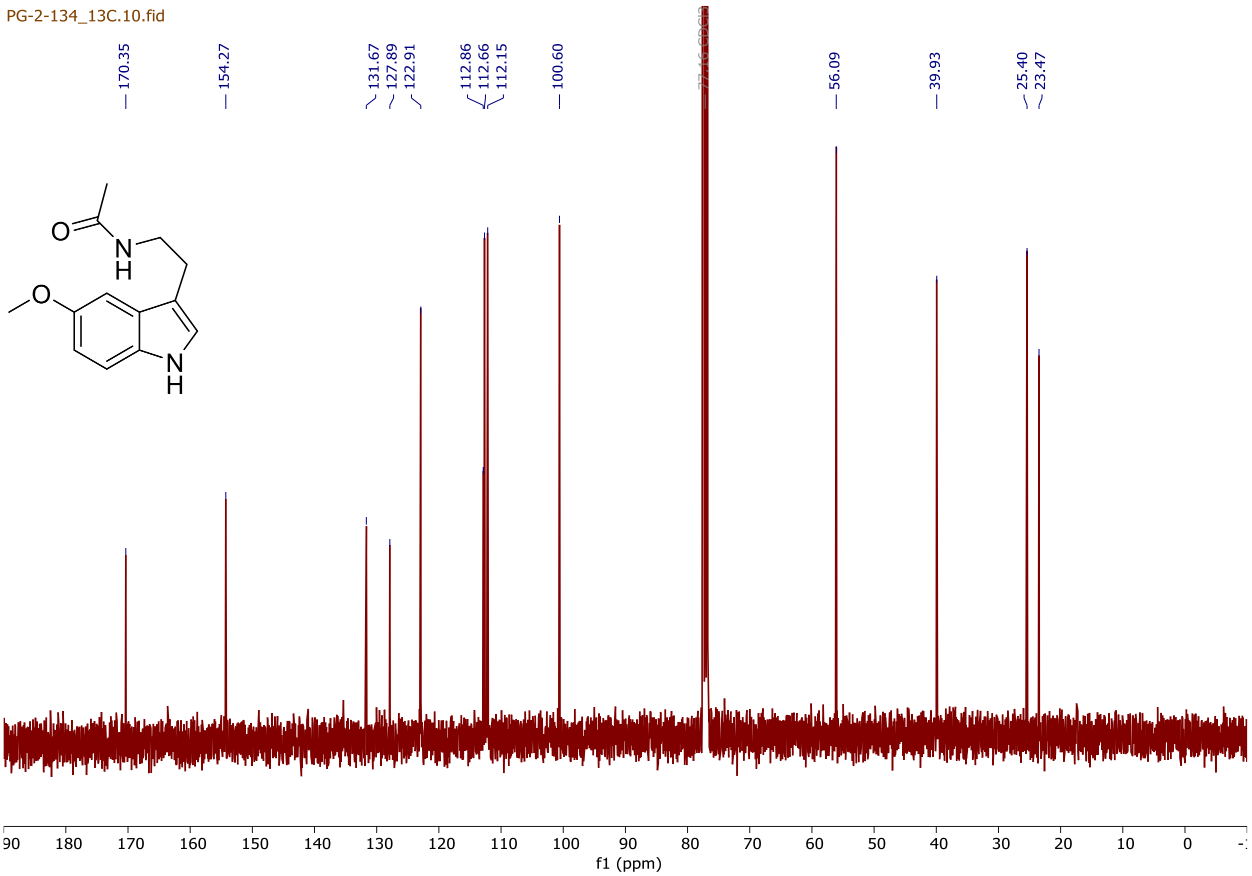

(*tert*-Butoxycarbonyl)tryptophan (19): ^{1}**H NMR** (400 MHz, Acetone-d_6)

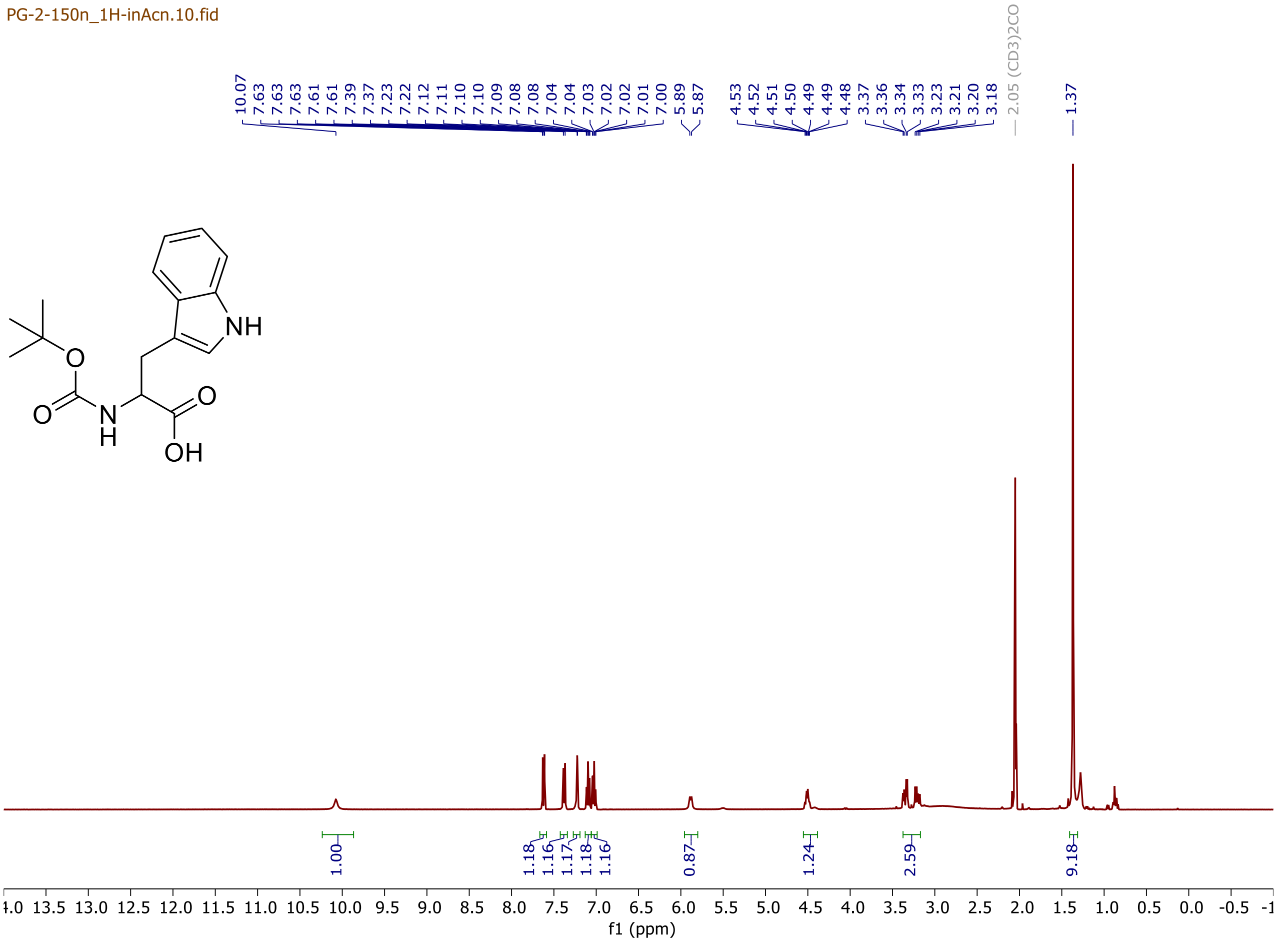

^{13}C NMR (101 MHz, Acetone-d_6)

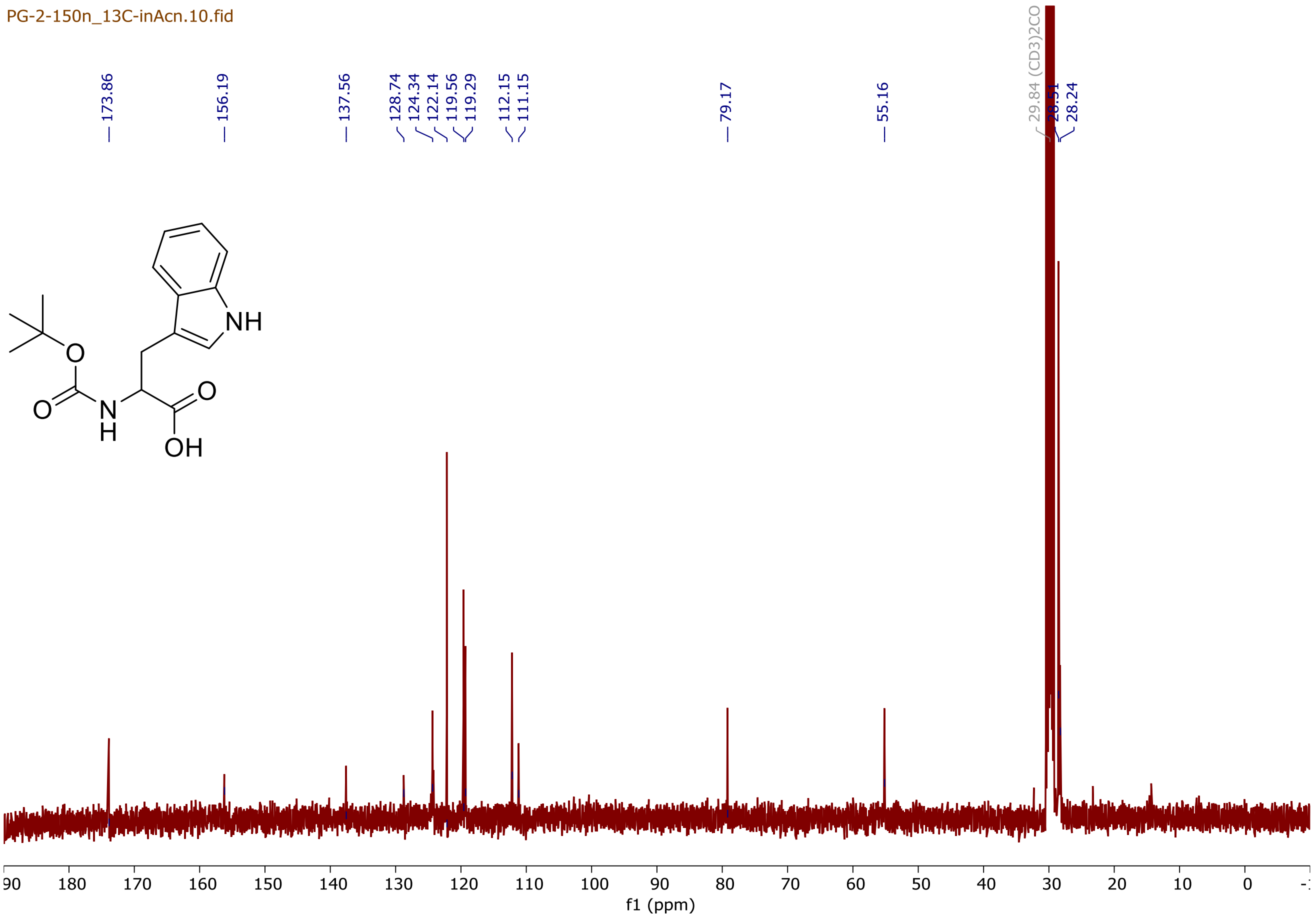

((Benzyloxy)carbonyl)tryptophan (20): ^{1}H NMR (400 MHz, CD_3OD)

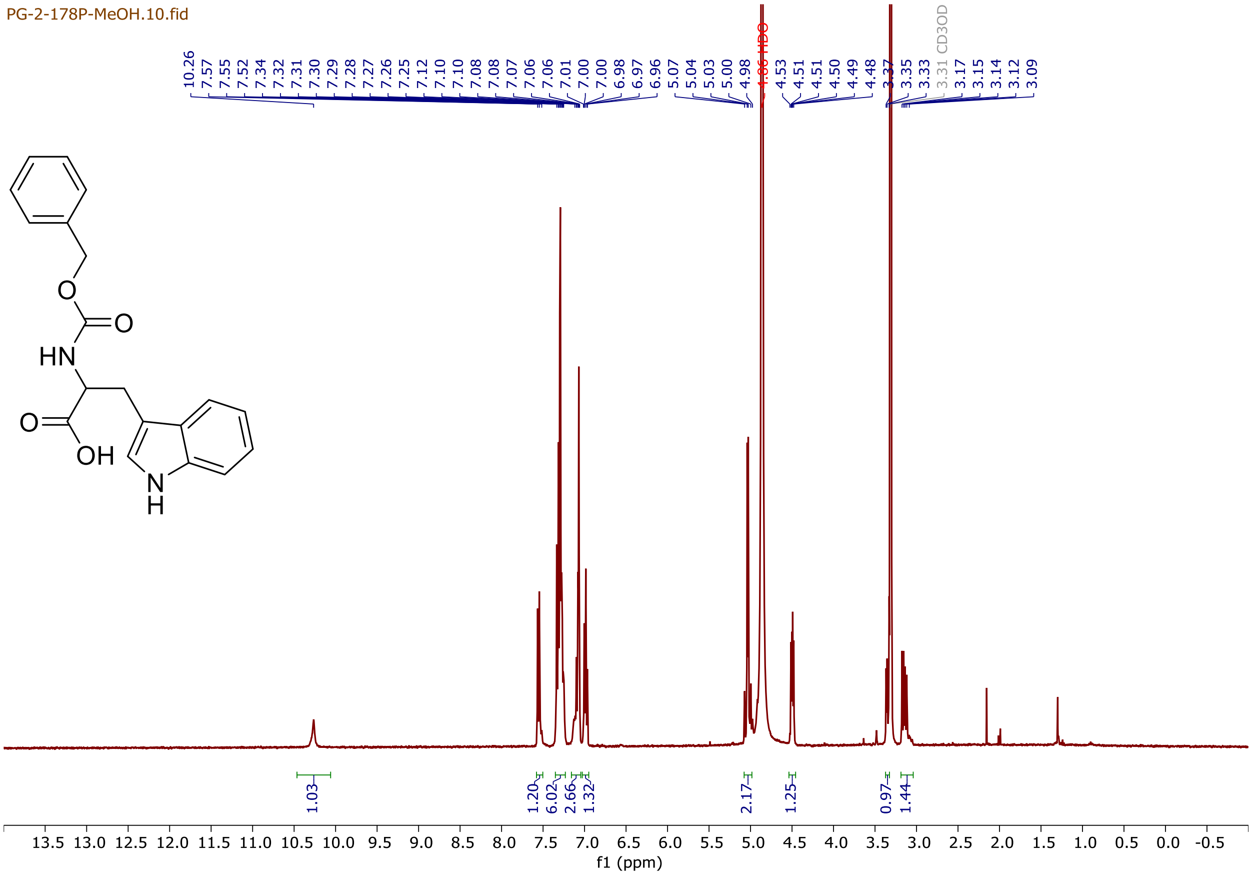

^{13}C NMR (101 MHz, CD_3OD)

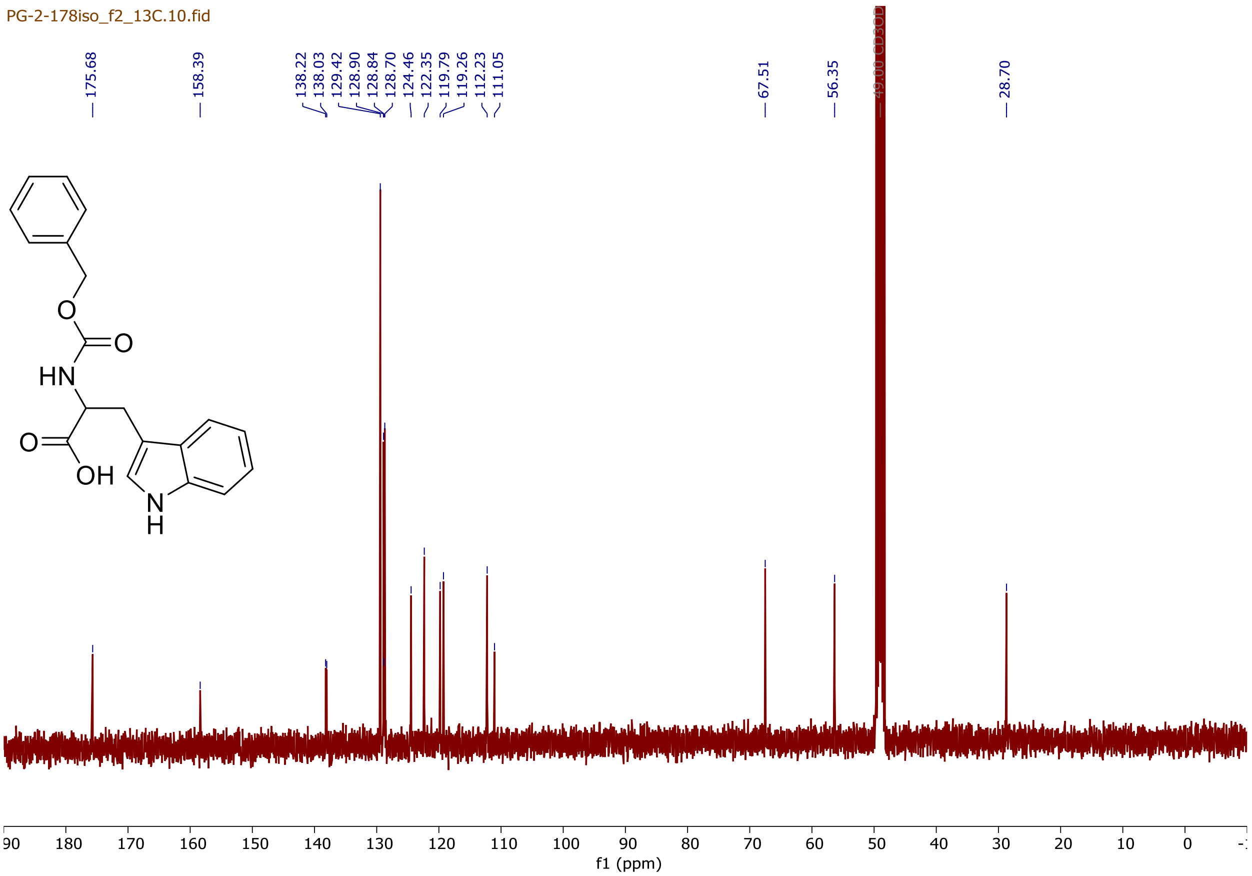

Aniline (21): ^{1}H NMR (400 MHz, $CDCl_3$)

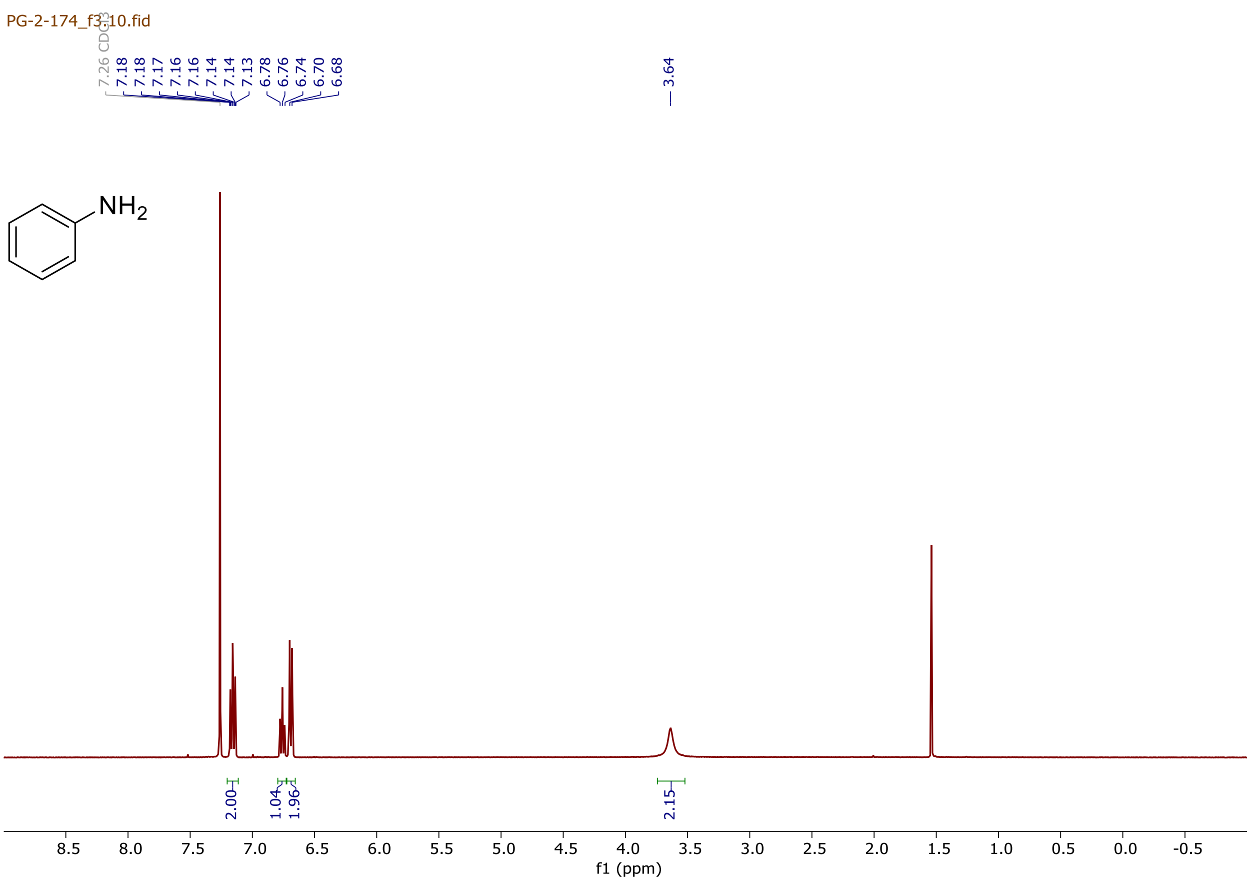

13C NMR (101 MHz, $CDCl_3$)

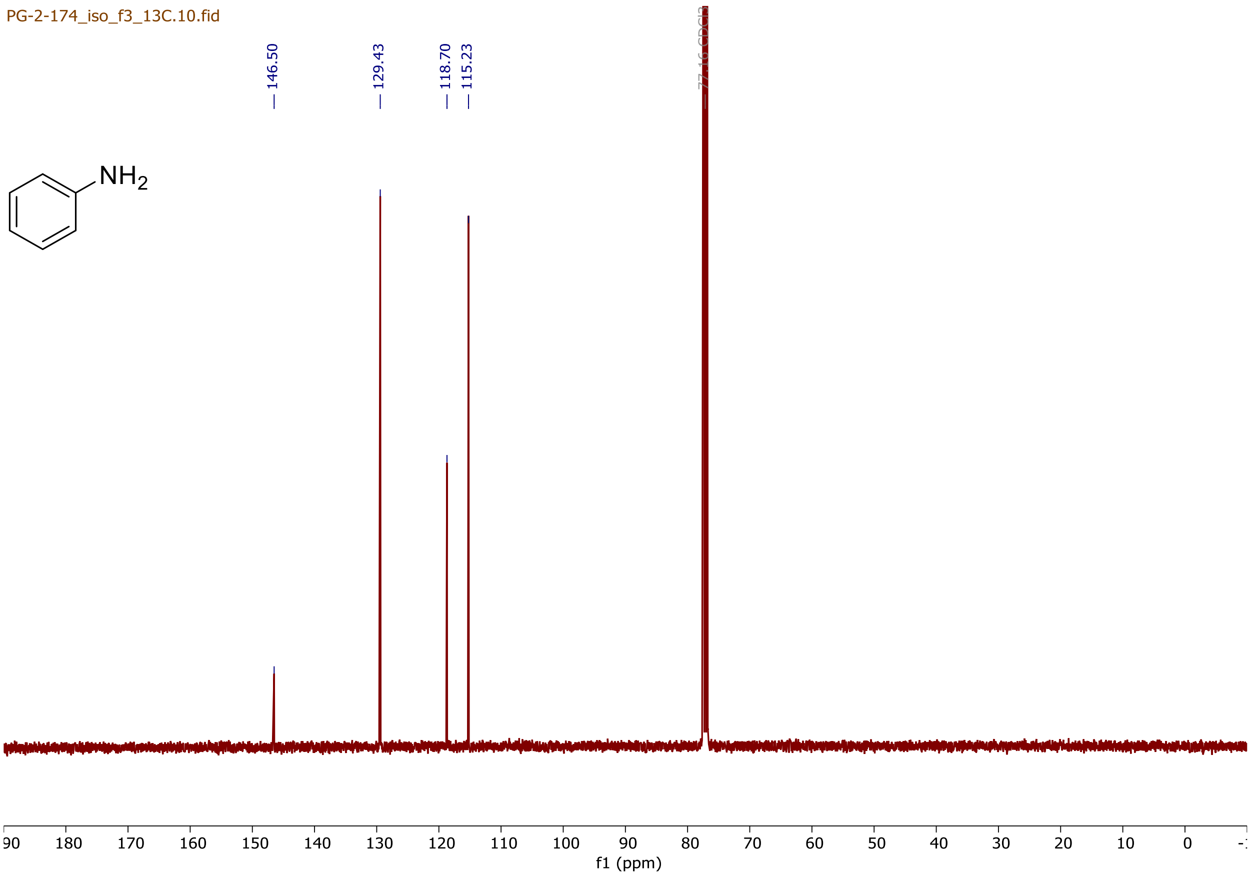

Benzyl propionate (22): ^{1}H NMR (400 MHz, $CDCl_3$)

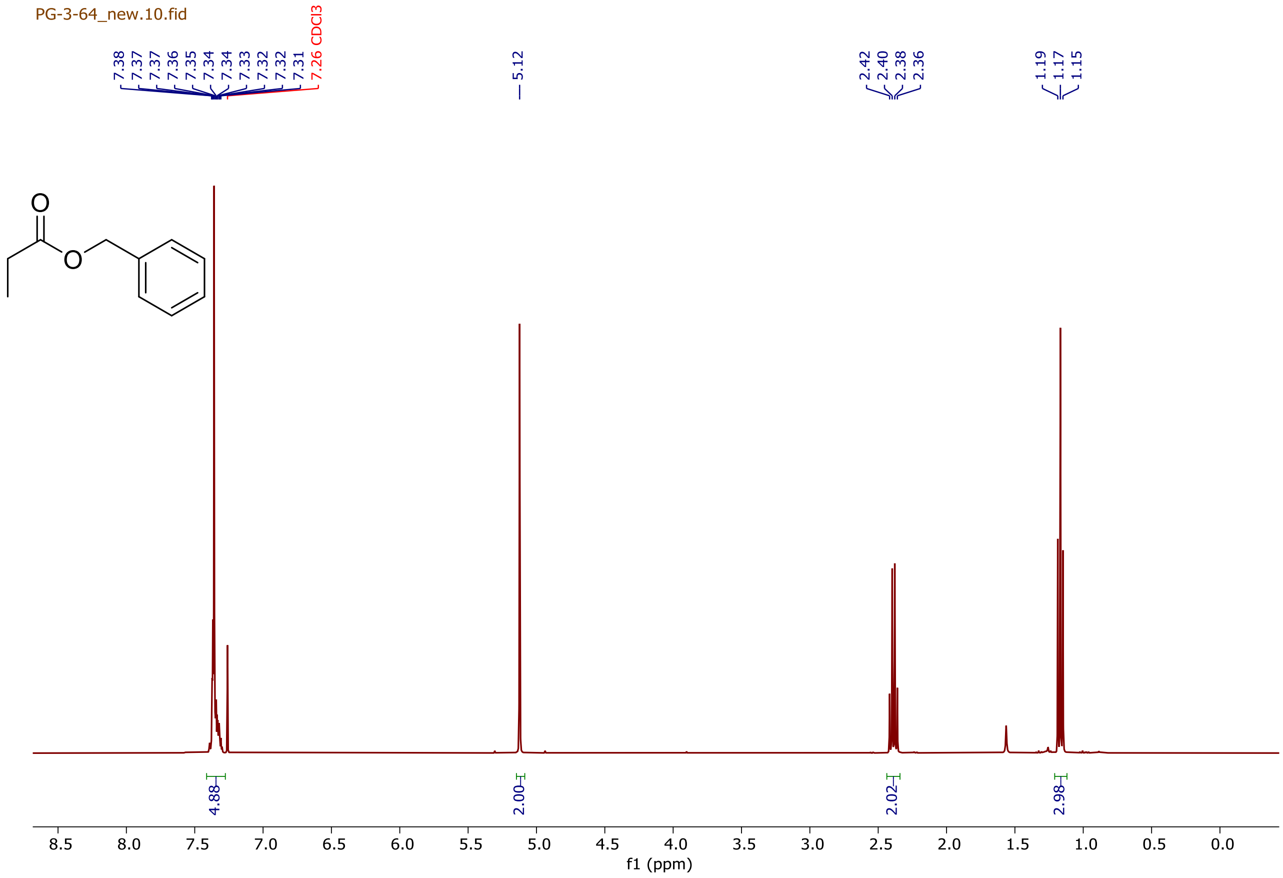

^{13}C NMR (101 MHz, $CDCl_3$)

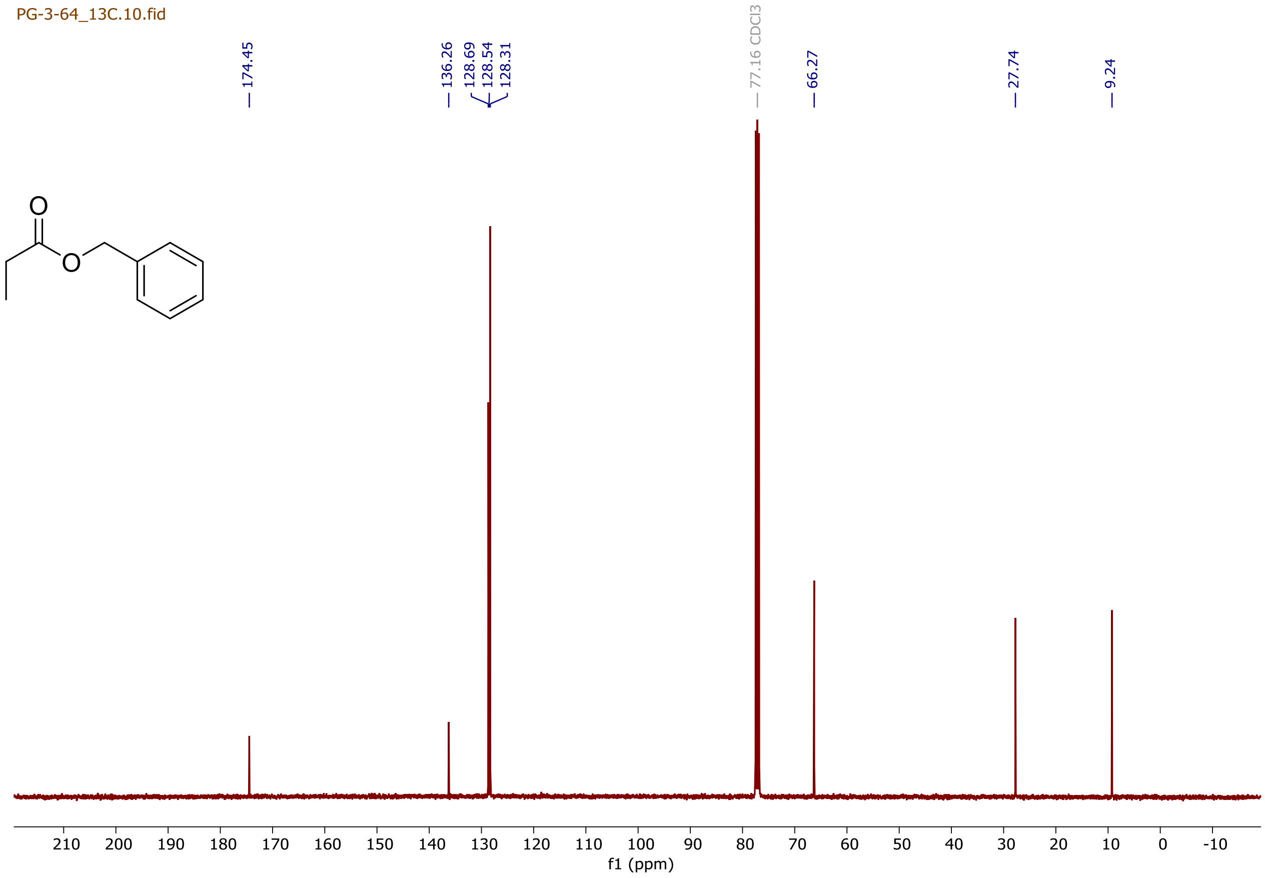

Benzyl isobutyrate (23): ^{1}H NMR (400 MHz, $CDCl_3$)

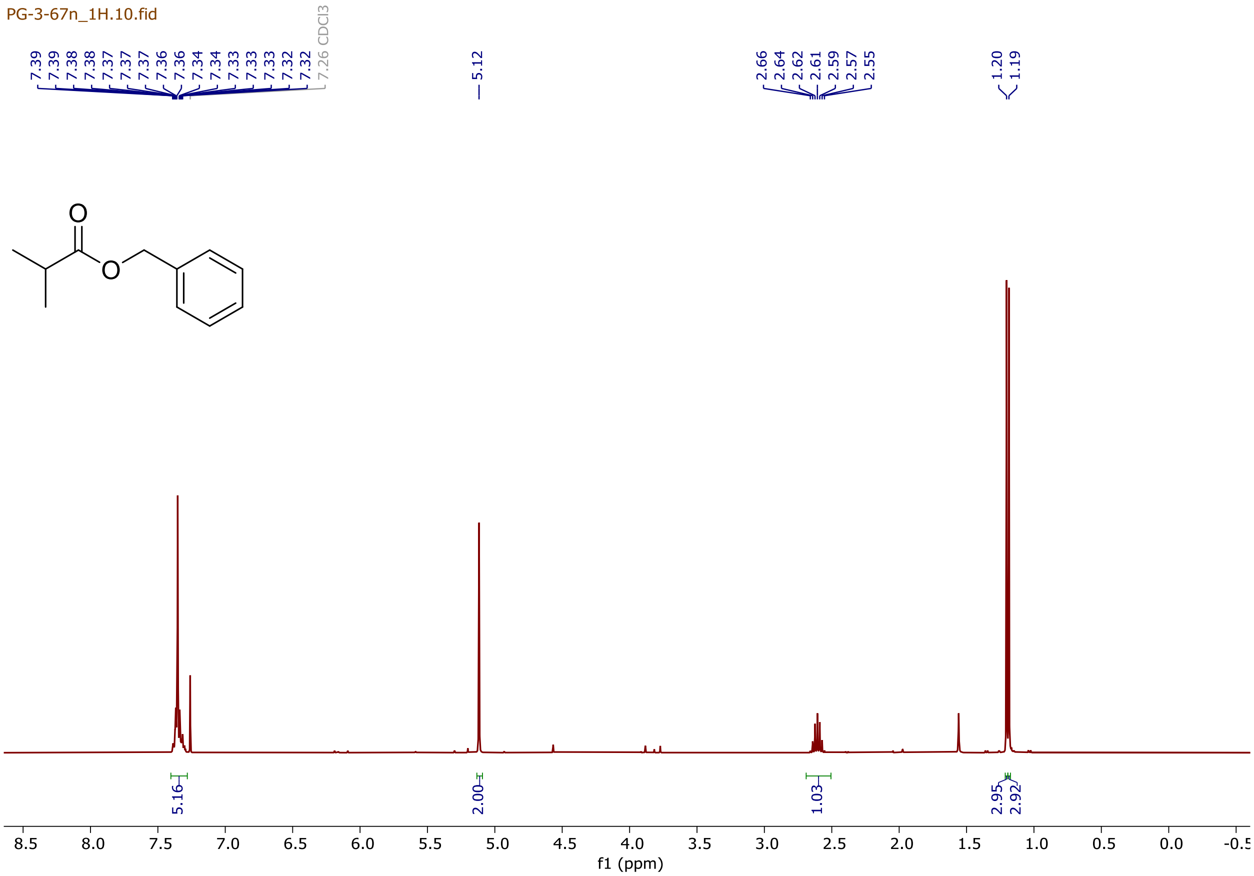

^{13}C NMR (101 MHz, $CDCl_3$)

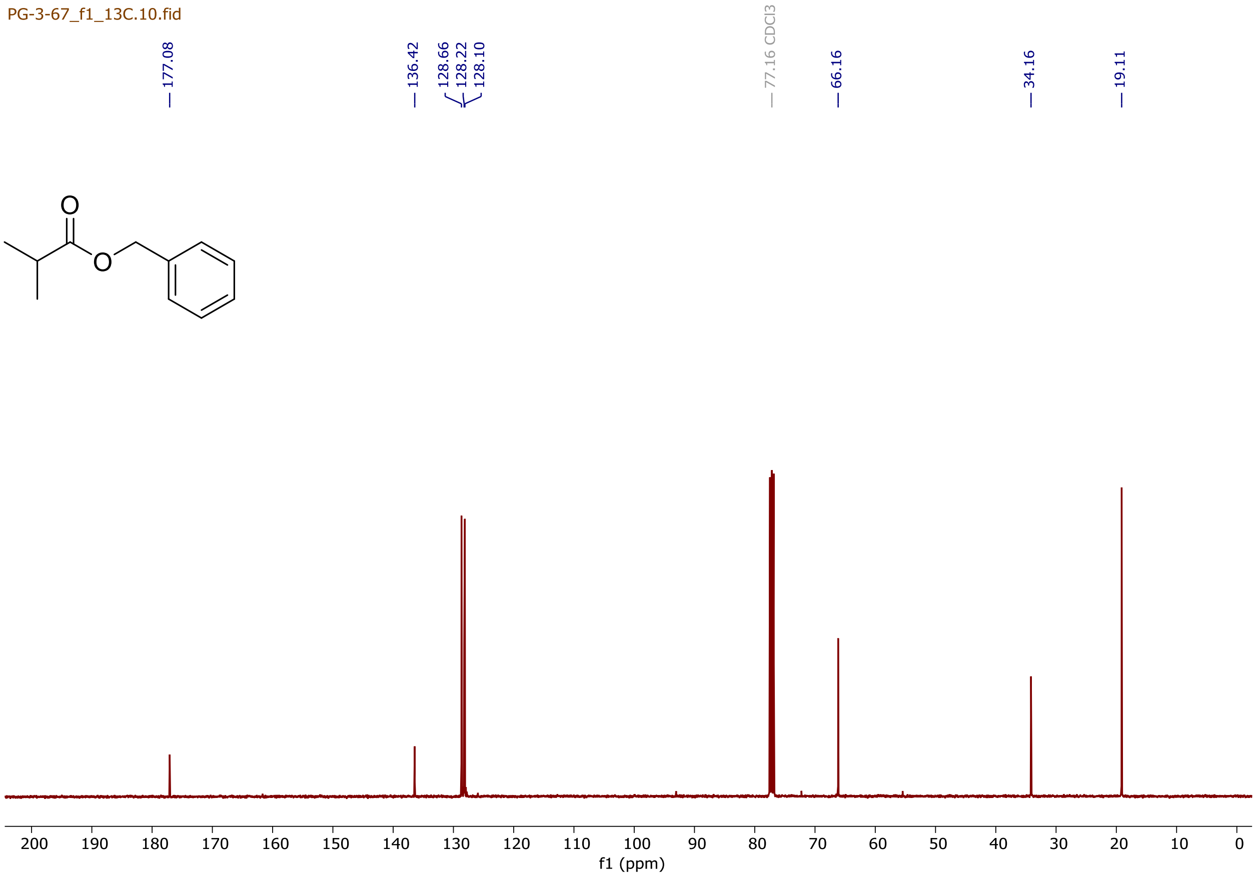

(Ethylsulfonyl)benzene (24): ^{1}H NMR (400 MHz, $CDCl_3$)

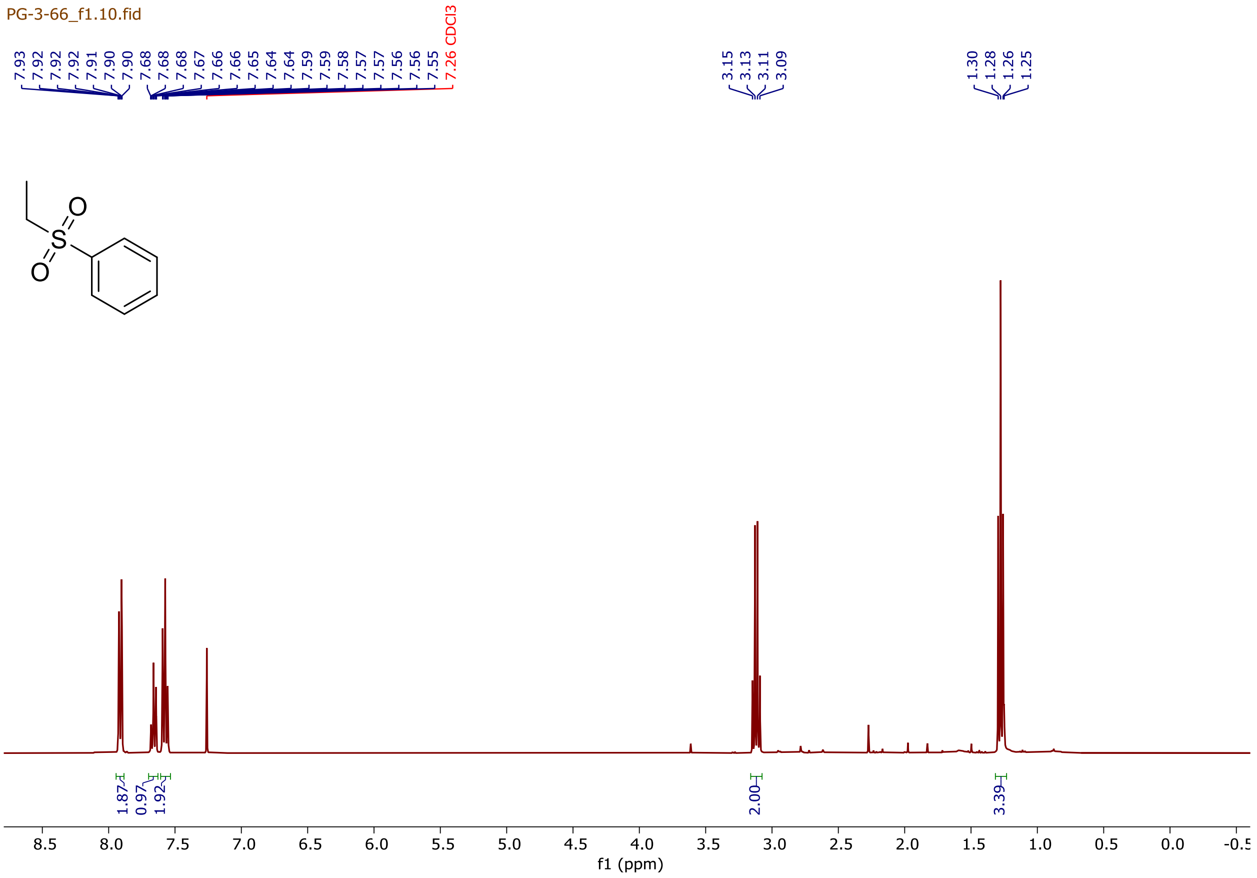

^{13}C NMR (101 MHz, $CDCl_3$)

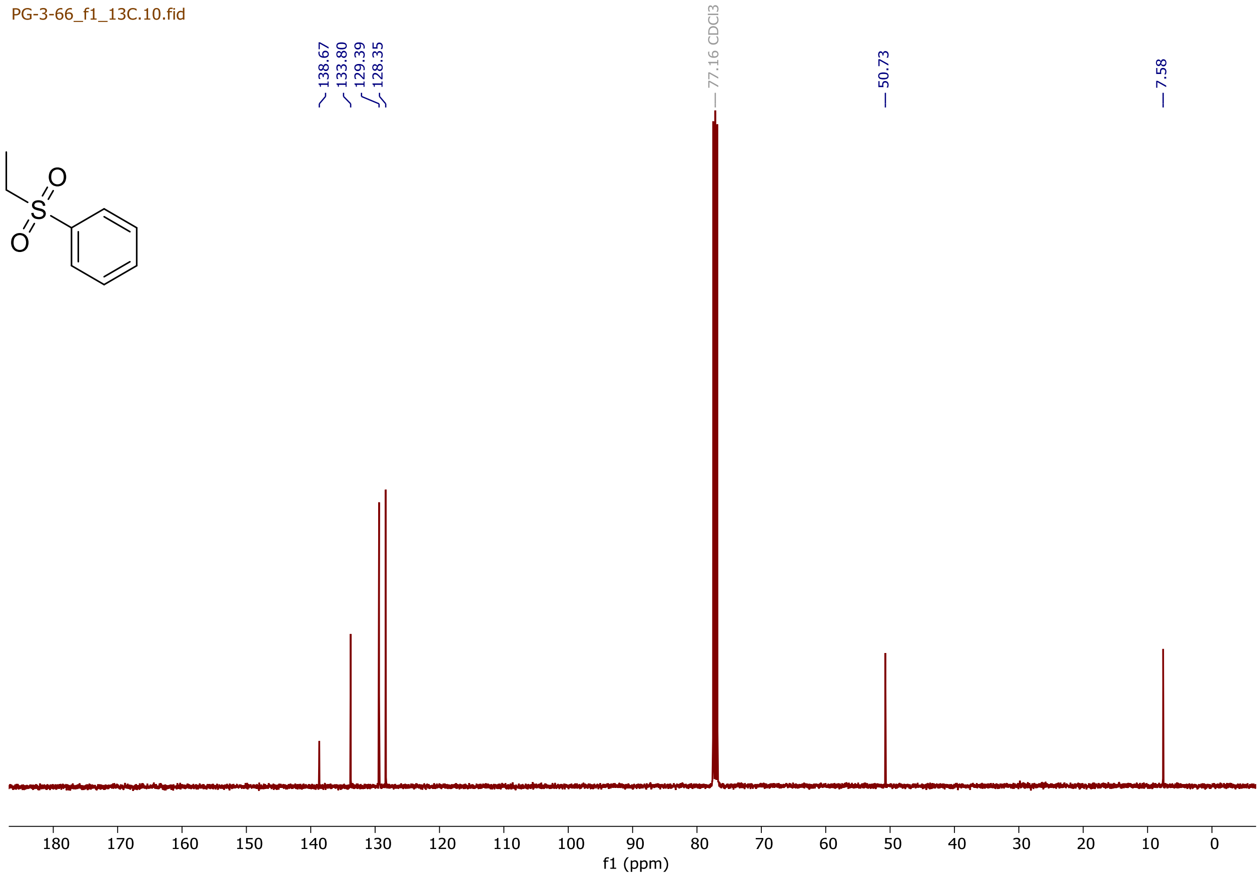

***N*-phenylpropionamide (25): ¹H NMR** (400 MHz, $CDCl_3$)

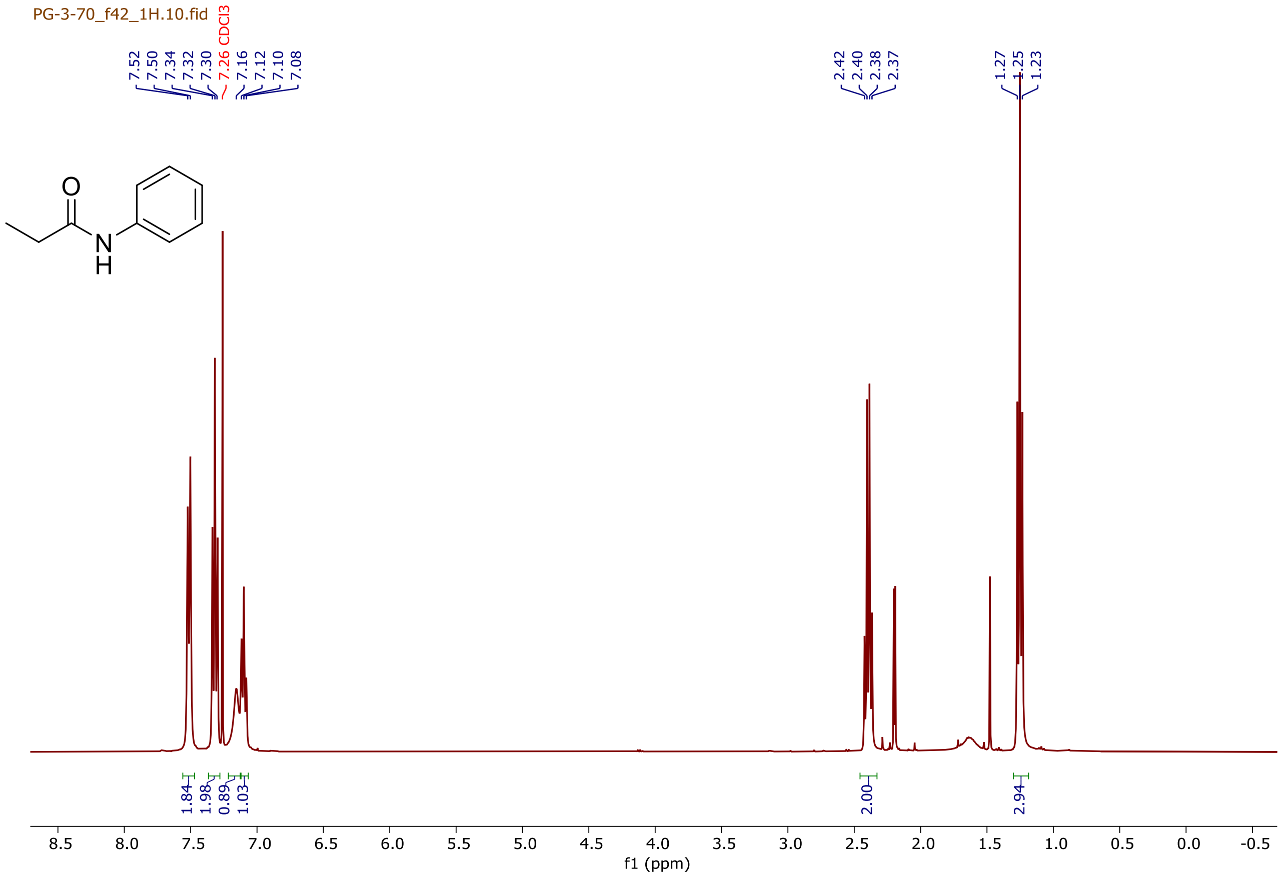

^{13}C NMR (101 MHz, $CDCl_3$)

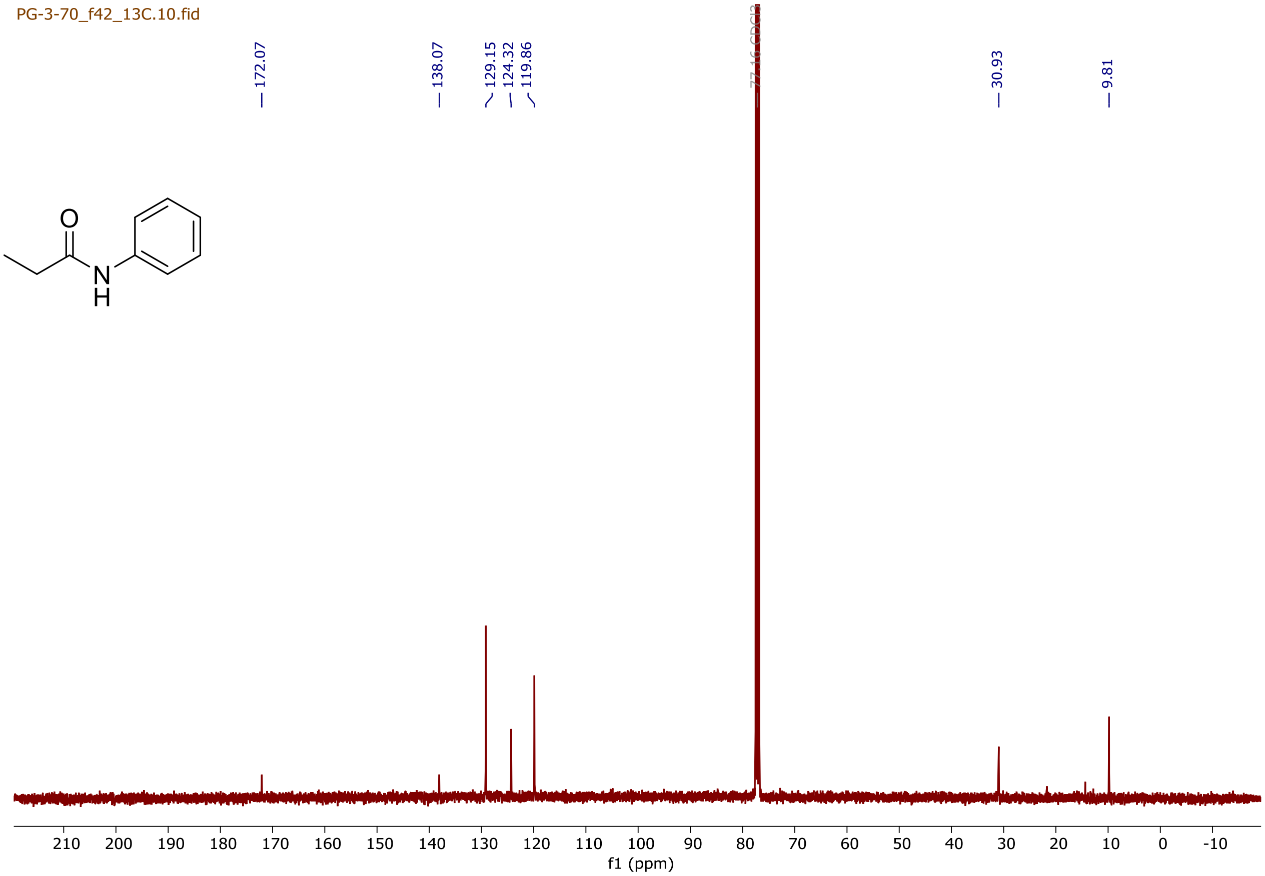

Supplement: Supplementary file 1 — ol4c00513_si_001.pdf [file ol4c00513_si_001.pdf]
